# Supplementary material for: Ivory Coast without ivory: Massive extinction of African forest elephants in Côte d’Ivoire
Source: PLoS One. 2020 Oct 14;15(10):e0232993. doi: 10.1371/journal.pone.0232993 (PMC7556483; doi:10.1371/journal.pone.0232993)
Supplement: S1 Appendix — (PDF) [file pone.0232993.s001.pdf]

S1 Appendix : Data collection sheets

Date : ...... /…… / ……..   Site : …………………………………….. N° Transect :……… Starting point: ……………………….

GPS coordinates departure:……………..… /……..……… Departure direction:……...... Weather:………… (Sunshine / forecast / rainfall)

<u>Reporter</u>: ………………………… Members of the team: ……...…………………………………………...

Time	Distance walked (m)	Type of vegetation (habitat)	Code of the observation	Type of observation	Number of object detected or group size	Stage or relative age index (nests / dung, etc.)	Geographical position				Perpendicular distance (m)
							GPS position N°	Longitude (UTM)	Latitude (UTM)	Altitude (m)	

Dung stage: S1 : fresh and wet with odor ; S2 : fresh dung, dry at surface and not wet, not odorous; S3 : 50% of the dung is decomposed, the dungs are decomposed and form a more or less flattened mass; S4 : dung piles are completely decomposed, the fecal matter is gone.
