# Supplementary material for: Ivory Coast without ivory: Massive extinction of African forest elephants in Côte d’Ivoire
Source: PLoS One. 2020 Oct 14;15(10):e0232993. doi: 10.1371/journal.pone.0232993 (PMC7556483; doi:10.1371/journal.pone.0232993)
Supplement: S2 Appendix — (PDF) [file pone.0232993.s002.pdf]

N° :…

S2 Appendix : Elephant survey questionnaire

Investigator :……………………………………………………………………………………….
Informant's name:……………………..……………………..…Date…………………………..
Home :…………… …………………. Place of investigation:……………………………….
Profession :………………………………. Village / Camp :…………………………………...
Plantation situation:…………………………………………………………………………….
Coordinates (GPS) of the plantation or the survey site :……………………………………….
Sub-prefecture:………………………… Prefecture:……………………………………………

01- Do you have problems with wild animals ?...

02- Which species?...

03- Have you ever seen elephants ?..

04- How many were they?...

05- How many groups were there?..

05- How many individuals per group? ………………………………………………………..

06- What is their size? (put on or in relation to the size of the man).....................................

07- Where did you see them?...

08- When did you see them? ....

	1st time	2nd time	3rd time
a -When precisely (month, week)			
b- Where?			
c- Have you heard of it?			
d- Have you seen any?			
e- Have you seen traces of it?			
f- How many were they (number)?			

09- In which regions can you easily meet Elephants?..

10- Why do you think they are common in these regions?..

11- Elephants are said to cause a lot of damage in plantations; did they arrive in your plantation ?...

12- Where is your plantation located in relation to the Reserve?...

13- How far is your plantation from the Reserve?...

14- How many times have elephants come to your plantation?...

15- Where do they come from?...

16- Where will they go next?...

17- How many were there? (each time)…………………………………………………

18- What were they doing ? (each time)……………………………………………………….

19- How many males were there? (each time)…………………………………………….

20- How many females were there? (each time)..

21- How many young people were there? (each time)..

22- How many cubs were there? (each time)..

23- Which were in front? (each time)………..

24- Which were behind? (each time)…………………..…………………………………………

25- Which were in the middle? (each time)..

26- Are there elephants that characterize these herds?.............................. ……………..

27- What are their characteristics (number, size, color, shape of ears, shape of tusks, etc.)?

……………………………………………………………………………………………

……………………………………………………………………………………………

28- When do the Elephants arrive on your plantation? (each time):

Saison	Clear moon	Black moon	Morning	Afternoon	Night (evening)	Night (morning)	Duration	Number of times
a. Rainy								
b. Dried								
c. Harvest Period								

29- Other (explain, list) : ………………………………………………………………..

……………………………………………………………………………………………….

30- Why do Elephants come here (to your home)?...

……………………………………………………………………………………………..

31- What crops do elephants destroy (list them in order of importance in relation to areas or productions)?

Season	Crops destroyed by elephants					
Rainy						
Dried						
Harvest period						
Quantity before damage (good, average, poor)						
Crop maturity (young, inter, mature)						

	Area / Production						
Cultures							
Total area							
Area destroyed							
Production obtained (t)							
Expected production (t)							
Expected income (FCFA)							
Loss in FCFA or in tonnes							

32- Other damage

a- Food reserve	
b- Water reserve	
c- Threat to human life	
d- Human injury	
e- Death of a man	
f- Others (specify)	
g- Injury or death Elephant	

33- Have you filed any complaints?..

Report No.	1	2	3	4	5

a- Sent ? Yes /No					
b- whose?					
c- When ?					
d- Where?					
e-How ?					

34- Have you received responses to your complaints (yes / no)?...

Report No.	1	2	3	4	5
a- Results obtained?					
b- Satisfied?					

35- Are elephants coming to your neighbors' plantations?..

36- When? (each time)……………………………………………………………………..

37- Where do they come from?...

38- Where will they go next?..

39- What methods do you use to keep elephants away from your plantations?.........................

……………………………………………………………………………………………..

……………………………………………………………………………………………..

40- Are these methods effective?...

41- Why ?...

42- What is the time used?...

43- What are the means used?

a- Humans……………………………………………………………………….

b- Equipment ………………………………………………………………………

c- Others……………………………………………………………………………

44- Do you know other methods that you have not yet experienced?

a- Yes/ Non…………………………………………………………………………

b- Which ?..

45- Are any of your non-crop property destroyed by elephants?

a- Lesquels						
b- When ?						
c- Value / cost						

46- Did you know that the Elephant plays an important role in nature for humans?...............

47- Cite examples you know

……………………………………………………………………………………………..

……………………………………………………………………………………………..

……………………………………………………………………………………………..

48- Are you in favor of elephant conservation?...

……………………………………………………………………………………………..

49- What advice can you give us to protect your crops?..

…………………………………………………………………………………………….

…………………………………………………………………………………………….

50- How can you help us achieve these goals?

…………………………………………………………………………………………….

…………………………………………………………………………………………….

51- What do you know about elephant behavior?

…………………………………………………………………………………………….

……………………………………………………………………………………………..

52- Are there other animals that destroy your crops?..

53- List them in order of importance ……………………………………………………

………………………………………………………………………………………………

………………………………………………………………………………………………

54- When do they destroy your crops ?...

……………………………………………………………………………………………….

………………………………………………………………………………………………..

55- To whom do you complain about the destruction of your crops (by other animals)?

………………………………………………………………………………………………..

56- How do they help you solve these problems?..

………………………………………………………………………………………………..

…………………………………………………………………………………………………..
57- Are you satisfied with the solutions they provide to you?..
…………………………………………………………………………………………………..
………………………………………………………………………………………………..
58- Have you ever complained to the managers of these animals?.......................................
59- How many times?..
60- What did they do?...
…………………………………………………………………………………………………..
…………………………………………………………………………………………………..
61- Were you satisfied?...
62- Why ?...
63- What would you like them to do?...
…………………………………………………………………………………………………..
………………………………………………………………………………………………….

64- What do you blame the government on this elephant problem?.......................................
…………………………………………………………………………………………………..
………………………………………………………………………………………………….
65- What do you blame yourself?...
…………………………………………………………………………………………………..
66- What are the paths taken by the Elephants to arrive:

a- in your plantations?..

b- in the Reserve?..

67- Do you know a story about these animals (movements, problems caused, etc.)?
…………………………………………………………………………………………………..
…………………………………………………………………………………………………..
68- Has the presence of these elephants changed:

a- The speculations practiced?………………………………..……………………..

b- Your sowing dates (crop calendar)?…………………………..…..……………….

c- Your agricultural practices?………………………………….……………………….

d- Your eating habits?………………………………...………………………………..

e- Your lifestyle in general?…………….………………………..…………………….

…………………………..……………………………………………………………………..

<u>Other</u>

<u>information</u>…………………………………………………………………..…………………….

……………………………………………………………………………………..……………….

……………………………………………………………………………………………………….

………………………………………………………………………………………………………..

………………………………………………………………………………………………………..

………………………………………………………………………………………………………..

………………………………………………………………………………………………………..

………………………………………………………………………………………………………..

………………………………………………………………………………………………………..

………………………………………………………………………………………………………..

………………………………………………………………………………………………………..

………………………………………………………………………………………………………..
