# Supplementary material for: Ivory Coast without ivory: Massive extinction of African forest elephants in Côte d’Ivoire
Source: PLoS One. 2020 Oct 14;15(10):e0232993. doi: 10.1371/journal.pone.0232993 (PMC7556483; doi:10.1371/journal.pone.0232993)
Supplement: S3 Appendix — (PDF) [file pone.0232993.s003.pdf]

S3 Appendix : Supporting data

Survey site	Size (km^2)	Presence / Absence of elephant	% area converted to plantation	Forest converted to cocoa farms (Km²)	Forest left (Km²)	Estimated annual cocoa production (tons)	Human Population size inside the forest	Poaching Index	Human population siz in the Department	Level of protection
Dassioko FR	79,8	10	80	63,84	15,96	63,8	0	1,1	207378	4
Port-Gauthier FR	25	5	80	20	5	20	0	1,1	207378	3
Bolo-Ouest FR	66,1	0	100	66,1	0	66,1	100	0,2	207378	1
Niégré FR	925	0	100	925	0	925	22	0,1	628592	1
Monogaga FR	396,6	0	100	396,6	0	396,6	24	0,04	422204	1
Marahoué NP	1,01	0	100	989,8	0	1010	30	0,7	236312	1
Rapide Grah FR	109,4	0	100	109,4	0	109,4	1200	0,15	628592	1
Haut Sassandra FR	102,4	0	70	71,68	30,72	71,7	1600	1,3	524214	1
Mont Péko NP	34	0	100	34	0	34	21,837	0,04	408148	1
Bouaflé FR	20,4	0	75	15,3	5,1	15,3	1600	0,9	236312	1
Kani-Bandaman Rouge FR	105,5	0	80	84,4	21,1	84,4	400	1,2	172358	1
Séguéla FR	119,2	0	97	115,62	3,57	115,6	600	0,8	172358	1
Koba FR	31,5	0	98	30,87	0,63	30,8	800	0,2	286182	1
Dé FR	13,5	0	80	10,8	2,7	10,8	1,2	0,4	148480	1
Haut Dé FR	7,4	0	95	7,03	0,37	7,1	1	0,7	286182	1
Moyenne Marahoué FR	38,3	0	90	33,7	3,83	34,5	2	0,9	286182	1
Azagny NP	194	0	40	77,6	116,4	77,6	0	1,1	85981	2
Bossématié FR	220	16	20	44	176	44	0	1,8	288231	3
Mabi FR	598	0	20	119,6	478,4	119,6	0	1,7	288231	2
Yaya FR	241	0	10	24,1	216,9	24,1	0	1,4	288231	2
Banco NP	30	0	0	0	30	0	100	1,2	3125890	1
Ile Ehotilé NP	5,5	0	0	0	5,5	0	0	1	100445	1
N'Gadan-N'Gadan FR	20	0	0	0	20	0	0	0,09	100445	1
Mont Tia	249	0	95	70	5	25	0	1,6	366916	1
Taï NP	5082	189	2	0,06	98	5	0	0,4	176688	5
