# Supplementary material for: Ivory Coast without ivory: Massive extinction of African forest elephants in Côte d’Ivoire
Source: PLoS One. 2020 Oct 14;15(10):e0232993. doi: 10.1371/journal.pone.0232993 (PMC7556483; doi:10.1371/journal.pone.0232993)
Supplement: S1 File — (PDF) [file pone.0232993.s004.pdf]

REPUBLIC OF CÔTE D'IVOIRE

Union, Discipline, Labor

Law n ° 96-766 of October 3, 1996

on the Environmental Code

THE NATIONAL ASSEMBLY, adopted,

THE PRESIDENT OF THE REPUBLIC, promulgates the law, the content of which follows:

TITLE 1: DEFINITIONS, OBJECTIVES AND FIELD OF APPLICATION

CHAPTER I: DEFINITIONS

Article 1:

Under the terms of this law:

The environment is the set of physical, chemical, biological elements and socio-economic, oral and intellectual factors likely to have a direct or indirect, immediate or long-term effect on the development of the environment, living beings and activities human.

The human environment concerns the living environment and land use planning.

The natural environment includes:

- the ground and the basement.
- water resources.
- the air ;
- biological diversity ;
- landscapes, sites and monuments …

Water resources include inland surface water and groundwater.

Air is the atmospheric layer whose physical, chemical or other modification can adversely affect the health of living beings, ecosystems and the environment in general.

The landscape is a portion of the territory whose various elements form a picturesque whole by the arrangement of its components or the contours of its forms or the effect of its colors.

The site is a portion of the landscape particularized by its geographical location and / or its history.

The natural monument is an element or a group of elements due to nature such as rocks, trees, sources, ground disturbances, geological accidents or others which, separately or together, form a panorama worthy of attention.

The ecosystem is a structured whole which encompasses the biotope and the biocenosis in one or the same functional unit.

The biotope is the geographical area where all the physical and chemical factors of the environment remain substantially constant.

The biocenosis is the set of plants and animals that live in the same environmental conditions and in a given space of varying dimensions.

Ecology is the study of the environments in which living beings live, reproduce and die, as well as the relationships of these beings with the environment and their protection against any pollution.

Biological diversity is the variability among living organisms from all sources including, inter alia, terrestrial, marine and other aquatic ecosystems and ecological complexes of which it is part; this includes diversity within species, between species and of ecosystems.

P**ollution** is the direct or indirect contamination or modification of the environment caused by any act likely to:

- alter the living environment of man and other living species ;
- harm the health, safety, well-being of man, flora and fauna or collective and individual goods.

Water pollution is the introduction into the aquatic environment of any substance liable to modify the physical, chemical and / or biological characteristics of the water and to create risks for human health, harm wildlife and terrestrial and aquatic flora, undermine the approval of the sites or hamper any other rational use of water.

Atmospheric pollution or air pollution is the deliberate or accidental emission into the atmospheric layer of gas, smoke or substances likely to create nuisances for living beings, to compromise their health or public safety or to harm to agricultural production, the conservation of buildings or the character of sites and landscapes.

Transboundary pollution is pollution that originates in one country and the effects of which spread to other countries.

Protected areas are areas specially devoted to the preservation of biological diversity and the natural resources associated with it.

The maritime zones include: the archipelagic waters, the territorial sea, the exclusive economic zone, the continental shelf as well as the sea shore, the seabed and the corresponding subsoil.

Human settlement includes all urban and rural agglomerations, the infrastructures and equipment they must have in order to ensure their inhabitants a pleasant living environment and a healthy, harmonious and balanced existence.

Hydrocarbons are energetic, fluid (liquid or gaseous) substances.

Nuisance is any harm to the health of living beings, whether or not they are done, by the emission of noise, light, odors, etc.

Waste is solid, liquid or gaseous products resulting from household activities, a manufacturing process, or any abandoned or threatened movable or immovable property.

Hazardous wastes are solid, liquid or gaseous products, which present a serious threat or particular risks to the health, safety of living beings and the quality of the environment.

Fertilizers are fertilizers, soil amendments and any product whose use contributes to improving agricultural productivity.

Natural hazards are natural disasters and calamities that can have unpredictable effects on the environment and health.

A major accident is defined as an event such as an emission of dangerous substances, a fire, an explosion resulting from an uncontrolled development of an industrial, agricultural or domestic activity.

Emergency plans are defined as the rapid and rational organization, under the responsibility of a determined authority, of all kinds of means to deal with an extremely serious situation.

Bush fires are fires ignited intentionally or not, regardless of the magnitude, causing damage to man and his property, flora and fauna.

Desertification refers to the degradation of land in arid, semi-arid and dry sub-humid areas as a result of various factors, including climatic variations and human **activities.**

Fishing consists of the capture, extraction or harvesting of fish, cetaceans, plant chelonians, plankton or vertebrate or invertebrate animals living partially or completely in the aquatic environment.

Hunting consists of any act tending

- to injure or kill in order to appropriate or not all or part of its remains, an animal in freedom in its natural environment within the meaning of the legislative and regulatory texts in force;

- destroy the eggs of birds and reptiles.

Capture consists of any act tending to:

- deprive of its freedom, a wild animal;

- collect and remove the eggs of birds or reptiles from their natural place of hatching.

The environmental impact study is an assessment report of the probable impact of a planned activity on the environment.

The Environmental Impact Study Office is a service available to the Authority Competent National responsible for examining impact studies.

The environmental audit is a procedure for evaluating and controlling actions to protect the environment.

The Competent National Authority is a single entity or a group of entities whose powers are defined by decree.

The Environmental Defense Association is the organization through which two or more people pool their knowledge or their activities with a view to contributing to the defense of the environment.

CHAPTER II: OBJECTIVES

Article 2

This code aims to:

- protect soils, subsoils, sites, landscapes and national monuments, plant formations, fauna and flora and particularly classified areas, national parks and existing reserves;

- establish the fundamental principles intended to manage and protect the environment against all forms of degradation in order to develop natural resources, to fight against all kinds of pollution and nuisances;
- improve the living conditions of the different types of population while respecting the balance with the surrounding environment;
- create the conditions for a rational and sustainable use of natural resources for present and future generations;
- guarantee all citizens an ecologically healthy and balanced living environment;
- ensure the restoration of damaged environments.
-

CHAPTER III: FIELD OF APPLICATION

Article 3

This law does not preclude the application of legislative and regulatory provisions relating to town planning and construction, health, hygiene, safety and public tranquility, the protection of ecosystems and in general to the exercise of police powers.

Article 4

This law does not apply to military activities and war situations. However, the authors of such activities are required to take into account environmental protection concerns.

Article 5

This law applies to all forms of pollution as defined in article 1 of this code and likely to cause an alteration in the composition and consistency of the atmospheric layer with harmful consequences for the health of living beings. , production, goods and the balance of ecosystems.

Article 6

Are subject to the provisions of this law;

- classified installations as defined in their nomenclature: factories, depots, mines, construction sites, quarries, underground or surface storage, stores and workshops;
- facilities operated or owned by any natural or legal person, public or private, which may present dangers or inconveniences, either for convenience or for health, safety and public sanitation;

- spills, flows, discharges and deposits' likely to cause or increase the degradation of the receiving environment.

Article 7

The following types of energy are referred to under this law:

- solar energy ;
- biomass energy ;
- wind energy ;
- geothermal energy ;
- hydroelectric energy ;
- thermal energy ;
- nuclear energy.

Article 8

Under the terms of this law, substances or combinations of substances manufactured or in the natural state are referred to which, because of their toxic, radioactive, corrosive or harmful nature, constitute a danger to human health, soil conservation. and subsoil, water, fauna and flora, the environment in general, when they are used or evacuated in the natural environment.

Article 9

This law covers the use of aggressive advertising techniques.

No one may advertise on a building without the authorization of the owner or the competent authorities under the conditions set by decree.

TITLE II THE ENVIRONMENT

CHAPTER 1: THE NATURAL ENVIRONMENT

Section 1: Soil and subsoil

Article 10

Soil and subsoil are natural resources to be preserved from all forms of degradation and of which it is important to promote sustainable use.

The use of the soil and the subsoil must be done while respecting the collective interests attached to their preservation.

As such, the right to property must be exercised without harming the general interest. The soil statutes must establish the rights and obligations of the holder vis-à-vis soil protection.

Article 11

Soils must be assigned to uses consistent with their vocation. The use of space for non-reversible uses must be limited and as rational as possible.

Article 12

Any development and land use project for agricultural, industrial or urban purposes, any research or exploitation project for raw materials from the subsoil are subject to prior authorization under the conditions set by decree.

Section II: Water resources and maritime waters

Article 13

The sampling points for water intended for human consumption must be surrounded by a protection perimeter provided for in article 51 of this code.

Any activity likely to adversely affect the quality of the water is prohibited or may be regulated within the protection perimeters.

Article 14

Water management can be granted.

The concessionaire is responsible for the quality of the water distributed in accordance with the standards in force.

Article 15

The occupants of a watershed can form an association for the protection of the environment.

Section III: Biological diversity

Article 16

The introduction, import and export of any animal or plant species are subject to prior authorization under the conditions set by decree.

Article 17

Apart from traditional hunting or the cases provided for in Articles 99 and 103 of the Penal Code relating to self-defense and the state of necessity, all forms of hunting are subject to obtaining a hunting license.

Article 18

All forms of fishing are the responsibility of the Competent National Authority:

- small-scale fishing must be carried out in compliance with regulations, taking into account good environmental management.
- industrial fishing requires for its exercise, the obtaining of a license issued by the competent administrative authority.

Article 19

The sale, exchange and marketing of game meat are regulated.

Section IV: Air

Article 20

Buildings, classified installations, vehicles and motor vehicles, industrial, commercial, craft or agricultural activities, owned or carried out by any natural or legal person must be designed and operated in accordance with the technical standards in force in terms of preservation of the environment. 'atmosphere.

CHAPTER II THE HUMAN ENVIRONMENT

Article 21

Land use plans, master plans, town plans and other town planning documents must take into account the imperatives of environmental protection in the choice, location and construction of activity zones economic, industrial, residential and leisure.

Article 22

The competent authority, under the terms of the regulations in force, may refuse the building permit if the constructions are spinning out to undermine the character or integrity of the neighboring places.

Article 23

No public or private work in the perimeter to which a plan applies can be carried out only if it is compatible with the latter, and if it takes into account the environmental provisions provided for by the texts in force.

Article 24

Construction work for public works such as roads and dams may be subject to an environmental impact study.

Article 25

The characteristics of the wastewater discharged must enable the receiving environments to meet the objectives assigned to them. The discharge of waste water into the public sewerage network must not harm the conservation of the works or the management of these networks.

Article 26

All wastes, including hospital and hazardous wastes, must be collected, treated and disposed of in an environmentally sound manner in order to prevent, eliminate or reduce their harmful effects on human health, natural resources, wildlife and wildlife flora and the quality of the environment.

Article 27

The burying of non-toxic waste in the ground and subsoil can only be carried out after authorization and subject to compliance with the technical prescriptions and special rules defined by decree.

Article 28

Waste disposal must comply with current standards and be designed in such a way as to facilitate their recovery.

To this end, the structures concerned are obliged to:

- develop and disseminate knowledge of appropriate techniques;
- conclude contracts organizing the reuse of waste;
- regulate manufacturing methods.

Article 29

All vehicles must be fitted with an audible warning device conforming to a type approved by the competent services and must not emit any noise likely to cause discomfort to road users and residents.

Article 30

In built-up areas, the use of audible warning devices is only authorized when absolutely necessary to give the necessary warnings to other road users.

At night, audible signals should only be used when absolutely necessary.

Article 31

When the urgency justifies it, the competent authority may take all appropriate measures to immediately stop any emission of noise liable to harm the health of living beings,

to constitute an excessive and unbearable annoyance for the neighborhood or to damage property.

Article 32

Early fires or fires lit with a view to renewing pastures, clearing cultivated land or within the framework of the development of pastoral, forest or savannah areas, national parks and wildlife reserves are subject to regulations by from the competent administrative authority.

TITLE III: GENERAL PRINCIPLES

Article 33

Everyone has the fundamental right to live in a healthy and balanced environment. It also has the duty to contribute individually or collectively to the safeguard of the natural heritage.

To this end, when a court rules on an application, it takes into account, in particular, the state of scientific knowledge, the solutions adopted by other countries and the provisions of international instruments.

Article 34

The national environmental protection policy is the responsibility of the State.

The State can develop environmental action plans with local communities or any other structure.

Article 35

When planning and carrying out acts that may have a significant impact on the environment, public authorities and individuals comply with the following principles:

35.1 - Precautionary principle

When planning or carrying out any action, preliminary measures are taken to avoid or reduce any risk or danger to the environment.

Anyone whose activities are likely to have an impact on the environment must, before acting, take into consideration the interests of third parties as well as the need to protect the environment.

If, in the light of experience or scientific knowledge, an action is considered likely to cause a risk or danger to the environment, this action should only be taken after a prior assessment indicating that it will not be has a detrimental impact on the environment.

35.2 Substitution

If an action likely to have a detrimental impact on the environment can be substituted by another action which presents a lower risk or danger, this last action is chosen even if it entails higher costs in relation to the values to be considered protect.

35.3 - Preservation of biological diversity

Any action must avoid having a significant detrimental effect on biological diversity.

35.4 Non-degradation of natural resources

In order to achieve sustainable development, it is necessary to avoid damaging natural resources such as water, air and soil which, in any event, are an integral part of the development process and should not not be taken into consideration in isolation. Irreversible effects on land should be avoided as far as possible.

35.5 - "Polluter-Pays" principle

Any natural or legal person whose actions and / or activities cause or are likely to cause damage to the environment is subject to a tax and / or a fee. It also assumes all remedial measures.

35.6 - Information.

Everyone has the right to be informed of the state of the environment and to participate in the procedures prior to making decisions that may have harmful effects on the environment.

3 5.7 Cooperation

Public authorities, international institutions, defense associations and individuals work together to protect the environment at all possible levels.

TITLE IV: THE OBLIGATIONS OF THE STATE AND LOCAL AUTHORITIES

CHAPTER I: GENERAL PROVISIONS

Article 36

The State owns the deposits and natural accumulations of hydrocarbons existing in Côte d'Ivoire including on the continental shelf.

Article 37

Rivers, lagoons, natural lakes, groundwater, springs, watersheds and maritime areas are in the public domain.

Article 38

Buildings, agricultural, industrial, commercial or craft establishments, vehicles or other movable objects owned, operated or owned by any natural or legal person, private or public must be built, operated or used in such a way as to meet the technical standards in force or enacted. in application of this law.

Article 39

Any major project likely to have an impact on the environment must be subject to a prior impact study. The same is true of programs, plans and policies that may affect the environment. A decree will specify the complete list.

All projects are subject to control and monitoring to verify the relevance of forecasts and adopt the necessary corrective measures.

Article 40

The Environmental Impact Assessment (EIA) includes at least:

- a description of the proposed activity;
- a description of the environment likely to be affected including the specific information necessary to identify or assess the effects of the proposed activity on the environment;
- a list of products used, if applicable;
- a description of the alternative solutions, if applicable;

- an assessment of the probable or potential effects of the proposed activity and of other possible solutions on the environment, including direct, indirect, cumulative effects in the short, medium and long terms;
- the identification and description of measures aimed at mitigating the effects of the proposed activity and other possible solutions on the environment, and an evaluation of these measures;
- an indication of knowledge gaps and uncertainties encountered in developing the necessary information;
- an indication of the risks to the environment of a neighboring State due to the proposed activity or to other possible solutions;
- a brief summary of the information provided under the previous headings;
- the definition of the methods of regular control and monitoring of environmental indicators before (initial state) ;
- during construction, during operation of the structure or development and, where applicable, after the end of operation (rehabilitation or redevelopment of the site);
- a financial estimate of the measures recommended to prevent, reduce or compensate for the negative effects of the project on the environment and regular monitoring and control measures of relevant environmental indicators.

Article 41

The examination of environmental impact studies by the Environmental Impact Study Office will result in the payment of a tax to the National Environment Fund, the basis of which will be specified by decree.

Article 42

On the proposal of the Competent National Authority, the Council of Ministers establishes and revises by decree the list of works, activities, planning documents for which the public authorities may not, under penalty of nullity, take any decision, approval or authorization without having an environmental impact study enabling them to assess the direct or indirect consequences for the environment.

Article 43

Installations which present the dangers or drawbacks referred to in Article 6 of this code are subject to authorization.

They cannot be opened without a prior authorization issued under the conditions set by decree at the request of the operator.

Installations are subject to declaration which, although they do not present such dangers or drawbacks, must nevertheless comply with the general prescriptions issued by the competent authority in order to ensure the protection of the interests referred to in article 6. The installations subject to authorization, which cause major risks (fires, explosions, toxic fumes, etc.) are subject to specific regulations aimed in particular at controlling urbanization in their immediate environment.

Article 44

Industrial fishing, hunting and capture are subject to a permit or license.

Article 45

The inspection of classified installations is carried out by sworn agents having the quality of Judicial Police Officer in the exercise of their function.

Article 46

The classified installations referred to in Article 6 be subject to a control and inspection tax, paid to the National Environment Fund.

Article 47

State installations assigned to national defense are subject to special rules.

Article 48

All existing classified installations benefiting from a period of 2 years from the promulgation of this law to be brought into conformity with its provisions and its implementing texts.

Article 49

Appropriate standards for the protection of the environment are established.

A label has been created for the most environmentally friendly consumer products.

Standards are also required for imported products.

Article 50

Companies or structures that are sources of significant pollution will be subject to an ecological audit by approved experts, at the expense of their promoters. The conditions of this audit will be specified by decree. The results of the ecological audit are transmitted to the Competent National Authority.

Article 51

Protection perimeters are established for the conservation or restoration of:

- ecosystems ;
- forests, afforestation, species and protected areas ;
- monuments, sites and landscapes ;
- hydraulic systems and water quality ;
- coastal areas ...

Article 52

The Competent National Authority may within the perimeters referred to in Article 49

- prohibit, limit or regulate activities incompatible with the objectives assigned to the area;
- implement programs to restore the natural environment or monuments;
- approve any development or action plan defining the means to achieve the objectives assigned to the area.

Article 53

The protection, conservation and enhancement of cultural and architectural heritage are an integral part of the national policy for the protection and enhancement of the environment.

Article 54

A list of protected sites and monuments has been drawn up which specifies the measures to be taken for the protection of the architectural, historical and cultural heritage throughout the national territory.

This list is revised every five years.

CHAPTER II: SPECIAL PROVISIONS

Section I: State obligations

Article 55

The State undertakes to:

- make the environment and its protection a global and integrated policy;
- take all appropriate measures to ensure or ensure compliance with the obligations arising from international conventions and agreements to which it is a party;
- prohibit any activity carried out under its control or within the limits of its jurisdiction, likely to lead to environmental degradation in another State or in regions not under any national jurisdiction;
- work in full cooperation with other States to take measures against transboundary pollution.

Article 56

The State determines the national environmental policy and sees to its implementation.

It ensures, by appropriate measures, the protection, conservation and management of the environment. However, the occupants of a watershed and / or the water users can form an association for the protection of the environment.

It regulates the establishment of access to dikes and sewer discharges in receiving environments.

It prohibits and regulates the exercise of activities likely to constitute, in one way or another, a threat to the environment, the integrity and the functioning of ecosystems.

Article 57

The State determines:

- the creation of a network of biological reserves in proportion to the land use;
- erosion control measures;
- measures to combat soil pollution by chemical substances, fertilizers, phytosanitary products and others whose use is permitted;
- measures to prevent diffuse pollution affect the soil and concrete measures to restore damaged soils;

- the protection perimeters of the water withdrawal points intended for human consumption;
- critical thresholds for atmospheric pollutants ;
- spaces allocated to industrial zones.

Article 58

- The state draws up a list animal and plant species that must be partially or fully protected because of their role in ecosystems, their aesthetic value, their rarity, the threat to their populations and finally the tourist, cultural and economic interest , and scientific that they represent;
- protected sites and monuments, specifying the measures to be taken for the protection of the national architectural, historical and cultural heritage;
- establishments, buildings and monuments which, although not classified or inscribed on which posting is prohibited.

This list is reviewed and corrected every five years.

Article 59

The State manages water while preserving the quality of its sources, avoiding wastage and increasing availability.

Article 60-

The State establishes standards designed to facilitate the recovery of waste. To this end, the structures concerned are obliged :

- develop and disseminate knowledge of appropriate techniques
- to conclude contracts organizing the reuse of waste;
- to regulate the methods of manufacture and use of certain materials or products, in order to facilitate the recovery of the elements of their composition.

Article 61

The State undertakes to:

- promote the use of renewable energies or not;
- fight against any form of waste of energy;
- fight against the waste of all energy sources, in particular wood resources.

Article 62

Any draft text relating to the environment is subject to the opinion and observation of the AuthorityCompetent National.

The National Competent Authority has a period of two months from the transmission of the project to follow up. The silence of the said authority is worth, at the end of the period, approval. Any divergence between the author of a project and the Competent National Authority is decided by the Council of Ministers.

Article 63

The State takes adequate measures to introduce environmental education, training and awareness into educational programs at all levels. It can approve associations for the defense of the environment and allocate subsidies to them.

Article 64

In its national environmental management policy, the State of Côte d'Ivoire integrates international cooperation.

Article 65

The Competent National Authority coordinates the national implementation mechanisms for monitoring international conventions and agreements relating to the environment.

Section II: The obligations of Local Authorities

Article 66

The municipalities are responsible for the collection, transport and disposal of household waste. This action can be undertaken in conjunction with the departments and regions or with private or public groups empowered for this purpose.

They are required to develop household waste collection and treatment plans with the assistance of the technical services of the competent structures.

They also ensure the elimination of other wastes which they can, having regard to their characteristics and the quantities produced, control or treat.

Article 67

Local authorities are required to have:

- an environmental management plan;
- one or more controlled landfills of household waste. They ensure that all illegal deposits are stopped.

They institute a health tax.

Section III: Obligations common to the State and Local Authorities

Article 68

It is the responsibility of the State, local communities and concessionaires to ensure, in compliance with environmental requirements, the rational exploitation of the natural hydrocarbon deposits and accumulations existing in Côte d'Ivoire, including on the continental shelf.

Article 69

The state and communities must ensure the ci cation, maintenance and upkeep of green spaces.

Article 70

Wastewater management is the responsibility of the State, local communities and all other structures likely to produce effluents liable to harm the environment.

It may be the subject of a concession.

Article 71

The state, regions, departments and local communities undertake to develop action programs and organize emergency plans in all areas with a view to protecting the environment.

Article 72

Environmental education, training and awareness are the responsibility of the State, local communities and defense associations.

Article 73

Public or private establishments and institutions responsible for teaching, research and information are required within the framework of their respective competences:

- to raise awareness of environmental problems through appropriate programs ;
- integrate programs into their activities to ensure better knowledge of the environment.

Section IV: Institutions

Article 74

For the application of this law, it is created :

- a Network of Biological Reserves in proportion to the intensification of land use ;
- an Air Quality Observatory;
- a National Environment Agency (ANDE), a public establishment of a particular category endowed with legal personality and financial autonomy ;
- a National Environment Fund (FNDE) ;
- a Waste Exchange

Furthermore, the summary judge is competent to ascertain or immediately stop any pollution or any form of environmental degradation.

The emergency procedure provided for in articles 221 to 230 of the Code of Civil, Commercial and Administrative Procedure is applicable.

TITLE V: PREVENTIVE AND PENAL PROVISIONS CHAPTER 1: PREVENTIVE PROVISIONS

Article 75

Are forbidden :

spills, discharges of all solid bodies, of all liquid and gaseous substances, in watercourses and bodies of water and their surroundings;

any activity likely to adversely affect the quality of air and water, both surface and underground.

Article 76

It is forbidden to discharge into maritime and lagoon areas any substances liable to:

- destroy sites and monuments of scientific, cultural, tourist or historical interest;
- destroy the flora and fauna ;
- constitute a danger to the health of living beings;
- undermine the aesthetic and tourist value of the lagoon, the sea and the coast.

Article 77

It is forbidden to discharge into maritime and lagoon waters wastewater, unless it has been previously treated in accordance with the standards in force;

- waste of all kinds not previously treated and harmful.

Article 78

It is forbidden to hold or abandon waste liable to:

- promote the development of disease vectors ;
- cause damage to people and property.

Article 79

Are forbidden :

- all discharges, runoffs, discharges or deposits of any kind likely to cause or increase pollution of continental, lagoon and maritime waters within the territorial limits;
- any illegal, degrading and / or unregulated exploitation;
- any emission into the atmosphere of toxic gases, smoke, soot, dust or any other chemical substance that does not comply with the regulations in force.

Article 80

In accordance with the special provisions of international conventions ratified by Côte d'Ivoire, dumping, dumping and incineration in maritime waters under Ivorian jurisdiction of substances of all kinds liable to:

- harm public health and biological maritime resources ;
- harm maritime activities including navigation and fishing ;
- alter the quality of maritime waters ;
- to degrade the values of amenities and the tourist potential of the sea and the coast

Article 81

Are forbidden :

- the unauthorized importation of waste into the national territory;
- waste deposits in the unauthorized public domain, including the maritime public domain as defined by the texts in force;
- dumping, incineration or elimination by any process whatsoever of waste in continental, lagoon and maritime waters, under Ivorian jurisdiction.

Article 82

All acts relating to the purchase, sale, import, export and transit of the substances or combination of substances referred to in article 8 of this law are prohibited on the national territory.

Article 83

If they have not been approved and / or if they do not benefit from a provisional authorization for sale, import or export issued by the competent authorities, any import, export , holding with a view to sale or offering for sale, even for free distribution, of any of the fertilizing materials defined in article 1 of this law.

Article 84

The use of the buzzer is prohibited in built-up areas and in the vicinity of hospitals and schools unless absolutely necessary and in this case, it must be brief and moderate.

Likewise, the emission of noise, lights and odors likely to harm the health of living beings or to constitute an excessive and unbearable annoyance for the neighborhood or to damage property is prohibited.

Article 85

All posting is prohibited on:

- buildings classified as historical monuments or registered ;
- natural monuments and in classified, registered or protected sites;
- monuments, sites and constructions listed by the competent authorities, benefiting from special protection ;
- road signs.

Article 86

Are forbidden :

- use of explosives, drugs, chemicals or bait in waters likely to intoxicate or destroy fish;
- the use of drugs, chemicals or bait likely to destroy game and / or make it unfit for consumption;
- uncontrolled bush fires.

Article-87

It is forbidden to :

- kill, injure or capture animals belonging to protected species ;
- destroy or damage habitats, larvae, and young protected species;
- destroy or damage the protected plants, pick all or part of them;
- transport or offer for sale all or part of a protected animal or plant;
- proceed to the felling of trees in classified forests, protected areas and national parks.

CHAPTER II: PENAL PROVISIONS

ARTICLE 88

Any legal or natural person who fails to carry out an environmental impact study prescribed by the competent authority and prior to any project likely to have harmful effects on the environment, is liable to suspension of activity or closure of 'establishment without prejudice to measures to repair damage caused to the environment, to persons and to property.

The falsification of an environmental impact study and / or its non-conformity are punishable by the same penalties.

Article 89

Is punished by imprisonment of two months to two years and a fine of 5,000,000 francs, anyone who proceeds or causes the felling of trees or animals in classified forests, protected areas and national parks .

Accomplices are punished with the same penalties.

Article 90

Any destruction of a listed site or monument is punished by a fine of 10,000,000 francs to 100,000,000 francs and imprisonment for six months to two years or only one of these two penalties.

Article 91

Is punished by imprisonment of one to six months and a fine of 1,000,000 francs to 5,000,000 francs or. One of these two penalties only: any manager of an establishment hindering the performance of the duties of the officers responsible for inspecting classified installations.

In the event of a repeat offense, the establishment is temporarily closed.

Article 92

Is liable to a fine of 5,000,000 francs to 50,000,000 francs without prejudice to a temporary suspension of activities, or to the closure of the establishment, any establishment that does not comply with the provisions of this law within two years of its promulgation.

Article 93

Anyone who continues to operate a classified installation without complying with the formal notice to comply with the determined technical requirements is punished by imprisonment from one month to one year and a fine of 200,000 francs to 2,000,000. of francs.

Article 94

Anyone who continues to operate a listed facility that has been closed, suspended or banned is punished by two months to two years imprisonment and from 50,000,000 francs to 100,000,000 francs or only one of these two penalties. .

Article 95

Is punished with a fine of 1,000,000 francs to 2,500,000 francs and imprisonment of six months to two years or one of these two penalties only anyone who unlawfully engages in research or exploitation work hydrocarbons.

Article 96

Anyone who carries out prohibited discharges or, without authorization, discharges subject to prior authorization as defined in articles is liable to a fine of 100,000,000 francs to 500,000,000 francs 74 to 86 of this code under the conditions set by decree or does not comply with the conditions determined by its authorization.

Article 97

Is punished by a fine of 2,000,000 francs to 50,000,000 francs and imprisonment of two months to two years or one of these two penalties only, any person having polluted continental waters by spills, flows, discharges and deposits of substances of any kind likely to cause or increase the pollution of continental waters and / or maritime waters within territorial limits.

In the event of a repeat offense, the penalty is doubled. The culprit can be condemned to clean up the polluted places.

The Competent National Authority may, in the event of negligence, refusal or resistance, do so or have it done at the costs and expenses of the interested party.

Article 98

Is punished by a fine of 100,000,000 francs to 1,000,000,000 francs and imprisonment from one to five years or one of the two penalties only without prejudice to the administrative sanctions in force, anyone, notwithstanding the special provisions of international conventions , carries out dumping, immersion and incineration in maritime waters under Ivorian jurisdiction, of substances of all kinds liable to

- harm public health and biological maritime resources;
- harm maritime activities including navigation and fishing ;
- alter the quality of maritime waters;
- to degrade the pleasure values and the tourist potential of the sea and the coast. ' The maritime administration can board any vessel caught in the act of dumping contaminants, including oil at sea.

In the event of a repeat offense, the fine is doubled and the Administration reserves the right to seize the vessel.

Article 99

Is liable to imprisonment from 1 to 5 years and a fine of 5,000,000 francs to 100,000,000 francs anyone who:

- deposit of waste in the national maritime public domain;
- imports without authorization of waste on the national territory;
- immerses, incinerates or eliminates by any process whatsoever waste in continental, lagoon and / or maritime waters under Ivorian jurisdiction.

Article 100

Is punished by a fine of 1,000,000 francs to 30,000,000 francs and imprisonment from three to twenty-four months or one of these two penalties only, the promoter of any business proceeding from illegal deposits.

Authorization to carry out any waste collection activity on national territory may be suspended for a period of at least two years.

Article 101

Anyone who purchases, sells for importation, transit, storage, landfilling or dumping on the national territory of hazardous waste or who signs an agreement for the authorization of such activities, is punished by imprisonment of 10 to 20 years and a fine of 500,000,000 francs to 5,000,000,000 francs.

The court which pronounced the sentence may

- order the seizure of any means used in the commission of the offense;
- order the seizure and disposal of waste at the expense of the owner of said waste.

Article 102

Anyone who deposits, abandons, throws garbage, waste, materials, or pours domestic wastewater in a public or private place, unless the deposit takes place at a location designated for this, is punished with a fine of 1,000 francs to 10,000 francs effect by the Competent Authority.

Likewise, any person who pollutes a public or private domain with excreta is subject to these penalties and / or required to clean the premises.

Are punished with a fine of 1,000 francs to 10,000 francs or fined. cleaning the premises, those which will have polluted public or private property with human waste, unless these locations have been designated for this purpose by the competent authority.

Article 103

Is liable to a fine of 10,000 francs to 500,000 francs anyone who:

- makes use in towns and in the vicinity of Hospitals and schools, of audible warning devices except in cases of immediate danger;
- makes untimely use and without absolute necessity, outside the agglomerations of audible warning devices ;
- makes use, without absolute necessity of audible warning devices at night ;
- emits noises likely to cause annoyance to road users and residents;
- uses motor vehicles fitted with audible warning devices that do not conform to the type approved by the competent services ;
- emits noises, lights. or odors liable to harm the health of living beings, to constitute an excessive and unbearable annoyance for the neighborhood or to damage property.

Article 104

Is punished by a fine of 50,000 francs to 5,000,000 francs and imprisonment for a maximum of three months anyone who does:

- advertising on a building without the authorization of the owner and the competent authorities ;
- posters and graffiti on listed buildings or listed historical monuments, on natural monuments and in listed or protected sites.

Article 105

The mitigating circumstances and the suspension are not applicable to the offenses provided for by this code relating to hazardous waste.

Article 106

Attempting and complicity in the offenses provided for by this code are punishable by the same penalties as the offense itself.

Article 107

Infractions are recorded on report by sworn officers of the National Authority Competent.

Article 108

The administration responsible for the environment can compromise in any circumstance and at any time during the proceedings before any decision on the merits.

The transaction request is submitted to the Competent National Authority which fixes, in the event of acceptance, the amount thereof.

Article 109

The prosecution of offenses under this code obeys the rules defined by the code of criminal procedure.

Article 110

Local authorities, regularly declared environmental defense associations or any person must refer the matter to the Competent National Authority before any recourse to the courts and / or exercise the rights granted to the civil party with regard to the facts constituting an offense falling under this law and causing direct or indirect damage to collective or individual interests.

TITLE VI: FINAL PROVISIONS Article 111

The terms of application of the provisions of this law will be the subject of decrees.

Article 112

This law repeals all previous contrary provisions.

Article 113

This law will be published in the Official Gazette of the Republic of Côte d'Ivoire and executed as law of the State.

Done in Abidjan, October 3, 1996

Henri Konan BEDIE
