# Supplementary material for: Ivory Coast without ivory: Massive extinction of African forest elephants in Côte d’Ivoire
Source: PLoS One. 2020 Oct 14;15(10):e0232993. doi: 10.1371/journal.pone.0232993 (PMC7556483; doi:10.1371/journal.pone.0232993)
Supplement: S2 File — (PDF) [file pone.0232993.s005.pdf]

**REPUBLIQUE DE COTE D'IVOIRE**
**Union - Discipline - Travail**

Loi n° 96-766 du 3 octobre 1996
portant Code de l'Environnement

L'ASSEMBLEE NATIONALE, a adopté,

LE PRESIDENT DE LA REPUBLIQUE, promulgue la loi dont la teneur suit :

## TITRE 1: DEFINITIONS, OBJECTIFS ET DOMAINE D'APPLICATION

### CHAPITRE I : DEFINITIONS

**Article ler:**

Aux termes de la présente loi :

**L'environnement** est l'ensemble des éléments physiques, chimiques, biologiques et des facteurs socio-économiques, moraux et intellectuels susceptibles d'avoir un effet direct ou indirect, immédiat ou à terme sur le développement du milieu, des êtres vivants et des activités humaines.

**L'environnement humain** concerne le cadre de vie et l'aménagement du territoire.

**L'environnement naturel** comprend :

- le sol et le sous-sol,
- les ressources en eau,
- l'air,
- la diversité biologique,
- les paysages, sites et monuments...

**Les ressources en eau** comprennent les eaux intérieures de surface et les eaux souterraines.

**L'air** est la couche atmosphérique dont la modification physique, chimique ou autre peut porter atteinte à la santé des êtres vivants, aux écosystèmes et à l'environnement en général.

**Le paysage** est une portion du territoire dont les divers éléments forment un ensemble pittoresque par la disposition de ses composants ou les contours de ses formes ou l'effet de ses couleurs.

**Le site** est une portion de paysage particularisée par sa situation géographique et/ou son histoire.

**Le monument naturel** est un élément ou un groupe d'éléments dus à la nature tels que rochers, arbres, sources, bouleversements du sol, accidents géologiques ou autres qui, séparément ou ensemble, forment un panorama digne d'attention.

**L'écosystème** est un ensemble structuré qui englobe en une seule ou et même unité fonctionnelle le biotope et la biocénose.

**Le biotope** est l'aire géographique où l'ensemble des facteurs physiques et chimiques de l'environnement reste sensiblement constant.

**La biocénose** est l'ensemble des végétaux et animaux qui vivent dans les mêmes conditions de milieu et dans un espace donné de dimensions variables.

**L'écologie** est l'étude des milieux où vivent, se reproduisent et meurent les êtres vivants ainsi que des rapports de ces êtres avec le milieu et leur protection contre toute pollution.

**La diversité biologique** est la variabilité des organismes vivants de toute origine y compris, entre autres, les écosystèmes terrestres, marins et autres écosystèmes aquatiques et les complexes écologiques

dont il fait partie ; cela comprend la diversité au sein des espèces et entre espèces ainsi que celle des écosystèmes.

**La pollution** est la contamination ou la modification directe ou indirecte de l'environnement provoquée par, tout acte susceptible :

- d'altérer le milieu de vie de l'homme et des autres espèces vivantes
- de nuire à la santé, à la sécurité, au bien-être de l'homme, de la flore et de la faune ou aux biens collectifs et individuels.

**La pollution des eaux** est l'introduction dans le milieu aquatique de toute substance susceptible de modifier les caractéristiques physiques, chimiques et/ou biologiques de l'eau et de créer des risques pour la santé de l'homme, de nuire à la faune et à la flore terrestres et aquatiques, de porter atteinte à l'agrément des sites ou de gêner toute autre utilisation rationnelle des eaux.

**La pollution atmosphérique** ou pollution de l'air est l'émission volontaire ou accidentelle dans la couche atmosphérique de gaz, de fumée ou de substances de nature à créer des nuisances pour les êtres vivants, à compromettre leur santé ou la sécurité publique ou à nuire à la production agricole, à la conservation des édifices ou au caractère des sites et paysages.

**La pollution transfrontière** est la pollution qui a son origine dans un pays et dont les effets se propagent dans d'autres pays.

**Les aires protégées** sont les zones spécialement consacrées à la préservation de la diversité biologique et des ressources naturelles qui y sont associées.

**Les zones maritimes** comprennent : les eaux archipélagiques, la mer territoriale, la zone économique exclusive, le plateau continental ainsi que le rivage de la mer, les fonds marins et le sous-sol correspondant.

**L'établissement humain** comprend l'ensemble des agglomérations urbaines et rurales, des infrastructures et équipements dont elles doivent disposer pour assurer à leurs habitants un cadre de vie agréable et une existence saine, harmonieuse et équilibrée.

**Les hydrocarbures** sont des substances énergétiques, fluides (liquides ou gazeuses).

**La nuisance** est toute atteinte à la santé des êtres vivants, de leur fait ou non, par l'émission de bruits, de lumière, d'odeurs etc..

**Les déchets** sont des produits solides, liquides ou gazeux, résultant des activités des ménages, d'un processus de fabrication ou tout bien meuble ou immeuble abandonné ou qui menace ruine.

**Les déchets dangereux** sont des produits solide liquides ou gazeux, qui présentent une menace sérieuse ou des risques particuliers, pour la santé, la sécurité des êtres vivants et la qualité de l'environnement.

**Les matières fertilisantes** sont les engrais, les amendements et tout produit dont l'emploi, contribue à améliorer la productivité agricole.

**Les risques naturels** sont les catastrophes et calamités naturelles qui peuvent avoir des effets imprévisibles sur l'environnement et la santé.

**L'accident majeur** est défini comme un événement tel qu'une émission de substances dangereuses, un incendie, une explosion résultant d'un développement incontrôlé d'une activité industrielle, agricole ou domestique.

**Les plans d'urgence** se définissent comme l'organisation rapide et rationnelle, sous la responsabilité d'une autorité déterminée, des moyens de toute nature pour faire face à une situation d'une extrême gravité.

**Les feux de brousse** sont des feux allumés volontairement ou non, quelle qu'en soit l'ampleur, causant des dommages à 1'homme et à ses biens, à la flore et à la faune.

**La désertification** désigne la dégradation des terres dans les zones arides, semi-arides et subhumides sèches par suite de divers facteurs, parmi lesquels les variations climatiques et les activités humaines.

**La pêche** consiste en la capture, l'extraction ou la récolte de poissons, cétacés, chéloniens végétaux, planctons ou d'animaux vertébrés ou invertébrés vivant partiellement ou complètement dans le milieu aquatique.

**La chasse** consiste en tout acte tendant

- à blesser ou tuer pour s'approprier ou non de tout ou partie de sa dépouille, un animal en liberté dans son milieu naturel au sens des textes législatifs et réglementaires en vigueur ;

- détruire les oeufs des oiseaux et des reptiles.

**La capture** consiste en tout acte tendant à:

- priver de sa liberté, un animal sauvage ;

- récolter et retirer hors de leur lieu naturel d'éclosion, les oeufs des oiseaux ou des reptiles.

**L'étude d'impact environnemental** est un rapport d'évaluation de l'impact probable d'une activité envisagée sur l'environnement.

**Le Bureau d'Etudes d'Impact Environnemental** est un service à la disposition de l'Autorité Nationale Compétente chargé d'examiner les études d'impact.

**L'audit environnemental** est une procédure d'évaluation et de contrôle des actions de protection de l'environnement.

**L'Autorité Nationale Compétente** est une entité unique ou un groupement d'entités dont les compétences sont définies par décret.

**L'Association de défense l'environnement** est l'organisation par laquelle deux ou plusieurs personnes mettent en commun leurs connaissances ou leurs activités en vue de concourir à la défense de l'environnement.

## CHAPITRE II : OBJECTIFS

**Article 2**

Le présent code vise à :

- protéger les sols, sous-sols, sites, paysages et monuments nationaux, les formations végétales, la faune et la flore et particulièrement les domaines classés, les parcs nationaux et réserves existantes ;

- établir les principes fondamentaux destinés à gérer, à protéger l'environnement contre toutes les formes de dégradation afin de valoriser les ressources naturelles, de lutter contre toutes sortes de pollution et nuisances ;

- améliorer les conditions de vie des différents types de population dans le respect de l'équilibre avec le milieu ambiant;

- créer les conditions d'une utilisation rationnelle et durable des ressources naturelles pour les générations présentes et futures ;

- garantir à tous les citoyens, un cadre de vie écologiquement sain et équilibré ;

- veiller à la restauration des milieux endommagés.

## CHAPITRE III: DOMAINE D'APPLICATION

**Article 3**

La présente loi ne fait pas obstacle à l'application des dispositions législatives et réglementaires concernant l'urbanisme et les constructions, la santé, l'hygiène, la sécurité et la tranquillité publique, la protection des écosystèmes et d'une manière générale à l'exercice des pouvoirs de police.

**Article 4**

La présente loi ne s'applique pas aux activités militaires et aux situations de guerre. Toutefois, les auteurs de telles activités sont tenus de prendre en compte les préoccupations de protection de l'environnement.

**Article 5**

La présente loi s'applique à toutes les formes de pollution telles que définies à l'article ler du présent code et susceptibles de provoquer une altération de la composition et de la consistance de la couche atmosphérique avec des conséquences dommageables pour la santé des êtres vivants, la production, les biens et l'équilibre des écosystèmes.

**Article 6**

Sont soumis aux dispositions de la présente loi ;

- les installations classées telles que définies dans leur nomenclature : les usines, dépôts, mines, chantiers, carrières, stockages souterrains ou en surface, magasins et ateliers ;

- les installations exploitées ou détenues par toute personne physique ou morale, publique ou privée qui peuvent présenter des dangers ou des inconvénients, soit pour la commodité, soit pour la santé, la sécurité et la salubrité publique ;

- les déversements, écoulements, rejets et dépôts' susceptibles de provoquer ou d'accroître la dégradation du milieu récepteurs.

**Article 7**

Sont visés, aux termes de la présente loi, les différents types d'énergie suivants

- l'énergie solaire
- l'énergie de biomasse
- l'énergie éolienne ;
- l'énergie géothermique
- l'énergie hydro-électrique
- l’énergie thermique
- l’énergie nucléaire.

**Article 8**

Aux termes de la présente loi, sont visées les substances ou combinaisons de substances fabriquées ou à l'état naturel susceptibles, en raison de leur caractère toxique, radioactif, corrosif ou nocif de constituer un danger pour la santé des personnes, la conservation des sols et sous-sol, des eaux, de la faune et de la flore, de l'environnement en général, lorsqu'elles sont utilisées ou évacuées dans le milieu naturel.

**Article 9**

Est visée par la présente loi, l'utilisation de techniques publicitaires agressives.

Nul ne peut faire de la publicité sur un immeuble sans l'autorisation du propriétaire ou des autorités compétentes dans les conditions fixées par décret.

## TITRE II L'ENVIRONNEMENT

### CHAPITRE 1 : L'ENVIRONNEMENT NATUREL

#### Section 1 : Le sol et le sous-sol

**Article 10**

Le sol et le sous-sol constituent des ressources naturelles à préserver de toutes formes de dégradation et dont il importe de promouvoir l'utilisation durable.

L'usage du sol et du sous-sol doit être fait en respectant les intérêts collectifs attachés à leur préservation.

A ce titre, le droit de propriété doit être exercé sans qu'il nuise à l'intérêt général. Les statuts du sol doivent établir les droits et les obligations du titulaire vis-à-vis d'une protection du sol.

**Article 11**

Les sols doivent être affectés à des usages conformes à leur vocation. L'utilisation d'espace pour des usages non réversibles doit être limitée et la plus rationnelle possible.

**Article 12**

Tout projet d'aménagement et d'affectation du sol à des fins agricoles, industrielles ou urbaines, tout projet de recherche ou d'exploitation des matières premières du sous-sol sont soumis à autorisation préalable dans les conditions fixées par décret.

#### Section II : Les ressources en eau et les eaux maritimes

**Article 13**

Les points de prélèvement de l'eau destinée à la consommation humaine, doivent être entourés d'un périmètre de protection prévu à l'article 51 du présent code.

Toute activité susceptible de nuire à la qualité des eaux est interdite ou peut être réglementée à l'intérieur des périmètres de protection.

**Article 14**

La gestion de l'eau peut-être concédée.

Le concessionnaire est responsable de la qualité de l'eau distribuée conformément aux normes en vigueur.

**Article 15**

Les occupants d'un bassin versant peuvent se constituer en association pour la protection du milieu.

**Section III** : **La diversité biologique**

**Article 16**

L'introduction, l'importation et l'exportation de toute espèce animale ou végétale sont soumises à autorisation préalable dans les conditions fixés par décret.

**Article 17**

En dehors de la chasse traditionnelle ou des cas prévus par les articles 99 et 103 du Code Pénal relatifs à la légitime défense et à l'état de nécessité, toutes formes de chasse sont soumises à l'obtention d'un permis de chasse.

**Article 18**

Toutes les formes de pêche relèvent de l'Autorité Nationale Compétente :

- la pêche artisanale doit être exercée dans le respect de la réglementation en tenant compte d'une bonne gestion de l'environnement

- la pêche industrielle requiert pour son exercice, l'obtention d'une licence délivrée par l'autorité administrative compétente.

**Article 19**

La vente, l'échange, la commercialisation de la viande de chasse sont réglementés.

**Section IV: L'Air**

**Article 20**

Les immeubles, les installations classées, les véhicules et engins à moteur, les activités industrielles, commerciales, artisanales ou agricoles, détenus ou exercées par toute personne physique ou morale doivent être conçus et exploités conformément aux normes techniques en vigueur en matière de préservation de l'atmosphère.

**CHAPITRE II L'ENVIRONNEMENT HUMAIN**

**Article 21**

Les plans d'aménagement du territoire, les schémas directeurs, les plans d'urbanisme et autres documents d'urbanisme doivent prendre en compte les impératifs de protection de l'environnement dans le choix, l'emplacement et la réalisation des zones d'activités économique, industrielle, de résidence et de loisirs.

**Article 22**

L'autorité compétente, aux termes des règlements en vigueur, peut refuser le permis de construire si les constructions sont de filature à porter atteinte au caractère ou à l'intégrité des lieux avoisinants.

**Article 23**

Aucun travail public ou privé dans le périmètre auquel s'applique un plan ne peut être réalisé que s'il est compatible avec ce dernier, et s'il prend en considération les dispositions d'ordre environnemental, prévues par les textes en vigueur.

**Article 24**

Les travaux de construction d'ouvrages publics tels que routes, barrages, peuvent être soumis à une étude d'impact environnemental.

**Article 25**

Les caractéristiques des eaux résiduaires rejetées doivent permettre aux milieux récepteurs de satisfaire aux objectifs qui leur sont assignés. Le déversement des eaux résiduaires dans le réseau d'assainissement public ne doit nuire ni à la conservation des ouvrages ni à la gestion de ces réseaux.

**Article 26**

Tous les déchets, notamment les déchets hospitaliers et dangereux, doivent être collectés, traités et éliminés de manière écologiquement rationnelle afin de prévenir, supprimer ou réduire leurs effets nocifs sur la santé de l'homme, sur les ressources naturelles, sur la faune et la flore et sur la qualité de l'Environnement.

**Article 27**

L'enfouissement dans le sol et le sous-sol de déchets non toxiques ne peut être opéré qu'après autorisation et sous réserve du respect des prescriptions techniques et règles particulières définies par décret.

**Article 28**

L'élimination des déchets doit respecter les normes en vigueur et être conçue de manière à faciliter leur valorisation.

A cette fin,, il est fait obligation aux structures concernées de :

- développer et divulguer la connaissance des techniques appropriées ;
- conclure des contrats organisant la réutilisation des déchets ;
- réglementer les modes de fabrication.

**Article 29**

Tous les engins doivent être munis d'un avertisseur sonore conforme à un type homologué par les services compétents et ne doivent pas émettre de bruit susceptible de causer une gêne aux usagers de la route et aux riverains.

**Article 30**

En agglomération, l'usage des avertisseurs sonores n’est autorisé qu'en cas de besoin absolu pour donner les avertissements nécessaires aux autres usagers de la route.

La nuit, les signaux sonores ne doivent être utilisés qu'en cas de nécessité absolue.

**Article 31**

Lorsque l'urgence le justifie l'autorité compétente peut prendre toutes mesures appropriées pour faire cesser immédiatement toute émission de bruits susceptibles de nuire à la santé des êtres vivants, de constituer une gêne excessive et insupportable pour le voisinage ou d'endommager les biens.

**Article 32**

Les feux précoces ou les feux allumés en vue du renouvellement des pâturages, de débroussaillement des terrains de culture ou dans le cadre de l'aménagement des zones pastorales, forestières ou savanicoles, des parcs nationaux et des réserves fauniques font l'objet de réglementation de la part de l'autorité administrative compétente.

## **TITRE III : PRINCIPES GENERAUX**

### **Article 33**

Toute personne a le droit fondamental de vivre dans un environnement sain et équilibré. Il a aussi le devoir de contribuer individuellement ou collectivement à la sauvegarde du patrimoine naturel.

A cette fin, lorsqu'un tribunal statue sur une demande, il prend notamment en considération, l'état des connaissances scientifiques, les solutions adoptées par les autres pays et les dispositions des instruments internationaux.

### **Article 34**

La politique nationale de protection de l'environnement incombe à l'Etat.

L'Etat peut élaborer des plans d'actions environnementales avec les collectivités locales ou toute autre structure.

### **Article 35**

Lors de la planification et de l'exécution d'actes pouvant avoir un impact important sur l'environnement, les autorités publiques et les particuliers se conforment aux principes suivants :

#### **35.1 - Principe de précaution**

Lors de la planification ou de l'exécution de toute action, des mesures préliminaires sont prises de manière à éviter ou réduire tout risque ou tout danger pour l'environnement.

Toute personne dont les activités sont susceptibles d'avoir un impact sur l'environnement doit, avant d'agir, prendre en considération les intérêts des tiers ainsi que la nécessité de protéger l'environnement.

Si, à la lumière de l'expérience ou des connaissances scientifiques, une action est jugée susceptible de causer un risque ou un danger pour l'environnement, cette action n'est entreprise qu'après une évaluation préalable indiquant qu'elle n'aura as d'impact préjudiciable à l'environnement.

#### **35.2 Substitution**

Si à une action susceptible d'avoir un impact préjudiciable à l'environnement, peut être substituée une autre action qui présente un risque ou un danger moindre, cette dernière action est choisie même si elle entraîne des coûts plus élevés en rapport avec les valeurs à protéger.

**35.3 - <u>Préservation de la diversité biologique</u>**

Toute action doit éviter d'avoir un effet préjudiciable notable sur la diversité biologique.

**35.4 <u>Non-dégradation des ressources naturelles</u>**

Pour réaliser un développement durable, il y a lieu d'éviter de porter atteinte aux ressources naturelles tels que l'eau, l'air et les sols qui,' en tout état de cause, font partie intégrante du processus de développement et ne doivent pas être prises en considération isolement. Les effets irréversibles sur les terres doivent être évités dans toute la mesure du possible.

**35.5 - <u>Principe "Pollueur-Payeur"</u>**

Toute personne physique ou morale dont les agissements et/ou les activités causent ou sont susceptibles de causer des dommages à l'environnement est soumise une taxe et/ou à une redevance. Elle assume en outre toutes les mesures de remise en état.

**35.6 - <u>Information</u>.**

Toute personne a le droit d'être informée de l'état de l'environnement et de participer aux procédures préalables à la prise de décisions susceptibles d'avoir des effets préjudiciables à l’environnement.

**3 5.7 <u>Coopération</u>**

Les autorités publiques, les institutions internationales, les associations de défense et les particuliers concourent à protéger l'environnement à tous les niveaux possibles.

## <u>TITRE IV</u>: <u>LES OBLIGATIONS DE L'ETAT ET DES COLLECTIVITES LOCALES</u>

### <u>CHAPITRE I</u> : <u>DISPOSITIONS GENERALES</u>

**<u>Article 36</u>**

L'Etat est propriétaire des gisements et des accumulations naturelles d'hydrocarbures existant en Côte d'Ivoire y compris sur le plateau continental.

**<u>Article 37</u>**

Les cours d'eau, les lagunes, les lacs naturels, les nappes phréatiques, les sources, les bassins versants et les zones maritimes sont du domaine public.

**<u>Article 38</u>**

Les immeubles, établissements agricoles, industriels, commerciaux ou artisanaux, véhicules ou autres objets mobiliers possédés, exploités ou détenus par toute personne physique ou morale, privée ou publique devront être construits, exploités ou utilisés de manière à satisfaire aux normes techniques en vigueur ou édictées en application de la présente loi.

**<u>Article 39</u>**

Tout projet important susceptible d'avoir un impact sur l'environnement doit faire l'objet d'une étude d'impact préalable. Il en est de même des programmes, plans et politiques pouvant affecter l'environnement. Un décret en précisera la liste complète..

Tout projet fait l'objet d'un contrôle et d'un suivi pour vérifier la pertinence des prévisions et adopter les mesures correctives nécessaires.

**Article 40**

L'Etude d'Impact Environnemental (EIE) comporte au minimum :

- une description de l'activité proposée ;

- une description de l'environnement susceptible d'être affecté y compris les renseignements spécifiques nécessaires pour identifier ou évaluer les effets de l'activité proposée sur l'environnement ;

- une liste des produits utilisés le cas échéant ;

- une description des solutions alternatives, le cas échéant ;

- une évaluation des effets probables ou potentiels de l'activité proposée et des autres solutions possibles sur l'environnement, y compris les effets directs, indirects, cumulatifs à court , à moyen et long termes ;

- l'identification et la description des mesures visait, atténuer les effets de l'activité proposée et les autres solutions possibles, sur l'environnement**,** et une évaluation de ces mesures **;**

**-** une indication des lacunes en matière de connaissance et des incertitudes rencontrées dans la mise au point de l'information nécessaire ;

- une indication sur les risques pour l'environnement d'un Etat voisin dus à l'activité proposée ou aux autres solutions possibles ;

- un bref résumé de l'information fournie au titre des rubriques précédentes ;

- la définition des modalités de contrôle et de suivi réguliers d'indicateurs environnementaux avant (état initial),. pendant le chantier, durant l'exploitation de l'ouvrage ou de l'aménagement et le cas échéant, après la fin de l'exploitation (remise en état ou réaménagement des lieux) ;

- une estimation financière des mesures préconisées pour prévenir, réduire ou compenser les effets négatifs du projet sur l'environnement et des mesures de suivi et contrôle réguliers d'indicateur environnementaux pertinents.

**Article 41**

L'examen des études d'impact environnemental par le Bureau d'Etude d'Impact Environnemental, donnera lieu au versement d'une taxe au Fonds National de l'Environnement dont l'assiette sera précisée par décret.

**Article 42**

Sur proposition de l'Autorité Nationale Compétente, le Conseil des Ministres établit et révise par décret la liste des travaux, activités, documents de planification pour lesquels les autorités publiques ne pourront, sous peine de nullité, prendre aucune décision, approbation ou autorisation sans disposer d'une étude d'impact environnemental leur permettant d'en apprécier les conséquences directes ou indirectes pour l'environnement.

**Article 43**

Sont soumises à autorisation, les installations qui présentent des dangers ou inconvénients visés à l'article 6 du présent code.

Elles ne peuvent être ouvertes sans une autorisation préalable délivrée dans les conditions fixées par décret sur demande de l'exploitant.

Sont soumises à déclaration, les installations qui, bien que ne présentant pas de tels dangers ou inconvénients, doivent néanmoins respecter les prescriptions générales édictées par l'autorité compétente en vue d'assurer la protection des intérêts visés à l'article 6. Les installations soumises à autorisation, qui occasionnent des risques majeurs (incendies, explosions, émanations toxiques, etc.) font l'objet d'une réglementation spécifique visant notamment à maîtriser 1'urbanisation dans leur environnement immédiat.

**Article 44**

Sont soumises à permis ou à licence, la pêche industrielle, la chasse et la capture.

**Article 45**

L'inspection des installations classées est assurée par des agents assermentés ayant la qualité d'Officier de Police Judiciaire dans l'exercice de leur fonction.

**Article 46**

Les installations classées visées à l'article 6 soit assujetties à une taxe de contrôle et d'inspection, versée au Fonds National de l'Environnement.

**Article 47**

Les installations de l'Etat affectées à la défense nationale, sont soumises à des règles particulières.

**Article 48**

Toutes les installations classées existantes bénéficiant d'un délai de 2 ans à compter de la promulgation de la présente loi pour être mise en conformité avec ses dispositions et ses textes d'application..

**Article 49**

Il est instauré des normes appropriées pour la protection de l'environnement.

Il est créé un label pour les produits de consommation les plus respectueux de l'environnement.

Des normes sont également exigées pour les produits importés.

**Article 50**

Les entreprises ou ouvrages, sources de pollutions importantes seront soumis à un audit écologique par des experts agréés, aux frais de leurs promoteurs. Les conditions de cet audit seront précisées par décret. Les résultats de l'audit écologique sont transmis à l'Autorité Nationale Compétente.

**Article 51**

Il est institué des périmètres de protection en vue de la conservation ou de la restauration des :

- écosystèmes,
- forêts, boisements, espèces et espaces protégés,
- monuments, sites et paysages,
- systèmes Hydrauliques et de la qualité des eaux,
- espaces littoraux...

**Article 52**

L'Autorité Nationale Compétente peut à l'intérieur des périmètres visés à l'article 49

- interdire, limiter ou réglementer les activités incompatibles avec les objectifs assignés à la zone ;

- mettre en œuvre des programmes de restauration du milieu naturel ou des monuments ;

- approuver tout plan d'aménagement ou d'action définissant les moyens d'atteindre les objectifs assignés à la zone .

**Article 53**

La protection, la conservation et la valorisation du patrimoine culturel et architectural font partie intégrante de la politique nationale de protection et de la mise en valeur de l'environnement.

**Article 54**

Il est dressé une liste de sites et monuments protégés qui précise les mesures à prendre pour la protection du patrimoine architectural, historique et culturel sur tout le territoire national.

Cette liste est révisée tous les cinq ans.

## CHAPITRE Il: DISPPOSITIONS PARTICULIERES

### Section I : Les obligations de l'Etat

**Article 55**

L'Etat s'engage à :

- faire de l'environnement et de sa protection une politique globale et intégrée ;

- prendre toutes dispositions appropriées pour assurer ou faire assurer le respect des obligations découlant des conventions et accords internationaux auxquels il est partie ;

- interdire toute activité menée sous son contrôle ou dans les limites de sa juridiction, susceptible d'entraîner une dégradation de l'environnement dans un autre Etat ou dans des régions ne relevant d'aucune juridiction nationale ;

- oeuvrer en toute coopération avec les autres Etats pour prendre les mesures contre la pollution transfrontière.

**Article 56**

L'Etat déterminé la politique nationale de l'Environnement et veille à sa mise en oeuvre.

Il assure, par des mesures idoines, la protection, la conservation et la gestion de l'environnement. Toutefois, les occupants d'un bassin versant et/ou les utilisateurs de l'eau peuvent se constituer en association pour la protection du milieu.

Il réglemente l'établissement d'accès aux digues et déversements d'égouts dans les milieux récepteurs.

Il interdit et réglemente l'exercice d'activités susceptibles de constituer, d'une manière ou d'une autre, une menace pour, l'environnement, l'intégrité et le fonctionnement des écosystèmes.

**Article 57**

L'Etat détermine :

- la création d'un réseau de réserves biologiques en proportion avec l'usage des sols ;

- les mesures de lutte contre l'érosion ;

- les mesures de lutte contre la pollution du sol par des substances chimiques, les engrais, les produits phytosanitaires et autres dont l'usage est admis ;

- les mesures de prévention des pollutions diffuses affectent le sol et les mesures concrètes de restauration des sols endommagés ;

- les périmètres de protection des points de prélèvement de l'eau destinée à la consommation humaine;

- les seuils critiques des polluants atmosphériques

- les espaces alloués aux zones industrielles

. **Article 58**

L'Etat dresse une liste

- des espèces animales et végétales qui doivent être partiellement ou intégralement protégées en raison de leur rôle dans les écosystèmes, de leur valeur esthétique, de leur rareté, de la menace qui pèse sur leurs populations et enfin de l'intérêt touristique, culturel, économique, et scientifique qu'elles représentent ;

- des sites et monuments protégés en précisant les mesures à prendre pour la protection du patrimoine architectural, historique et culturel national ;

- des établissements, édifices et monuments qui, bien que non classés ou inscrits sur lesquels l'affichage est interdit.

Cette liste est revue et corrigée tous les cinq ans.

**Article 59**

L'Etat assure la gestion de l'eau en préservant la qualité de ses sources, en évitant le gaspillage et en accroissant la disponibilité.

**Article 60**-

L'Etat établit des normes conçues de manière à faciliter la valorisation des déchets.

A cette fin, il est fait obligation aux structures concernées

- de développer et de divulguer la connaissance des techniques appropriées

- de conclure des contrats organisant la réutilisation des déchets ;

- de réglementer les modes de fabrication et d'utilisation de certains matériaux ou produits, afin de faciliter la récupération des éléments de leur composition.

**Article 61**

L'Etat s’engage à :

- promouvoir l’utilisation des énergies renouvelables ou non ;
- lutter contre toute forme de gaspillage des énergies ;
- lutter contre le gaspillage de toutes les sources d'énergie notamment les ressources ligneuses.

**Article 62**

Tout projet de texte relatif à l'environnement est soumis à l'avis et à l'observation de l'Autorité Nationale Compétente.

L'Autorité Nationale Compétente dispose d'un délai de deux mois à compter de la transmission du projet pour donner suite. Le silence de ladite autorité vaut, au terme du délai, approbation. Toute divergence entre l'auteur d'un projet et l'Autorité Nationale Compétente est tranchée par le Conseil des Ministres.

**Article 63**

L'Etat prend les mesures adéquates pour introduire l'éducation, la formation et la sensibilisation environnementales dans les programmes d'enseignement à tous les niveaux.. Il peut donner son agrément aux associations de défense de l'environnement et leur allouer des subventions.

**Article 64**

Dans sa politique nationale de gestion de l'environnement, l'Etat de Côte d'Ivoire intègre la coopération internationale.

**Article 65**

L'Autorité Nationale Compétente coordonne les mécanismes nationaux de mise en oeuvre de suivi des conventions et accords internationaux relatifs à l'environnement.

## Section II : Les obligations des Collectivités Locales

**Article 66**

Les communes sont responsables de la collecte, du transport et de l'élimination des déchets ménagers. Cette action peut être entreprise en 1iaison avec les départements et les régions ou avec des groupes privés ou publics habilités à cet effet.

Elles ont l'obligation d'élaborer des schémas de collecte et de traitement des déchets ménagers avec le concours des services techniques des structures compétentes.

Elles assurent également l'élimination d'autres déchets qu'elles peuvent, eu égard à leurs caractéristiques et aux quantités produites, contrôler ou traiter.

**Article 67**

Les collectivités locales sont tenues d'avoir :

- un plan de gestion de l'environnement ;

- une ou plusieurs décharges contrôlées d'ordures ménagères.

Elles veillent à enrayer tous les dépôts sauvages.

Elles instituent une taxe de salubrité.

## Section III : Les obligations communes à l'Etat et aux Collectivités Locales

**Article 68**

Il incombe à l'Etat, aux collectivités locales et aux concessionnaires d'assurer, dans le respect des prescriptions environnementales, l'exploitation rationnelle des gisements et accumulations naturelles d'hydrocarbures existant en Côte d'Ivoire y compris sur le plateau continental.

**Article 69**

L'Etat et les collectivités doivent veiller ' la ci cation, au maintien et à l'entretien d'espaces verts.

**Article 70**

La gestion des eaux usées relève de la compétence de l'Etat, des collectivités locales et de toutes autres structures susceptibles de produire des effluents de nature à porter atteinte à l'environnement.

Elle peut faire l'objet d'une concession.

**Article 71**

L'Etat, les régions, les départements et les collectivités locales s’engagent à élaborer des programmes d'action et à organiser des plans d'urgence dans tous les domaines en vue de protéger l'environnement.

**Article 72**

L'éducation, la formation et la sensibilisation environnementales incombent à l'Etat, aux collectivités locales et aux associations de défense.

**Article 73**

Les établissements et institutions publics ou privés ayant en charge l'enseignement, la recherche et l'information sont tenus dans le cadre de leurs compétences respectives :

- de sensibiliser aux problèmes d'environnement par des programmes adaptés

- d'intégrer dans leurs activités des programmes permettant d'assurer une meilleure connaissance de l'environnement.

**Section IV : Les Institutions**

**Article 74**

Pour l’application de la présente loi, il est créé

- un Réseau de Réserves Biologiques en proportion avec l'intensification de l'exploitation des sols

- un Observatoire de la Qualité de L’Air ;

- une Agence Nationale de l'Environnement (ANDE) , établissement public de catégorie particulière dotée de la personnalité morale et de l'autonomie financière

- un Fonds National de l'Environnement (FNDE)

- une Bourse de Déchets

Par ailleurs, le juge des référés est compétent pour constater ou, faire cesser immédiatement toute pollution ou toute forme de, dégradation de l'environnement.

La procédure d'urgence prévue aux articles 221 à 230 du Code de Procédure Civile, Commerciale et Administrative est applicable.

## TITRE V : DISPOSITIONS PREVENTIVES ET DISPOSITIONS PENALES

## CHAPITRE 1: DISPOSITIONS PREVENTIVES

### Article 75

Sont interdits :

les déversements, les rejets de tous corps solides, de toutes substances liquides, gazeuses, dans les cours et plans d'eaux et leurs abords ;

toute activité susceptible de nuire à la qualité de l'air et des eaux tant de surface que souterraines.

### Article 76

Il est interdit de rejeter dans les zones maritimes et lagunaires, toutes substances susceptibles de :

- détruire les sites et monuments présentant un intérêt scientifique, culturel, touristique ou historique ;

- détruire la faune et la flore

- constituer un danger pour la santé des êtres vivants ;

- porter atteinte à la valeur esthétique et touristique de la lagune, de la mer et du littoral .

### Article 77

Il est interdit de rejeter dans les eaux maritimes et lagunaires

des eaux usées , à moins de les avoir préalablement traitées conformément aux normes en vigueur ;

- des déchets de toutes sortes non préalablement traités et nuisibles.

### Article 78

Il est interdit de détenir ou d'abandonner des déchets susceptibles de :

- favoriser le développement d'animaux vecteurs de maladies

- provoquer des dommages aux personnes et aux biens.

### Article 79

Sont interdits :

- tous déversements, écoulements, rejets ou dépôts de toutes natures susceptibles de provoquer ou d'accroître la pollution des eaux continentales, lagunaires et maritimes dans les limites territoriales ;

- toute exploitation illégale, dégradante et/ou non réglementée ;

- toute émission dans l'atmosphère de gaz toxiques**,** fumée, suie**,** poussière ou toutes autres substances chimiques non conformes à la réglementation en vigueur**.**

**Article 80**

Conformément aux dispositions spéciales des conventions internationales ratifiées par la Côte d'Ivoire, sont interdits les déversements, les immersions et incinérations dans les eaux maritimes sous juridiction ivoirienne de substances de toutes natures susceptibles :

- de porter atteinte à la santé publique et aux ressources maritimes biologiques

- de nuire aux activités maritimes y compris la navigation et la pêche

- d'altérer la qualité des eaux maritimes

- de dégrader les valeurs d'agréments et le potentiel touristique de la mer et du littorale

**Article 81**

Sont interdits :

- l'importation non autorisée de déchets sur le territoire national ;

- les dépôts de déchets sur le domaine public non autorisé, y compris le domaine public maritime tel que défini par les textes en vigueur ;

- l'immersions l'incinération ou l'élimination par quelque procédé que ce soit, des déchets dans les eaux continentales, lagunaires et maritimes, sous juridiction ivoirienne.

**Article 82**

Sont interdits sur le territoire national, tous actes relatifs l'achat, à la vente, à l'importation, à l'exportation et au transit des substances ou combinaison de substances visées à l'article 8 de la présente loi..

**Article 83**

Sont interdites, si elles n'ont pas fait l'objet d'une homologation et/ou si elles ne bénéficient pas d'une autorisation provisoire de vente, d'importation, d'exportation délivrée par les autorités compétentes, toute importation, exportation, détention en vue de la vente ou de la mise en vente, de distribution même à titre gratuit, de l'une quelconque des matières fertilisantes définies à l'article 1er de la présente loi..

**Article 84**

L'usage de l'avertisseur sonore est interdit dans les agglomérations et aux environs des hôpitaux et des écoles sauf nécessité absolue et dans ce cas, il doit être bref et modéré.

De même sont interdites les émissions de bruits, de lumières et d'odeurs susceptibles de nuire à la santé des êtres vivants ou de constituer une gêne excessive et insupportable pour le voisinage ou d'endommager les biens.

**Article 85**

Tout affichage est interdit sur :

- les immeubles classés monuments historiques ou inscrits
- les monuments naturels et dans les sites classés, inscrits ou protégés ;
- les monuments, sites et les constructions dont la liste est établie par les autorités compétentes, bénéficiant d'une protection spéciale
- les panneaux de signalisation routière.

**Article 86**

Sont interdits :

- usage d'explosif, de drogues, de produits chimiques ou appâts dans les eaux de nature à enivrer le poisson ou à le détruire ;

- l'emploi de drogues, de produits chimiques ou appâts de nature à détruire le gibier et/ou à le rendre impropre à la consommation ;

- les feux de brousse non contrôlés.

**Article-87**

Il est interdit de :

- tuer, blesser ou capturer les animaux appartenant, aux espèces protégées

- détruire ou endommager les habitats, les larves, et les jeunes espèces protégées ;

- faire périr, endommager les végétaux protégés, en cueillir tout ou partie ;

- transporter ou mettre en vente tout ou partie d'un animal ou d'un végétal protégé ;

- procéder à l'abattage d'arbres dans les forêts classées, aires protégées et parcs nationaux.

## CHAPITRE II : DISPOSITIONS PENALES

**ARTICLE 88**

Toute personne morale ou physique, qui omet de faire une étude d'impact environnemental prescrite par l'autorité compétente et préalable à tout projet susceptible d'avoir des effets nuisibles sur l'environnement, est passible de suspension d'activité ou de fermeture d'établissement sans préjudice des mesures de réparation des dommages causés à l'environnement, aux personnes et aux biens.

La falsification d'une étude d'impact environnemental et/ou sa non conformité sont punies des mêmes peines.

**Article 89**

Est puni d'un emprisonnement de deux mois à deux ans et d'une amende de 5000000 de francs, quiconque procède ou fait procéder à l'abattage d'arbres ou d'animaux dans les forêts classées, les aires protégées et les parcs nationaux.

Les complices sont punis des mêmes peines.

**Article 90**

Est puni d'une amende de 10000000 de francs à 100000000 de francs et d'un emprisonnement de six mois à deux ans ou de l'une de ces deux peines seulement, toute destruction de site ou monument classé.

**Article 91**

Est puni d'un emprisonnement de un  à six mois et d'une amende de 1000000 de francs à 5000000 de francs ou de. 1'une de ces deux peines seulement : tout responsable d'un établissement faisant obstacle a l'exercice des fonctions des agents chargés de l'inspection des installations classées.

En cas de récidive, il est procédé à la fermeture temporaire de l'établissement.

**Article 92**

Est passible d'une amende de 5000000 de francs à 50000000 de francs sans préjudice d'une suspension temporaire des activités, ou d'une fermeture de l'établissement, tout établissement qui ne se sera pas mis en conformité avec les dispositions de la présente loi dans les deux ans de sa promulgation.

**Article 93**

Quiconque poursuit l'exploitation d'une installation classée sans se conformer à la mise en demeure d'avoir à respecter les prescriptions techniques déterminées est puni d'un emprisonnement d'un mois à un an et d'une amende de 200000 francs à 2000000 de francs.

**Article 94**

Quiconque poursuit l'exploitation d'une installation classée frappée de fermeture, de suspension ou d'interdiction est puni de deux mois à deux ans d'emprisonnement et de 50000000 de francs à 100000000 de francs ou de l’une de ces deux  peines seulement.

**Article 95**

Est puni d'une amende de 1000000 de francs à 2500000 de francs et d'un emprisonnement de six mois à deux ans ou de l'une de ces deux peines seulement quiconque se livre de façon illicite à des travaux de recherches ou d'exploitation des hydrocarbures.

**Article 96**

Est passible d'une amende de 100000000 de francs à 500000000 de francs quiconque effectue des rejets interdits ou, sans autorisation, des rejets soumis à autorisation préalable ainsi que défini aux articles 74 à 86 du présent code dans les conditions fixées par décret ou ne se conforme pas aux conditions déterminées par son autorisation.

**Article 97**

Est puni d'une amende de 2000000 de francs à 50000000 de francs et d'un emprisonnement de deux mois à deux ans ou de l'une de ces deux peines seulement, toute personne ayant pollué les eaux continentales par des déversements, écoulements, rejets et dépôts de substances de toute nature susceptible de provoquer ou d'accroître la pollution des eaux continentales et/ou des eaux maritimes dans les limites territoriales.

En cas de récidive, la peine est portée au double. Le coupable peut être condamné à curer les lieux pollués.

L'Autorité Nationale Compétente peut, en cas de négligence, refus ou résistance, y procéder ou y faire procéder aux frais et dépens de l'intéressé.

**Article 98**

Est puni d'une amende de 100000000 de francs à 1000000000 de francs et d'un emprisonnement de un à cinq ans ou de l'une des deux peines seulement sans préjudice des sanctions administratives en vigueur, quiconque, nonobstant les dispositions spéciales des conventions internationales, procède à des déversements, immersion et incinération dans les eaux maritimes sous juridiction ivoirienne, des substances de toutes natures susceptibles

- de porter atteinte à la santé publique et aux ressources maritimes biologiques ;

- de nuire aux activités maritimes y compris la navigation et la pêche

- d'altérer la qualité des eaux maritimes ;

- de dégrader les valeurs d'agrément et le potentiel touristique de la mer et du littoral.'

L'administration maritime peut arraisonner tout navire surpris en flagrant délit de déversement de contaminants, y compris les hydrocarbures en mer.

En cas de récidive, l'amende est portée au double et L'Administration se réserve le droit de procéder à la saisie du navire.

**Article 99**

Est passible d'un emprisonnement de 1 à 5 ans et d'une amende de 5000000 de francs à 100000000 de francs quiconque :

- dépose des déchets dans le domaine public maritime national ;

- importe sans autorisation des déchets sur le territoire national ;

- immerge, incinère ou élimine par quelque procédé que ce soit des déchets dans les eaux continentales, lagunaires et/ou maritimes sous juridiction ivoirienne.

**Article 100**

Est puni d'une amende de 1000000 de francs à 30000000 de francs et d'un emprisonnement de trois à vingt-quatre mois ou de l'une de ces deux peines seulement, le promoteur de toute entreprise procédant des dépôts sauvages.

L'autorisation d'exercer toute activité de collecte de déchets sur le territoire national peut être suspendue pour une période d'au moins deux ans.

**Article 101**

Quiconque procède ou fait procéder à l'achat, à la vente à l'importation, au transit, au stockage, à l'enfouissement ou au déversement sur le territoire national de déchets dangereux ou signe un accord pour l'autorisation de telles activités, est puni d'un emprisonnement de 10 à 20 ans et d'une amende de 500000000 de francs à 5000000000 de francs.

La juridiction ayant prononcé la peine peut

- ordonner la saisie de tout moyen ayant servi à la commission de l'infraction ;

- ordonner la saisie et l'élimination des déchets aux frais dépens du propriétaire desdits déchets.

**Article 102**

Est puni d'une amende de 1000 francs à 10000 francs celui qui dépose, abandonne, jette des ordures, déchets, matériaux, ou verse des eaux usées domestiques en un lieu public ou privé sauf si le dépôt a lieu à un emplacement désigné à cet effet par l'Autorité Compétente.

De même est soumise à ces peines et/ou astreinte au nettoyage des lieux, toute personne qui pollue par des déjections un domaine public ou privée.

Sont punis d'une amende de 1000 francs à 10000 francs ou astreint. au nettoyage des lieux, ceux qui auront pollué par des déchets humains un bien public ou privé sauf si ces emplacements sont désignés à cet effet par l'autorité compétente.

**Article 103**

Est passible d'une amende de 10000 francs à 500000 de francs quiconque :

- fait usage dans les agglomérations et aux environs des Hôpitaux et des écoles, d'avertisseurs sonores en dehors des cas de danger immédiat;

- fait usage intempestif et sans nécessité absolue, en dehors des agglomérations d'avertisseurs sonores

- fait usage, sans nécessité absolue d'avertisseurs sonores dans la nuit

- émet des bruits susceptibles de causer une gêne aux usagers de la route et aux riverains ;

- utilise des engins à moteur munis d'avertisseurs sonores non conformes au type homologué par les services compétents

- émet des bruits, lumières. ou odeurs susceptibles de nuire à la santé des êtres vivants, de constituer une gêne excessive et insupportable pour le voisinage ou d'endommager les biens.

**Article 104**

Est puni d'une amende de 50000 francs à 5000000 francs et d'un emprisonnement de trois mois au maximum quiconque fait:

- de la publicité sur un immeuble sans l'autorisation du propriétaire et des autorités compétentes

- de l'affichage et des graffitis sur les immeubles classés inscrits ou classés monuments historiques, sur les monuments naturels et dans les sites inscrits ou protégés.

**Article 105**

Les circonstances atténuantes et le sursis ne sont pas applicables aux infractions prévues par le présent code relatives aux déchets dangereux.

**Article 106**

La tentative et la complicité des infractions prévues par le présent code sont punissables des mêmes peines que l'infraction elle-même.

**Article 107**

Les infractions sont constatées sur procès-verbal par les agents assermentés de l'Autorité Nationale Compétente.

**Article 108**

L'administration chargée de l'environnement peut transiger en toute circonstance et à tout moment de la procédure avant toute décision au fond.

La demande de transaction est soumise à l'Autorité Nationale Compétente qui fixe en cas d'acceptation, le montant de celle-ci.

**Article 109**

La poursuite des infractions relevant du présent code obéit aux règles définies par le code de procédure pénale.

**Article 110**

Les collectivités locales, les associations de défense de l'environnement régulièrement déclarées ou toutes personnes doivent saisir l'Autorité Nationale Compétente avant tout recours devant les juridictions

et/ou exercer les droits reconnus à la partie civile en ce qui concerne les faits constituant une infraction relevant de la présente loi et portant un préjudice direct ou indirect aux intérêts collectifs ou individuels.

## TITRE VI : DISPOSITIONS FINALES

### Article 111

Les modalités d'application des dispositions de la présente loi feront l'objet de décrets.

### Article 112

La présente loi abroge toutes les dispositions contraires antérieures.

### Article 113

La présente loi sera publiée au Journal Officiel de la République de Côte d’Ivoire et exécutée comme loi de l'Etat.

Fait à Abidjan, le 3 octobre 1996

Henri Konan BEDIE
