# Supplementary material for: Ivory Coast without ivory: Massive extinction of African forest elephants in Côte d’Ivoire
Source: PLoS One. 2020 Oct 14;15(10):e0232993. doi: 10.1371/journal.pone.0232993 (PMC7556483; doi:10.1371/journal.pone.0232993)
Supplement: S3 File — (PDF) [file pone.0232993.s006.pdf]

N° :…

S3 File : Guide de l'enquête sur les éléphants

Enquêteur :……………………………………………………………………………………..
Nom de l'informateur :……………………….………………Date……………………….
Domicile :………………………………Lieu de l'enquête :……………………………….
Profession :………………………………Village/Campement…………………………….
Situation de la plantation :……………………………………………………………………..
Coordonnées (GPS) de la plantation ou du lieu d'enquête :………………………………
Sous-préfecture :…………………………. Préfecture :……………………………………

01- Avez-vous des problèmes avec les animaux sauvages ?...

02- Lesquels ?...

03- Avez-vous déjà vu des éléphants ?...

04- Combien étaient-ils ?..

05- Combien de groupes y avait-ils ?...

05- Combien d'individus par groupe ? ……………………………………………………..

06- Quelle est leur taille ? (en mettre ou par rapport à la taille de l'homme)............................

07- Où les avez-vous vu ?..

08- Quand les avez-vous vus ? ....

	1ère fois	2ème fois	3ème fois
a- Quand précisément (mois, semaine)			
b- Où ?			
c- En avez-vous entendu parler			
d- En avez-vous vu ?			
e- En avez-vous vu des traces ?			
f- Combien étaient-ils (nombre) ?			

09- Dans quelles régions peut-on facilement rencontrer les éléphants ?....................................

10- Selon vous, pourquoi sont-ils fréquents dans ces régions ?..

11- On dit que les éléphants causent beaucoup de dégâts dans les plantations ; sont-ils arrivés dans ta plantation ?..

12- Où se situe ta plantation par rapport à la Réserve ?...

13- Quelle distance sépare ta plantation de la Réserve ?..

14- Combien de fois les éléphants sont-ils arrivés dans ta plantation ?.....................................

15- D'où viennent-ils ?..

16- Où vont-ils après ?...

17- Combien étaient-ils ? (chacune des fois)…………………………………………………

18- Que faisaient-ils ? (chacune des fois)……………………………………………………

19- Combien de mâles y avait-il ? (chacune des fois)…………………………………………

20- Combien de femelles y avait-il ? (chacune des fois)...

21- Combien de jeunes y avait-il ? (chacune des fois)..

22- Combien de petits y avait-il ? (chacune des fois)..

23- Lesquels étaient devant ? (chacune des fois)...

24- Lesquels étaient derrière ? (chacune des fois)……………………………………………

25- Lesquels étaient au milieu ? (chacune des fois)...

26- Y a-t-il des éléphants qui caractérisent ces troupeaux ?.............................. ……………..

27- Quelles sont leurs caractéristiques (nombre, taille, couleur, forme oreilles, forme des défenses, etc.) ?

…………………………………………………………………………………………………

…………………………………………………………………………………………………

28- Quand est ce que les éléphants arrivent dans ta plantation ? (chacune des fois) :

Saison	Lune claire	Lune noire	Matin	Après-midi	Nuit (soir)	Nuit (matin)	Durée	Nombre de fois
a. Pluvieuse								
b. Sèche								
c. Période de Récolte								

29- Autre (préciser) : ………………………………………………………………………..

…………………………………………………………………………………………………

30- Pourquoi les éléphants arrivent-ils ici (chez vous) ?...

………………………………………………………………………………………………..

31- Quelles sont les cultures que les éléphants détruisent (les énumérer par ordre d'importance par rapport aux superficies ou aux productions) ?

**Saison**	**Cultures détruites par les éléphants**					
Pluvieuse						
Sèche						
Période de récolte						
Quantité avant les dégâts (bon, moyen, faible)						
Maturité des cultures (jeune, inter, mature)						

	**Superficie/ Production**						
**Cultures**							
Superficies totales							
Superficie détruite							
Production obtenue (t)							
Production attendue (t)							
Revenu attendu (FCFA)							
Perte en FCFA ou en tonne							

32- Autres dégâts

a- Réserve de vivres	
b- Réserve d'eau	
c- Menace de vie humaine	
d- Blessure humaine	
e- Mort d'homme	
f- Autres (préciser)	
g- Blessure ou mort éléphant	

33- Avez-vous déposé des plaintes ?..

Rapport N°	1	2	3	4	5
a- Envoyé ? o/n					
b- à qui ?					
c- Quand ?					
d- Où ?					
e- Comment ?					

34- Avez-vous reçu des réponses à vos plaintes (oui/non) ?..

Rapport N°	1	2	3	4	5
a- Résultats obtenus ?					
b- Satisfaits ?					

35- **Les éléphants arrivent-ils dans les plantations de tes voisins ?.....................................**

36- Quand ? (chacune des fois)……………………………………………………………

37- D'où viennent-ils ?...

38- Où vont-ils après ?...

39- Quelles méthodes utilisez-vous pour éloigner les éléphants de vos plantations ?...........

………………………………………………………………………………………..

………………………………………………………………………………………..

40- Ces méthodes sont-elles efficaces ?..

41- Pourquoi ?...

42- Quel est le temps utilisé ?..

43- Quels sont les moyens utilisés ?

a- Humains……………………………………………………………………

b- Matériel……………………………………………………………………

c- Autres………………………………………………………………

44- Connaissez-vous d'autres méthodes que vous n'aviez pas encore expérimentées ?........

a- Oui/ non……………………………………………………………………….

b- Lesquelles ?..

45- Certains de vos biens autres que les cultures sont-ils détruits par les Éléphants ?

a- Lesquels						
b- Quand ?						
c- Valeur/coût						

46- Savez-vous que l'éléphant joue un rôle important dans la nature pour l'homme ?....

47- Citez des exemples que vous connaissez

………………………………………………………………………………………..

………………………………………………………………………………………..

………………………………………………………………………………………..

48- Êtes-vous favorables à la conservation des Éléphants ?...

………………………………………………………………………………………..

49- Quels conseils pouvez-vous nous donner pour protéger vos cultures ?...........................

……………………………………………………………………………………….

……………………………………………………………………………………….

50- Comment pouvez-vous nous aider à atteindre ces objectifs ?

……………………………………………………………………………………….

……………………………………………………………………………………….

51- Que savez-vous des comportements des Éléphants ?

……………………………………………………………………………………….

………………………………………………………………………………………..

52- Existe-t-il d'autres animaux qui détruisent vos cultures ?...

53- Énumérez-les par ordre d'importance…………………………………………………

…………………………………………………………………………………………

…………………………………………………………………………………………

54- Quand détruisent-ils vos cultures ?...

…………………………………………………………………………………………

…………………………………………………………………………………………..

55- À qui exposez-vous les problèmes de destruction de vos cultures (par les autres animaux) ?

………………………………………………………………………………………………..

56- Comment vous aident-ils à résoudre ces problèmes ?...

………………………………………………………………………………………………..

………………………………………………………………………………………………..

57- Êtes-vous satisfaits des solutions qu'ils vous apportent ?..

………………………………………………………………………………………………..

……………………………………………………………………………………………..

58- Êtes-vous déjà allés vous plaindre aux gestionnaires de ces animaux ?............................

59- Combien de fois ?...

60- Qu'ont-ils fait ?..

………………………………………………………………………………………………..

………………………………………………………………………………………………..

61- Étiez-vous satisfaits ?...

62- Pourquoi ?..

63- Que souhaiterez-vous qu'ils fassent ?...

………………………………………………………………………………………………..

……………………………………………………………………………………………….

64- Que reprochez-vous au gouvernement dans ce problème d'éléphant ?...........................

………………………………………………………………………………………………..

……………………………………………………………………………………………….

65- Qu'est-ce que vous vous reprochez ?..

………………………………………………………………………………………………..

66- Quels sont les chemins empruntés par les Éléphants pour arriver :

a- dans vos plantations ?..

b- Dans la Réserve ?..

67- Connaissez-vous une histoire sur ces animaux (déplacements, problèmes causés, etc.) ?

………………………………………………………………………………………………..

………………………………………………………………………………………………..

68- La présence de ces éléphants a-t-elle modifié:

a- Les spéculations pratiquées ?…………………………………..……………..………

b- Vos dates de semis (calendrier cultural) ?……………………………..……………

c- Vos pratiques agricoles ?………………………………………..……………………

d- Vos habitudes alimentaires ?………………………………...………………………

e- Votre mode de vie de façon générale ?…………….…………….…………..……….

………………………..………………………………………………………………………..

Autres informations

……………………………………………………………………………...…………………

……………………………………………………………………………..………………

………………………………………………………………………………………………

……………………………………………………………………………………………..

……………………………………………………………………………………………..

……………………………………………………………………………………………..

……………………………………………………………………………………………..

……………………………………………………………………………………………..

……………………………………………………………………………………………..

……………………………………………………………………………………………..

……………………………………………………………………………………………..

……………………………………………………………………………………………..
