# Supplementary material for: Ivory Coast without ivory: Massive extinction of African forest elephants in Côte d’Ivoire
Source: PLoS One. 2020 Oct 14;15(10):e0232993. doi: 10.1371/journal.pone.0232993 (PMC7556483; doi:10.1371/journal.pone.0232993)
Supplement: S4 File — (PDF) [file pone.0232993.s007.pdf]

S4 File : Fiches de collecte de données

Date : ...... /…… / 2019 Site visitée ……………………… N° layon :…………… Point départ : ……………………………

Coordonnées GPS départ : ……………….… /………..……… Direction départ :……...... Météo ………… (Ens. ou Couv ou Pluv.)

<u>Rapporteur</u> : ………………………… Membres de l'équipe : ……...…………………………………………………..........................

Heure	Distance parcourue (m)	Type de végétation (habitat)	Objet ou code de l'observation	Type observation	Nombre objet détecté ou taille groupe	Stade ou âge relatif indice (nids/crotte)	Position géographique				Distance perpendiculaire (m)
							Numéro position GPS	Longitude (UTM)	Latitude (UTM)	Altitude (m)	

<u>Stade des crottes</u> : **S1**, crotte fraîche, très odorante, humide ; **S2**, crotte fraîche, surface un peu sèche et pas humide, pas odorante ; **S3**, la moitié de la crotte est décomposée, les boules de crottes sont décomposées et forment une masse plus ou moins aplatie ; **S4**, toutes les boules de crottes sont désagrégées, la matière fécale a disparu.
