# Supplementary material for: Ivory Coast without ivory: Massive extinction of African forest elephants in Côte d’Ivoire
Source: PLoS One. 2020 Oct 14;15(10):e0232993. doi: 10.1371/journal.pone.0232993 (PMC7556483; doi:10.1371/journal.pone.0232993)
Supplement: S5 File — (PDF) [file pone.0232993.s008.pdf]

MINISTERE DES EAUX ET FORETS

DIRECTION GENERALE
DES EAUX ET FORETS

DIRECTION DE LA FAUNE ET
DES RESSOURCES CYNEGETIQUES

SOUS-DIRECTION DE LA FAUNE
ET DE LA CHASSE

REPUBLIQUE DE COTE D'IVOIRE
Union – Discipline - Travail

TEXTES REGISSANT LA PROTECTION DE LA FAUNE ET L'EXERCICE DE LA CHASSE

Mai 2013

SOMMAIRE

Loi n° 94-442 du 16 août 1994 portant modification de la loi n° 65-255 du 4 août 1965 relative à la protection de la faune et à l'exercice de la chasse …………………………………………………………..........................1

Loi n° 65-255 du 4 août 1965 relative à la protection de la faune et à l'exercice de la chasse…………………..6

Décret n° 97-130 du 07 mars 1997 réglementant la détention des ivoires………………………………………18

Décret n° 66-423 du 15 septembre 1966 fixant le régime des permis de chasse et les modalités de leurs attributions en République de Côte d'Ivoire……………………………………………………………………22

Décret n° 66-424 du 15 septembre 1966 relatif à la licence de guide de chasse……………………………..30

Décret n° 66-425 du 15 septembre 1966 réglementant le trafic, la circulation, l'importation, l'exploitation des trophées d'animaux protégés et spectaculaires et de leurs dépouilles……………………………………………35

Décret n° 66-433 du 15 septembre 1966 portant statut et réglementation de la procédure de classement des réserves naturelles, intégrales ou partielles et des parcs nationaux………………………………………………37

Arrêté n° 1712 AGRI/EFC du 29 décembre 1966 fixant les conditions d'élimination ou d'éloignement des animaux nuisibles……………………………………………………………………………………………..41

Arrêté n° 621 AGRI/EFC du 29 mai 1967 réglementant la destination des produits de la chasse……………..44

Arrêté n° 1068 du 29 septembre 1967 réglementant la chasse des crocodiles et varans dans un but commercial…………………………………………………………………………………………………….45

Arrêté n° 1069 du 29 décembre 1967 réglementant la détention des animaux vivants par des particuliers……………………………………………………………………………………………………46

Arrêté n° 68 du 23 janvier 1967 fixant les tarifs des taxes et redevances en matière de chasse et de capture des animaux sauvages ……………………………………………………………………………………48

Arrêté n° 15 SEPN / SEB du 26 décembre 1972 portant modification de l'Arrêté n° 68 du 23 janvier 1967 fixant les tarifs des taxes et redevances en matière de chasse et de capture des animaux sauvages……………………………………………………………………………………………………….50

Arrêté n° 003/SEPN/CAB du 20 Février 1974 portant fermeture de la chasse…………………………………..52

Loi n° 94-442 du 16 août 1994 portant modification de la loi n° 65-255 du 4 août 1965 relative à la protection de la faune et à l'exercice de la chasse

L'ASSEMBLEE NATIONALE A ADOPTE

LE PRESIDENT DE LA REPUBLIQUE PROMULGUE LA LOI DONT LA TENEUR SUIT :

Article premier :
L'article 4 de la loi n° 65-255 du 4 août 1965 relative à la protection de la faune et à l'exercice de la chasse est ainsi complété :

Article 4 nouveau :
La protection de faune est assurée par les processus ci-après :

1. Constitution et entretien de réserves naturelles intégrales ou partielles et de parcs nationaux, tels que définis à l'article 2 de la Conservation internationale de Londres du 8 novembre 1933 relative à la conservation de la faune et de la flore africaines à l'état naturel ;
2. Constitution et entretien de réserves totales ou partielles de faune établies soit pour toute la faune, soit pour certaines espèces seulement ou dans certaines conditions ;
3. Détermination et aménagement de zones à vocation faunique ;
4. Protection intégrale ou partielle des espèces animales rares ou menacées d'extinction, ou présentant un intérêt scientifique, ou nécessaire à l'équilibre biologique, ou particulièrement utiles à l'homme, ou dont l'exploitation pour le tourisme cynégétique ou visuel comporte un intérêt économique ou éducatif ;
5. Mesures techniques de limitation de l'exercice de la chasse notamment protection des femelles et des jeunes, des œufs et couvées, interdiction de chasser de nuit, fixation de périodes de fermeture, limitation des tableaux de chasse, limitation du nombre des armes ou de l'emploi de certaines armes ;
6. Interdiction de certains moyens de chasse et notamment véhicules à moteur terrestres ou aériens, feux encerclant, lumières éblouissantes, poisons, stupéfiants, explosifs, filets, fosses et pièges ;
7. Surveillance exercée par un personnel spécialisé avec l'aide des différents services ayant attributions de contrôle de répression : Police Forestière, Gendarmerie, Douane, Polices nationale et municipale ;
8. Répression dont l'efficacité sera recherchée par l'application des présomptions légales de culpabilité, par l'exemplarité des peines et par la rapidité d'intervention ;
9. Education globale de la population tant par l'enseignement aux différents degrés que par les moyens audiovisuels en vue de susciter une prise de conscience nationale de la notion de protection de la nature ;
10. Elevage d'animaux sauvages en des lieux fixes spécialement aménagés.

Article 2 :
L'article 9 de la loi n° 65-225 du 4 août 1965 est abrogé et remplacé par l'article 9 nouveau :

Article 9 nouveau :
Il est créé cinq catégories de permis :
1. Les permis de petite chasse qui comportent deux degrés ;
2. Le permis local à l'échelon de la sous-préfecture, au bénéficie exclusif de cultivateurs ou éleveurs titulaires d'un permis de port d'armes de traite et ne donnant le droit de chasser que les animaux cités aux annexes III de la présente loi et ceci hors des zones d'aménagement faunique prévues à l'article 18 ;
3. Le permis national valable pour les animaux non protégés donnant le droit de chasser avec une arme perfectionnée, sur l'ensemble du territoire, les animaux des annexes II et III, dans les limites prévues à ces annexes ;

4. Les permis de chasse sportive autorisant l'abattage d'un nombre déterminé d'animaux dits partiellement protégés et comportant trois degrés ;
5. Le permis de moyenne chasse ;
6. Le permis de chasse touristique de passager de courte durée ;
7. Le permis de grande chasse ;
8. Les permis de capture commerciale autorisant la capture, la détention, la cession, l'exportation des animaux sauvages vivants à l'exclusion des espèces intégralement protégés ;
9. Les permis scientifiques de chasse ou de capture accordés exceptionnellement à des représentants d'organismes scientifiques officiels pour l'abattage ou la capture à des fins scientifiques précises d'animaux d'espèces intégralement protégés ;
10. Les permis de chasse d'animaux sauvages d'élevage ;

Article 3 :
L'intitulé du chapitre V et les dispositions des articles 15 et 16 sont modifiés comme suit :

CHAPITRE V (NOUVEAU)
Produits de la chasse

Article 15 nouveau :
L'autorité administrative compétente réglemente, si besoin est, la commercialisation, l'importation et l'exportation des dépouilles d'animaux sauvages et notamment des trophées.
L'expression «trophées» désigne tout animal mort mentionné aux annexes I et II ou toute partie non périssable ou naturalisée de cet animal, incorporés ou non dans un objet travaillé.
L'expression «viande» désigne la viande fraîche ou conservée, la graisse et le sang des animaux sauvages.

Article 16 nouveau :
L'autorité compétente fixe les conditions dans lesquelles elle autorise la commercialisation sous toutes ses formes de la viande de chasse.

Article 4 :
L'article 39 de la loi n° 65-255 du 4 août 1965 est ainsi complété :
Les conditions d'application de la présente loi sont déterminées par décret et notamment en ce qui concerne :
- La constitution des réserves, des parcs nationaux et des zones d'aménagement faunique ;
- La représentation de l'Administration devant les tribunaux ;
- La procédure applicable en matière de transaction ;
- Les conditions de délivrance des permis de chasse et de capture, des licences de chasse et les modalités de concession du droit de chasse ;
- Les conditions de création et d'exploitation des fermes d'élevage d'animaux sauvages.

Article 5 :
Les annexes à la loi n° 65-255 du 4 août 1965 sont abrogées et remplacées par les annexes nouvelles ci-après.

Article 6 :
La présente loi sera exécutée comme loi de l'Etat et publiée au Journal Officiel de la République de Côte d'Ivoire.

Fait à Abidjan, le 16 août 1994.

Henri Konan BEDIE.

ANNEXES

à la loi n°94-442 du 16 août 1994 portant modification de la loi n° 65-255 du 04 août 1965 relative à la protection de la faune et à l'exercice de la chasse.

ANNEXE I
ESPECES INTEGRALEMENT PROTEGEES

Animaux sauvages intégralement protégés dont la capture et la chasse (y compris celles de leurs jeunes ou de leurs œufs) sont interdits sauf aux porteurs de permis scientifiques dans les limites et avec les moyens inscrits au permis :

Mammifères

- Eléphant (Loxodonta africana africana, Loxodonta africana cyclotis) ;
- Hippopotame nain (Choeropsis liberiensis) ;
- Lamatin (Trichechus senegalensis) ;
- Chimpanzé (Pan troglotydes) ;
- Chevrotain aquatique (Hyemesenus aquaticas) ;
- Colobe magistrat (Colobus polykomos) ;
- Céphalophe zèbre (Cephalophus zebra) ;
- Pangolin terrestre géant (Manis gigantea) ;
- Microprotamogale (Microprotamogale lamottei);
- Antilope royale (Neotragus pygmeus) ;
- Hylochère (Hylochoerus meinerzhageni) ;
- Potto de Bosman (Perodictucus potto) ;
- Galago de Demidof (Galago demidovii) ;
- Bongo (Tragelaphus euryceros) ;
- Léopard (Phanthera pardus) ;
- Lion (Panthera leo) ;
- Cercopithèque Diane (Cercopithecus diana) ;
- Colobe Bai (Colobus badius badius) ;
- Oryctérope (Orycteropus afer) ;
- Cephalophe de Jentink (Cephalophus jentinki) ;
- Céphalophe à dos jaune (Cephalophus sylvicultor).

Reptiles

- Crocodiles du Nil (Crocodylus niloticus) ;
- Crocodiles à long museau (Crocodylus cataphractus) ;
- Crocodiles de forêt ou de marais (Osteolaemus tetraspis) ;
- Tortues marines (Chelonidae)

Oiseaux

- Petit serpentaire (Polyboroides radiatus) ;
- Pintade à poitrine blanche (Agelastes meleagrides) ;
- Tous les vautours ;
- Grand calao d'abyssinie (Bucorvus abyssinicus) ;
- Marabout (Leptoptilos crumeniferus) ;
- Grande aigrette (Egretta aiba) ;
- Aigrette garzette forme blanche (Egretta garzetta) ;
- Aigrette garzette forme grise (Egretta gularis) ;
- Grue couronnée (Balearica pavonina) ;
- Jabiru (Ephippiorhynchus senegalensis) ;
- Tous les Hérons, Cigognes et Ibis ;
- Tous les Aigles.

ANNEXE II

Liste des animaux partiellement protégés dont la chasse et la capture sont autorisées aux titulaires de permis de chasse sportive ou permis de capture dans les limites indiquées aux permis :

Mammifères

- Pangolin à écailles tricuspides (Manis tricuspis) ;
- Pangolin à longue queue (manis tetradactyla) ;
- Anomalures nain (Idiurus macrotis) ;
- Buffle (Syncerus caffer) ;
- Hippotrague (Hippotragus equinus) ;
- Cobe defassa (Kobus ellispsiprymnus defassa) ;
- Bubale (Alcephalus buselaphus) ;
- Lycaon (Lycaon pictus) ;
- Hyène tachetée (Crocuta crocuta) ;
- Chacal à flans rayés (Canis adustus) ;
- Serval (Leptairus serval liposticta) ;
- Loutre à joues blanches (Aonyx capensis) ;
- Ratel (Meilivora capensis) ;
- Zorile commun (Ictonyx striatus) ;
- Nandinie (Nandinia binotata) ;
- Mone (Cercopithecus mona) ;
- Singe vert (Cercopithecus aethiops) ;
- Céphalophe noir (Cephalophus niger) ;
- Potamochère (Potamochoerus porcus).

Reptiles

- Python de Séba (Python sebae) ;
- Python royal (Python regius).

Oiseaux

- Poule suitane (Porphyrio porphyrio) ;
- Jacana (Actophilomis africana) ;
- Tous les Rapaces diurnes sauf Serpentaires, Vautours, aigles, Accipitriformes ;
- Rapaces nocturnes : tous les Strigiformes ;
- Perroquets : tous les Psittaciformes ;
- Touracos, Musophages, Coucous ;
- Couroucou à joues vertes (Apaloderma narina) ;
- Tous les Pics et Barbus ;
- Tous les Martins pêcheurs, Rolliers, Calaos (sauf le grand calao et Guêpiers)
- Merie métallique, Loriot et Souimangas.

ANNEXE III

Animaux sauvages dont la chasse autorisée pour les usagers coutumiers, pour les titulaires de permis de petite chasse et de permis spéciaux sportifs dans les limites des latitudes générales de chasse autorisées par la loi :

Mammifères

- Guib harnaché (Tragelaphus scriptus) ;
- Cobe de buffon (Kobus kob)
- Redunca (Redunca redunca) ;
- Céphalophe de grimm ou biche cochon (Cephalophus grimmia) ;
- Céphalophe à bande dorsale noire (Cephalophus niger) ;
- Céphalophe à flanc roux (Cephalophus rufilatus) ;
- Céphalophe de maxwell (Cephalophus maxwelli) ;
- Ourébi (Ourebia ourebi) ;
- Phacochère (Phacochoerus aethiopicus) :
- Daman d'arbre (Dendrohyrax arboreus) ;
- Lièvre (Lepus whytei) ;
- Aulacode (Tryonomis swinderianus) ;
- Porc-épic (Hystrix cristata) ;
- Atherure (Atherurus africanus) :
- Tous les Ecureuils ;
- Hérisson à ventre blanc (Erinaceus aibiventris) ;
- Chat doré (Profelis aurata) ;
- Chat sauvage (Felis silvestris) ;
- Civette (Viverra civetta) ;
- Genettes (Genette pardina, Genetta tigrina) ;
- Poiane (Poiana richardsoni) ;
- Mangouste ichneumon (Herpestes ichneumon) ;
- Mangouste rouge (Herpestes sanguineus) ;
- Mangouste brune (Mungos obscurus) ;
- Mangue (Mungos gambianus) ;
- Cynocéphale (Papio cynocephalus) ;
- Patas (Erythrocebus patas) ;
- Hocheur (Cercopithecus petaurista) ;
- Potamogale (Potamogale velox) ;

Reptiles

- Toutes les Tortues (sauf Chelonidae) ;
- Varan de savane (Varanus exanthematicus) ;

Oiseaux

- Tous les : Canards, Oiseaux, Sarcelles ;
- Tous les : Francolins, Pintades, Caille, Poule de roche ;
- Tous les : Pluviers, Vanneaux, Chevaliers, Courlis, Oedicnèmes, Bécassines.

Loi n° 65-255 du 4 août 1965 relative à la protection de la faune et à l'exercice de la chasse

L'ASSEMBLEE NATIONALE A ADOPTE,

LE PRESIDENT DE LA REPUBLIQUE PROMULGUE LA LOI DONT LA TENEUR SUIT :

TITRE PREMIER
GENERALITES

Article premier :

- Aux termes de la présente loi et des textes qui seront pris pour son application, la faune est constituée par tous les animaux sauvages vivant en liberté dans leur milieu naturel, classés parmi les mammifères (à l'exception des chauves souris, des rats et des souris), et parmi les oiseaux, les crocodiles, les varans et pythons.

Les animaux qui composent la faune sont répartis comme suit :

- Les espèces dites protégées, classées et énumérées à l'annexe **I**, rares ou menacées d'extinction ou très localisées ou d'intérêt scientifique dont la disparition constituerait une perte irrémédiable, ou présentant une utilité particulière pour l'homme et ses activités y compris l'intérêt de la chasse sportive et de la valeur des trophées ;
- Les espèces dites spectaculaires, énumérées à l'annexe **II**, oiseaux notamment qui, par leur forme, leur habitat, leur mode de vie, constituent la parure de la nature et interviennent ainsi dans l'intérêt touristique des régions où elles vivent ;
- Les espèces dites prédatrices, énumérées à l'annexe **III**, qui participent à l'équilibre biologique dans les zones affectées à la faune ;
- Les espèces dites petit gibier, énumérés à l'annexe **IV**, qui ne sont ni protégées, ni citées dans les catégories précédentes, qui sont recherchées pour la chasse traditionnelle et la petite chasse et qui participent traditionnellement à l'alimentation locale ;
- Les espèces dites nuisibles, qui constituent un danger permanent ou causent des dommages dans les zones d'habitation ou d'exploitation agricole ou pastorale, et qui seront désignées par l'autorité administrative nonobstant leur appartenance aux annexes **III** et **IV**.

Article 2 :

Les animaux tenus en captivité ou les dépouilles des animaux sauvages ne deviennent la propriété des particuliers qui si ces animaux ont été capturés ou tués conformément aux règlements pris pour l'exercice de la capture ou de la chasse, ou pour les éliminations ou destructions dûment autorisées.

Les dépouilles comprennent tout ou partie de l'animal mort et notamment la viande fraîche ou conservée.

TITRE II : PROTECTION DE LA FAUNE

Article 3 :

La protection de la faune tend à assurer la conservation et l'enrichissement qualitatif et quantitatif des animaux des espèces sauvages vivant naturellement dans le pays, tant sur les surfaces relevant du Domaine de l'Etat que sur les terrains des particuliers.

Article 4 :
La protection de la faune assurée par le processus ci-après:

1° Constitution et entretien de réserves naturelles intégrales ou partielles et de parcs nationaux, tels que définis à l'article 2 de la Convention Internationale de Londres du 8 novembre 1933, relative à la conservation de la faune et de la flore africaines à l'état naturel ;

2° Constitution et entretien de réserves totales ou partielles de faune établies soit pour toute la faune, soit pour certaines espèces seulement ou dans certaines conditions ;

3° Détermination et aménagement de zones à vocation faunique ;

4° Protection intégrale ou partielle des espèces animales rares ou menacées d'extinction, ou présentant un intérêt scientifique, ou nécessaire à l'équilibre biologique, ou particulièrement utiles à l'homme, ou dont l'exploitation pour le tourisme cynégétique ou visuel comporte un intérêt économique ou éducatif ;

5° Mesures techniques de limitation de l'exercice de la chasse, notamment protection des femelles et des jeunes, des œufs et couvées, interdiction et chasser la nuit, fixation de période de fermeture, limitation des tableaux de chasse, limitation du nombre des armes ou de l'emploi de certaines armes ;

6° Interdiction de certains moyens de chasse et notamment de véhicules à moteur terrestres ou aériens, feux encerclants, lumières éblouissantes, poisons, stupéfiants explosifs, filets fosses et pièges ;

7° Surveillance exercée par un personnel spécialisé avec l'aide des différents services ayant des attributions de contrôle et de répression : eaux, forêts et chasse, gendarmerie, douane, police nationale et municipale ;

8° Répression dont l'efficacité sera recherchée par l'application des prescriptions légales de culpabilité, par l'exemplarité des peines et par la rapidité d'intervention ;

9° Education globale de la population tant par l'enseignement aux différents degrés que par les moyens audiovisuels en vue de susciter une prise de conscience nationale de la notion de protection de la nature.

Article 5 :
Le classement des réserves naturelles intégrales ou partielles et des parcs nationaux est décidé par décrets.

Les réserves naturelles intégrales et les parcs nationaux sont soustraits à tous droits d'usage et font partie du domaine forestier classé.

Article 6 :
L'autorité administrative compétente fixe les conditions de délivrance des autorisations spéciales écrites dans lesquelles il est interdit de pénétrer, de circuler, y compris par voie aérienne à basse altitude, de camper et d'effectuer toute recherche scientifique dans les réserves naturelles et réglemente la circulation et le campement à l'intérieur des parcs nationaux.

TITTRE III : CHASSE ET CAPTURE

CHAPITRE PREMIER
ACTES DE CHASSE ET DE CAPTURE

Article 7 :
Aux termes de la présente loi, il faut entendre par «chasse» tout acte tendant soit à blesser ou tuer, pour s'approprier ou non tout ou partie de sa dépouille, un animal sauvage au sens de l'article premier de la présente loi, soit à détruire les œufs des oiseaux ou des reptiles cités en ce même article premier.

Est qualifié acte de capture, tout acte tendant à priver de sa liberté, un animal sauvage désigné à l'article premier ou à récolter et retirer hors de leur lieu naturel d'éclosion, les œufs des oiseaux ou des reptiles cités à l'article premier.

Article 8 :
Nul ne peut, en dehors des exceptions prévues aux articles 11 et 12 (chasse traditionnelles) et aux articles 20, 21 et 22 légitime défense, se livrer à aucun acte de chasse ou de capture s'il n'est détenteur d'un permis.

CHAPITRE II
PERMIS DE CHASSE ET DE CAPTURE

Article 9 :
Il est créé quatre catégories de permis:

1° Les permis de petite chasse qui comportent deux degrés :

a) Le permis local à l'échelon de la sous-préfecture au bénéfice exclusif des cultivateurs ou éleveurs titulaires d'un permis de port d'armes de traite et ne donnant le droit de chasser que les animaux cités aux annexes III et IV zones d'aménagements fauniques prévues à l'article 18.
b) Le permis national valable pour animaux non protégés et donnant le droit de chasser avec une arme perfectionnée sur l'ensemble du territoire les animaux, des annexes II, III et IV, dans les limites prévues à ces annexes.

2° Les permis spéciaux de chasse sportive autorisant l'abattage d'un nombre déterminé d'animaux dits partiellement protégés et comportant trois degrés :

a) Le permis de moyen chasse ;
b) Le permis de chasse touristique de passager de courte durée ;
c) Le permis de grande chasse.

3° Les permis spéciaux de capture commerciale autorisant la capture, la détention, la cession, l'exportation des animaux sauvages vivants, à l'exclusion des espèces intégralement protégées.

4° Les permis spécifiques de chasse ou de capture accordés exceptionnellement à des représentants d'organismes scientifiques officiels pour l'abattage ou la capture à des fins scientifiques précises d'animaux d'espèces intégralement protégées.

Article 10 :
Les dispositions relatives à la nature, à l'attribution, aux latitudes d'abattage, au contrôle, à la publicité, à la durée, à la déchéance de ces divers permis ainsi qu'à la qualité et aux responsabilités des titulaires, sont définies par décret.

CHAPITRE III
CHASSE TRADITIONNELLE

Article 11 :
La petite chasse pour les animaux non protégés pratiquée suivant la tradition, hors des réserves et ses zones de protection, avec des armes traditionnelles de fabrication locale à l'exclusion de toute arme à feu et de tout procédé interdit par la présente loi et ses décrets d'application, est qualifiée « chasse traditionnelle ».

Article 12 :
Est considéré comme « chasseur traditionnel» quiconque, dans les limites de la sous-préfecture de son lieu de résidence, chasse pour son alimentation et celle de sa famille, dans les conditions prévues à l'article 11.

Par dérogation à l'article 8, le chasseur traditionnel est autorisé à chasser sans permis en respectant toutefois les périodes de fermeture de la chasse.

CHAPITRE IV
GUIDES DE CHASSE

Article 13 :
Est réputé «guide de chasse» quiconque organise à titre onéreux pour le compte d'autrui, des opérations de chasse ou de capture ou des expéditions de photographe d'animaux sauvages.

Article 14 :
Nul ne peut exercer la profession de guide de chasse s'il n'est titulaire d'une licence spéciale.

CHAPITRE V
PRODUITS DE LA CHASSE
TROPHEES ET DEPOUILLES
VIANDE DE CHASSE

Article 15 :
L'autorité administrative réglemente le trafic, la circulation des trophées d'animaux protégés et spectaculaires et des dépouilles d'animaux non protégés.

L'expression « trophées » désigne tout animal mort ou vif, mentionné aux annexes I et II, ses dents, défenses, cornes, os, écailles, griffes, sabots, peau, poils, œufs, plumage ou toute partie non périssable de l'animal inclus ou non dans un objet travaillé ou transformé, sauf s'ils ont perdu leur identité d'origine ; le terme « viande » désigne la viande fraîche et conservée, la graisse et le sang.

Article 16 :
L'échange, la cession, l'achat, le troc et la vente sous quelque forme que ce soit de la viande de chasse sur les marches et dans le commerce, ainsi qu'en faveur ou au profit de l'Administration civile ou militaire ou des entreprises agricoles ou industrielles, sont prohibés.

L'autorité administrative détermine les tolérances en faveur des chasseurs traditionnels et des titulaires de permis de chasse locale, à l'intérieur des limites du village, et notamment au profit du chasseur de village, lorsque son métier est consacré par la tradition, et règlemente le transport de viande de chasse.

CHAPITRE VI
DETENTION D'ANIMAUX SAUVAGES EN CAPTIVITE

Article 17 :
L'autorité administrative compétente fixe les tolérances et les modalités de détention par les particuliers, en dehors de tout but commercial, d'un petit nombre d'animaux en captivité obtenus dans des conditions réglementaires ou fortuites.

La détention d'animaux sauvages en captivité par les personnes autres que les détenteurs de permis scientifiques, sont soumise au paiement de taxes annuelles.

CHAPITRE VII
ZONES D'AMENAGEMENTS FAUNIQUES

Article 18 :
Pour l'exécution de l'article 4, alinéa 3, de la présente loi, l'autorité administrative détermine des zones affectées à l'aménagement de la faune et dans lesquelles seront interdits l'exercice de la chasse traditionnelle et de la petite chasse ainsi que la destruction systématique des prédateurs.

Dans ces zones, la chasse et la capture ne seront autorisées qu'aux porteurs de permis spéciaux ou sous le contrôle de l'Administration.

Article 19 :
Le droit de chasse dans les zones d'aménagement faunique pourra faire l'objet de concession en faveur de sociétés de chasse dans le cadre des spécifications des règlements d'aménagement.

CHAPITRE VIII
PROTECTION DES PERSONNES ET DES BIENS
LEGITIME DEFENSE

Article 20 :
Les conditions d'élimination ou d'éloignement des animaux nuisibles aux personnes et aux biens, sont fixées par l'autorité administrative qui détermine les conditions dans lesquelles la chasse sera interdite dans les récoltes pendant ou dans certaines plantations permanentes par mesure de sécurité pour les personnes ou de protection des récoltes.

Article 21 :
Au cas où certains animaux, protégés ou non, constitueraient un danger ou causeraient des dommages, l'autorité administrative peut, par mesure temporaire et exceptionnelle, en assurer ou en autoriser la poursuite ou la destruction après enquête sur place.

Article 22 :
Aucune infraction ne peut être relevée sauf provocation préalable des animaux contre quiconque a fait acte de chasse indûment, mais dans la nécessité immédiate de sa défense ou de celle d'autrui ou de la protection de son bétail domestique ou de sa propre récolte. En cas d'abattage d'un animal intégralement protégé, la preuve de la légitime défense doit être fournie dans les plus brefs délais, aux agents de l'Administration.

CHAPITRE IX
ARMES ET MUNITIONS

Article 23 :
Les armes et les munitions de guerre composant ou ayant composé l'armement réglementaire de forces militaires, de milice ou de police ne peuvent être utilisées pour la chasse.

Article 24 :
L'usage des armes à répétition automatique susceptibles de tirer par rafales est interdit pour la chasse.

Article 25 :
Nul ne peut, sauf exceptions prévues aux articles 26 et 27, obtenir un permis de chasse s'il n'est titulaire d'un permis de port d'arme valant titre de propriété.

Article 26 :
Les guides de chasses agréés, titulaires de la licence prévue à l'article 14, pourront mettre des armes de chasse à la disposition de leurs clients et obtenir pour ces derniers, des permis spéciaux de chasse sportive nonobstant les dispositions de l'article 25.

En cas d'infractions commises par leurs clients, les guides de chasse sont responsables du paiement des amendes qui pourront être prononcées, sauf à eux de prouver qu'ils ont fait tout ce qui était en leur pouvoir pour empêcher l'accomplissement du délit.

Article 27 :
Les enfants mineurs âges de dix-huit ans à vingt-et-un ans et le conjoint d'un titulaire de permis de port d'arme pourront obtenir un permis de chasse sur demande écrite du titulaire justifiant de l'âge et de la parenté du bénéficiaire.

TITRE IV
REPRESSION
CONSTATIONS DES DELITS

Article 28 :
Tout individu trouvé en infraction à la présente loi par un agent habilité mais n'ayant pas qualité d'officier de police judiciaire, sera, s'il ne peut justifier valablement de son identité et de sa résidence, conduit immédiatement devant l'officier de police judiciaire le plus proche ou le cas échéant, devant le Procureur de la République ou le juge de la section de tribunal.

Article 29 :
La procédure de flagrant délit sera applicable en la matière.

ACTIONS ET PROURSUITES

Article 30 :
Les actions et poursuites sont exercées directement par l'autorité administrative devant les juridictions compétentes sans préjudice du droit qui appartient au ministère public près ces juridictions.

PRESCRIPTIONS

Article 31 :
Les délits de chasse se prescrivent par un an, à partir du jour où ces délits ont été constatés.

PRESOMPTIONS DE DELITS

Article 32 :
Est présumé coupable d'infraction à la législation sur la chasse et sera poursuivi dans les mêmes conditions que si l'acte délictueux présumé avait été effectivement constaté quiconque :
1° Est trouvé porteur d'une arme chargée sur les limites d'une réserve intégrale ou d'un parc national ou d'une réserve spéciale de faune ;
2° Est trouvé porteur d'une arme même non chargée, accompagnée de munitions à l'intérieur desdites zones réservées ;
3° Hors d'une propriété close ou d'une agglomération urbaine ou des limites habitées d'un village est trouvé de nuit porteur en même temps d'une arme même non chargée et d'une lampe éblouissante, installée ou non, adaptable au front, à la tête, à la coiffure ou au fusil ;
4° Hors des mêmes lieux et hors d'un terrain de culture portant des récoltes est trouvé porteur d'une arme de chargée, soit en période de fermeture de la chasse, soit de nuit ;
5° En tous temps et en tous lieux, se trouve en possession d'un animal protégé vivant ou mort ou d'une partie de cet animal s'il ne peut faire la preuve par l'exhibition d'un permis réglementaire, ou de toute autre façon, qu'il a été autorisé soit à abattre, soit à détenir ledit animal.

PENALITES

Article 33 :
Les infractions à la présente loi et à ses décrets d'application sont punies :

1° D'une amende de 3.000 francs à 300.000 francs, et d'un emprisonnement de deux mois à un an ou de l'une de ces deux peines seulement ;
2° De la confiscation des animaux blessés ou capturés ou de la dépouille des animaux tués, ou d'une condamnation au paiement d'une somme égale à leur valeur s'ils ne peuvent être commodément saisis.

Ces peines peuvent être assorties en outre :

1° De la confiscation des armes, munitions, engins, matériaux ayant servi à commettre le délit. Le véhicule automobile ou autre, ayant été utilisé délibérément à des fins cynégétiques est considéré comme matériel susceptible de confiscation, notamment lorsqu'il a été utilisé comme moyen de poursuite du gibier, comme engin éblouissant par ses phares ou pour transporter des chasseurs délinquants à l'intérieur d'une réserve ou d'un parc national ;
2° De la déchéance du permis et, éventuellement, de la privation temporaire ou définitive d'octroi de tout autre permis ou licence de chasse ou de capture.

Article 34 :
Les peines d'amende ou d'emprisonnement prévues à l'article précédent sont portées au double lorsque l'une des trois conditions suivantes est réalisée :
1° Délit commis dans une réserve ou parc national ;
2° Délit commis de nuit avec engin éclairant ;
3° Récidive.

Article 35 :
Les peines sont portées au triple lorsque deux des trois circonstances prévues à l'article 34 se trouvent réunies au moment du délit.

Article 36 :
L'emprisonnement sera obligatoire, sans bénéfice du sursis et sans circonstance atténuantes, lorsque l'auteur d'un délit commis dans une réserve ou parc national aura déjà été condamné une première fois pour des faits analogues dans les délais de récidive prévus par la présente loi.

JUGEMENTS ET TRANSACTIONS

Article 37 :
Sauf dans le cas où la peine d'emprisonnement est obligatoire, les infractions à la réglementation de la chasse et de la protection de la faune peuvent faire l'objet d'une transaction entre l'autorité administrative et le délinquant. La transaction peut intervenir avant ou après jugement. Toutefois le jugement devenu définitif, la transaction ne peut porter que sur les condamnations pécuniaires qu'il prononce.

Article 38 :
Il y a récidive en matière de chasse et protection de la faune lorsque, dans les trois ans qui ont précédé l'infraction, le délinquant a déjà été l'objet d'une condamnation définitive ou a bénéficié d'une transaction pour une infraction prévue par la présente loi et ses règlements d'application.

Dans le cas de transaction, l'autorité administrative compétente fournira au tribunal, un exemplaire de l'acte signé par l'intéressé et par l'autorité administrative compétente.

Article 39 :
Les conditions d'application de la présente loi sont déterminées par décret et notamment en ce qui concerne:

— La constitution des réserves, des parcs nationaux et des zones d'aménagement faunique ;
— La représentation de l'Administration devant les tribunaux ;
— La procédure applicable en matière de transaction ;
— Les conditions de délivrance des permis de chasse et de capture, des licences de guide de chasse et les modalités de concession du droit de chasse.

Article 40 :
La présente loi sera exécutée comme loi de l'Etat et publiée au *Journal officiel* de la République de Côte d'Ivoire.

Fait à Abidjan, le 4 Août 1965.

FELIX HOUPHOUET BOIGNY.

ANNEXE I : ESPECES PROTEGEES

CLASSE A

Liste des animaux sauvages intégralement protégés, dont la capture, y compris celles de leurs jeunes ou de leurs œufs, sont interdites sauf aux porteurs de permis scientifiques dans les limites et avec les moyens inscrits au permis.

MAMMIFERES :

Hippopotame nain : *Choeropsis liberiensis*.
Eléphant (jeune accompagnant sa mère et femelle suitée) : *Loxodonta africana*.
Lamantin : *Trichechus senegalensis*.
Chimpanzé : *Pan satryrus verus*.

OISEAUX :

Messager serpentaire : *Sagittarius serpentarius*.
Pintade à poitrine blanche : *Agelastes meleagrides*.

CLASSE B

Liste des animaux sauvages partiellement protégés dits spécifiques dont la chasse et la capture, y compris celles de leurs jeunes ou de leurs œufs, ne sont autorisés qu'aux porteurs de permis de capture dans les limites inscrites aux permis et aux porteurs de permis spéciaux de grande chasse et de chasse touristique de passager mais seulement à titre unitaire comme trophée ou pièce de collection.

MAMMIFERES :

Chevrotain aquatique : *Hyemoschus aquaticus*.
Oryctérope : *Orycteropus afer*.
Colobe magistrat : *Colobus polykomos*.
Céphalophe de Jentink : *Cephalophus jentinki*.
Céphalophe à dos jaune : *Cephalophus sylvivultor*.
Céphalophe zébré : *Cephalophus zebra*.
Pangolin terrestre géant : *Smutsia (syn. Manis) gigantea*.
Pangolin arboricole commun : *Phataginus (syn. Manis) tricuspis*.
Pangolin arboricole à longue queue : *Uromanis (syn.Manis) longicaudata*.
Potamogale : *Potamogale velox*.
Micropotamogale : *Micropotamogale lamottei*.
Néotrague pygmée ou Antilope royale : *Neotragus pygmaeus*.
Hylochère : *Perodicticus potto*.
Galapos : Genre *Galago*.
Anomalures ou Ecureuils volants : Genre *Anomalurus*, *Anomalurops*.

OISEAUX :

Tous les vautours : Famille des Aegypriidés.
Grand Calao d'Abyssinie : *Bucorvus Abyssinicus*.
Marabout : *Leptoptilos crumenirefus*.
Aigrette intermédiaire : *Egretta (syn. Casmerodius) alba*.

Aigrette gazette forme blanche : *Egretta garzetta garzetta*.
Aigrette gazette forme grise : *Egretta garzetta gularis*.
Grue couronnée : *Balearica pavonica*.
Jabiru : *Ephippiorhyncus senegalensis*.
Aigle pêcheur : *Haliaetus (syn. Cuncuma) vocifer*.

CLASSE C

Liste des animaux sauvages partiellement protégés dits cynégétiques dont la chasse des seuls individus adultes est autorisé aux titulaires de permis spéciaux de chasse sportive dans les limites du tableau fixé pour chaque degré et dont la capture y compris celle de leurs jeunes est autorisée aux porteurs de permis de capture dans les limites indiquées aux permis.

MAMMIFERES:

Eléphant : *Loxodonta (syn. Elephas) africana*.
Buffle : *Bubalus (syn. Syncerus) caffer*.
Hippopotame amphibie : *Hippopotamus amphibius*.
Bongo : *Boocercus euryceros*.
Situtonga : *Linnotrgus spokoi*.
Hippotrague : *Hippotragus equinus*.
Cob defassa (Waterbuck) : *Kobus deffa defassa*.
Bubale : *Alcolaphus major*, *Acolaphus lelwel*.
Cob de Buffon : *Adenota kob*.
Léopard ou Panthère d'Afrique : *Panthera pardus*.
Lion : *Leo leo*.

ANNEXE II : OISEAUX SPECTACULAIRES

Liste des oiseaux dits spectaculaires dont la chasse est interdite aux usagers coutumiers et dont l'abattage par les titulaires de permis nationaux de petite chasse et de permis spéciaux sportifs n'est autorisé que dans des limites restreintes à titre de trophées.

OISEAUX :

Hérons, Cigognes et Ibis : parmi les Ardéiformes.
Poules sultanes et Jacanas : parmi les Ralliformes.
Rapaces diurnes (autre que Serpentaire, Vautorus, Aigles pêcheurs qui sont protégés) : tous les Accipitriformes.
Rapaces nocturnes : tous les Strigiformes.
Perroquets : tous les Psittaciformes.
Touracos, Musophages et Coucous : parmi les Cuculiformes.
Couroucou ou Trognon : parmi les Trogoniformes.
Pics et Barbus : parmi les Piciformes.
Martins pêcheurs, Rolliers, Calaos et Guêpiers : parmi les Coraciadiformes.
Merles métallique, Loriot et Souimangas (improprement appelés oiseaux-mouches) : parmi les Passériformes.

ANNEXE III : ESPECES PREDATRICES

Liste des espèces dites prédatrices dont l'abattage est autorisé normalement dans les zones d'habitation et d'exploitation agricole, avicole ou pastorale, dans les conditions prévues pour la chasse coutumière pour les permis de chasse de toutes catégories ainsi que pour la défense des cultures ou du bétail domestique, mais dont la chasse sera réglementée dans les zones affectées à la faune et l'aménagement cynégétique :

CARNASSIERES :

Lycaon ou Cynhyène : *Lycaon Pictus*.
Hyène tachetée : *Crocuta crocuta*.
Chacals : *Canis adustus, Canis aureus*.
Serval et servalin : *Felis serval ; Felis brachyura*.
Chat sauvage : *Felis libyca (sylvestris).*
Chat doré : *Felis aurata*.
Loutres : *Lutra maculicollis, Anyx capensis.*
Ratel : *Mellivora capensis*.
Zorille : *Zorilla striatus*.
Civette : *Civettictis civetta*.
Genettes et Pseudogenette : Genre *Gentta*, genre *Pseudogenetta*.
Nandinie : *Nandina binotata*.
Poïane : *Poïana richardsoni*.
Mangoustes : Genres *Herpostes*, *Ichneumia*.
Mangues et Mungos : Genre *Mungos*.

PRIMATES :

Colobe bai : *Colobus badius*.
Colobe vrai ou de van beneden : *Colobus verus*.
Cynocéphales : Genre *Papio*.
Patas ou singe rouge : *Erythrocebus patas*.
Cercocèbes ou Mangabeys : Genre *Cercocebus*.
Callitriche ou singe vert : *Cercopithecus aethiops*.
Mone : *Cercopithecus mona*.
Hocheur ou pain à cacheter : *Cercopithecus.*
Diane : *Cercopithecus diana*.

REPTILES :

Crocodile du Nil : *Crocodylus nilticus*.
Crocodile à museau de gavial, Cabinda : *Crocodylus oataphractus*.
Crocodile de forêt ou de marais : *Osteolaemus tretrapis*.
Varan du Nil : *Varanus niloticus*.
Varan de savane : *Varanus exanthematicus*.
Python de séba : *Python sebae*.
Python royal : *Python regius*.

ANNEXE IV : PETIT GIBIER

Liste des animaux sauvages dits petit gibier dont la chasse est autorisée pour les usagers coutumiers et pour les titulaires de permis de petite chasse et de permis spéciaux sportifs dans les limites des latitudes générales de chasse autorisées par la loi.

MAMMIFERES :

ANTILOPES :
Guib ou Mina harnaché : *Tragelaphus scriptus*.
Redunca ou Cob des roseaux : *Redunca*.
Céphalophe de Grimm ou biche cochon : *Sylvicapra grimmia*.
Céphalophe (à bande dorsale noir) *: Cephalophus dorsalis*.
Céphalophe de Maxwell ou biche grise : *Philanthomba Maxwelli*.
Céphalophe (à flancs roux ou biche) : *Cephalophus rufilatus*.
Céphalophe noir : *Cephalobus niger*.
Ourébi ou Oribi : *Ourebia ourebi*.

SUIDES :
Phacochère : *Phacochoerus aethiopicus*.
Potamochère : *Potamochoerus porcus*.

DAMANS :
Daman de rocher : *Procavia ruficeps*.
Daman d'arbre : *Dendrohyrax dorsalis*.

RONGEURS :
Lièvre africain, improprement appelé lapin : *Lepus aegypticus.*
Aulacode, improprement appelé agouti : *Aulacodus (syn. Thryonomys), swinderianus*.
Porc-épic : *Hystrix cristata*.
Athérure : *Atherura africana.*
Tous les écureuils : Genre *Xerus*, *Protexerus, Epixerus, Funisciurus, Heliosciurus*.

INSECTIVORES :
Hérisson à ventre blanc : *Atelerix albiventris*.

OISEAUX GIIER :
Oies, canards, sarcelles : Ordre des Ansériformes, famille des Anatidés.
Pintades, francolins, caille, poule de roche : Ordre des Galloformes.
Pluviers, vanneaux, chevaliers, courlis, Oedicnèmes, bécassines : parmi les Chanadriidormes.

REPTLES :
Les tortures.

Décret n° 97-130 du 07 mars 1997
réglementant la détention des ivoires

LE PRESIDENT DE LA REPUBLIQUE,

Sur rapport du Ministre de l'Agriculture et des Ressources Animales, du Ministre du Logement, du Cadre de vie et de l'Environnement, du Ministre de la Culture et du Ministre du Commerce

Vu la constitution

Vu la loi n° 65-255 du 4 août 1965 relative à la protection de la faune et à l'exercice de la chasse telle que modifiée par la loi n° 94-442 du 16 août 1994

Vu la loi n° 87-806 du 28 juillet 1987 portant protection du patrimoine culturel

Vu le décret n° 94-448 du 25 août 1994 portant adhésion de la République de Côte d'Ivoire à la Convention sur le Commerce international des espèces de faune et de flore sauvages menacées de disparition, signée à Washington le 3 mars 1973, ensemble le décret n° 94-449 du 25 août 1994 portant publication de ladite Convention

Vu le décret n° 96-PR/002 du 26 janvier 1996 portant nomination des membres du Gouvernement tel que modifié par le décret n°96-PR/10 du 10 août 1996

Vu le décret n° 96-179 du 1er mars 1996 portant attributions des membres du Gouvernement

Le Conseil des Ministres entendu

DECRETE

CHAPITRE I : DEFINITIONS

Article 1 - Aux termes du présent décret sont des «ivoires»

- les défenses d'éléphant
  - à l'état brut
  - travaillées
  - incorporées à d'autres objets
- les objets en ivoire
- les objets dont l'ivoire est la principale composante en quantité ou en valeur.

Article 2 - Est Collectionneur d'Ivoires au sens du présent décret toute personne, publique ou privées, propriétaire d'un ou plusieurs Ivoires détenus légalement.

Article 3 - Les Ivoires propriété de l'Etat et des collectivités publiques qu'ils soient ou non conservés dans des musées publics nationaux ou régionaux constituent la Collection Nationale des Ivoires.

Article 4 - La Collection Nationale des Ivoires de Côte d'Ivoire et les collections privées d'Ivoires constituent «la Collection Eburnéenne d'Ivoires» ci-après désignée par le sigle « Ivoires CI ».

CHAPITRE II : CONSTITUTION ET NATURE D'IVOIRES CI

Article 5 - Les Ivoires détenus sur le territoire national doivent être déclarés à l'Administration des Eaux et Forêts par le dépôt d'une fiche d'identification conforme à l'annexe au présent décret.

Les certificats d'origine délivrés par l'Administration des Eaux et Forêts en application du décret n° 66-425 susvisé ne dispensent pas leurs possesseurs de la déclaration prévue ci-dessus.

Chaque ivoire déclaré est inscrit sur un registre spécial dit «Répertoire des Ivoires» et doté d'un numéro d'identification.

Un extrait du Répertoire est délivré à chaque Collectionneur pour ce qui le concerne.

L'extrait a valeur de certificat d'origine et constitue une présomption légale de propriété en faveur du Collectionneur qu'il mentionne.

Article 6 - Ivoire-CI fait partie du capital culturel national. A ce titre, les Ivoires qui la composent sont des biens nationaux nonobstant la qualité des personnes dont ils sont la propriété.

CHAPITRE III : DROITS ET OBLIGATIONS DES COLLECTIONNEURS D'IVOIRES

Article 7 - Tout détenteur d'Ivoires a l'obligation d'en faire la déclaration ainsi qu'il est dit à l'article 5 ci-dessus.

Article 8 - Les Ivoires déclarés et répertoriés peuvent faire l'objet :

- d'une exposition permanente ou exceptionnelle
- d'une cession à titre gratuit ou onéreux

Dans ce dernier cas, la mutation au Répertoire des Ivoires est obligatoire et doit être effectuée sans délais.

Article 9 - Tout collectionneur d'Ivoires peut librement en modifier le support ou l'intégrer à un autre objet sous réserve d'en préserver l'intégralité.

La transformation d'Ivoire est subordonnée à une autorisation conjointe du Ministre chargé des Eaux et Forêts et du Ministre chargé de la Culture.

CHAPITRE IV : SORTIE DES IVOIRES DU TERRITOIRE NATIONAL

Article 10 - La sortie des Ivoires du territoire national est interdite sauf autorisation exceptionnelle délivrée par le Ministre chargé des Eaux et Forêts.

Peuvent seules solliciter une telle autorisation à titre strictement personnel, les personnes physiques ayant régulièrement résidé en Côte d'Ivoire pendant une période minimale ininterrompue de six mois.

L'autorisation ne pourra porter que sur des Ivoires enregistrés au Répertoire des Ivoires au nom du demandeur.

CHAPITRE V : INTRODUCTION D'IVOIRES SUR LE TERRITOIRE NATIONAL

Article 11 - L'importation des Ivoires peut être autorisée par le Ministre chargé des Eaux et Forêts au vu d'une autorisation expresse et explicite des autorités compétentes du pays d'origine. Cette autorisation doit être obtenue préalablement à l'embarquement des Ivoires. Elle ne peut être accordée qu'à une personne physique à titre d'objets personnels.
Les Ivoires concernés par l'autorisation doivent être déclarés à leur entrée sur le territoire national faute de quoi ils constituent une marchandise prohibée saisissable à tout moment.

CHAPITRE VI : COMMERCIALISATION DES IVOIRES

Article 12 - Le commerce des Ivoires est interdit.

Toutefois, les commerçants régulièrement installés, disposant d'infrastructures permanentes ouvertes à la clientèle et pratiquant le commerce des Ivoires antérieurement à la date de publication du présent décret pourront continuer cette activité à condition :

- de s'être fait connaitre à l'Administration des Eaux et Forêts
- d'avoir dûment enregistré leurs Ivoires.

Jusqu'à satisfaction de cette double condition, l'exposition de leurs Ivoires ne sera possible que sous réserve d'indiquer de façon apparente : « ces marchandises ne sont pas offertes à la vente ».

CHAPITRE VII : INFRACTIONS ET SANCTIONS

Article 13 - Tout détenteur d'un Ivoire non répertorié est, par application de l'article 32 de la loi n° 65-255 susvisée, présumé coupable d'infraction aux dispositions de cette loi et poursuivi dans les mêmes conditions que si l'acte délictueux avait été effectivement constaté.

Est poursuivi de la même manière l'exposant, même temporaire d'un Ivoire non répertorié.

Article 14 - Sans préjudice des pénalités prévues à l'article 13 ci-dessus, toute transaction ayant pour objet un Ivoire non répertorié, toute tentative d'exportation d'un Ivoire, toute tentative d'importation d'Ivoires en fraude aux dispositions du présent décret est passible des peines prévues par la loi n° 87-806 du 28 juillet 1987 susvisée.

CHAPITRE VIII : MESURES TRANSITOIRES

Article 15 - Les personnes publiques ou privées détentrices d'Ivoires à la date de publication du présent décret disposent d'un délai de deux mois pour en faire déclaration à l'Administration des Eaux et Forêts soit à la Direction de la Protection de la Nature à Abidjan soit au Chef de la Région Forestière du domicile du déclarant.

CHAPITRE IX : DISPOSITIONS FINALES

Article 16 - Le présent décret annule toutes dispositions antérieures contraires.

Article 17 - Le Ministre de l'Agriculture et des Ressources Animales, le Ministre de la Culture, le Ministre du Commerce et le Ministre de l'Economie et des Finances sont chargés, chacun en ce qui le concerne, de l'exécution du présent décret qui sera publié au Journal Officiel de la République de Côte d'Ivoire.

Fait à Abidjan, le 07 mars 1997

Henri Konan BEDIE

Décret n° 66-423 du 15 septembre 1966
fixant le régime des permis de chasse et les modalités de leurs attributions en République de Côte d'Ivoire

LE PRESIDENT DE LA REPUBLIQUE,

Sur proposition du ministre délégué à l'Agriculture,

Vu la loi n° 65-255 du 4 août 1965, relative à la protection de la faune et l'exercice de la chasse et en particulier ses articles 9 et 10 ;

Le Conseil des ministres entendu,

DÉCRÈTE:

Article premier :

Le régime des permis de chasse prévu à l'article 9 de la loi relative à la protection de la faune et à l'exercice de la chasse est fixé comme suit:

TITRE PREMIER
PERMIS DE PETITE CHASSE POUR ARME DE TRAITE

Article 2 :

Le permis de petite chasse pour arme de traite ne peut être délivré qu'à des personnes âgées d'au moins vingt et un ans, titulaires d'un permis de port d'arme de traite.

La durée de validité est limitée à la saison de chasse en cours. Il est délivré par les sous-préfets qui peuvent déléguer leurs pouvoirs aux représentants compétents du ministère de l'Agriculture.

Il donne à son titulaire, dans le cadre de la réglementation de la chasse, le droit de tirer uniquement dans la sous-préfecture de sa résidence, les animaux cités aux annexes III et IV de la loi sur la protection de la faune et l'exercice de la chasse.

Cette chasse aux armes de traite sera interdite dans les zones d'aménagement faunique prévues à l'article 19 de la loi susvisée.

PERMIS NATIONAL

Article 3 :

Il ne peut être délivré qu'à des personnes âgées d'au moins vingt et un ans, titulaires d'un permis de port d'armes perfectionnées.

Sa durée de validité est limitée à la saison de chasse en cours. Il est délivré par le sous-préfet qui peut déléguer ses pouvoirs au représentant compétent du ministère de l'Agriculture.

Dans les communes de plein exercice, les permis nationaux sont délivrés par le préfet qui peut également déléguer ses pouvoirs au représentant compétent du ministère de l'agriculture.

Tout permis national donne le droit de tirer sur l'ensemble du territoire les animaux des annexes II, III, IV dans les limites prévues à ces annexes.

TITRE II
PERMIS DE CHASSE SPORTIVE
PERMIS DE MOYENNE CHASSE

Article 4 :

Il ne peut être délivré qu'à des personnes âgées d'au moins vingt et un ans, titulaires d'un permis de port d'armes perfectionnées, rayées d'un calibre égal ou supérieur à 7 millimètres.

La durée de validité est limitée à saison de chasse en cours. Il est délivré par le directeur des services compétents du ministère de l'Agriculture.

Il donne à son titulaire dans le cadre de la réglementation sur la chasse, le droit de tirer sur l'ensemble du territoire, excepté dans les zones d'aménagement faunique ;
1° Les animaux des annexes II., III et IV dans les limites prévues à ces annexes sans taxes d'abatage ;
2° les animaux partiellement protégés ;

Annexe I, classe C dans les limites indiquées ci-dessous et moyennant paiement, après abattage, d'une taxe dont le montant par animal abattu est fixé par arrêté du ministre délégué à l'agriculture pris sur avis du ministre délégué aux affaires économiques et financières.

Espèces :	Nombre total d'animaux dont l'abattage est autorisé par permis de moyenne de chasse
Eléphant	1
Buffle	1
Hippopotame	1
Hippotrague	1
Cob de Buffon	1
Cob defassa	1
Bubale	1
Léopard	1
Lion	1

PERMIS DE PASSAGER

Article 5 :

Il ne peut être délivré qu'à des personnes âgées d'au moins 21 ans, titulaire d'un permis de port d'arme rayée d'un calibre supérieur à 7 millimètres ou pouvant justifier de la location d'une arme rayée conformément à la réglementation en vigueur.

Il est délivré par le directeur des services compétents du ministère de l'agriculture et valable un mois.

Il donne à son titulaire, dans le cadre de la réglementation de la chasse, le droit de tirer sur l'ensemble du territoire :

1° Les animaux cités aux annexes II, III, IV dans les limites prévues à ces annexes sans taxe d'abattage ;

2° Les animaux partiellement protégés annexe I, classe C, dans les limites indiquées ci-dessous et moyennant paiement après abattage d'une taxe dont le montant par animal abattu est fixé par arrêté du ministre délégué à l'Agriculture pris sur avis du ministre délégué aux Affaires Economiques et Financières.

Espèces :	*Nombre total d'animaux dont l'abattage est autorisé par permis de passager*
Eléphant…………………………………………………………	1
Buffle…………………………………………………………….	1
Hippopotame…………………………………………………………….	1
Bongo…………………………………………………………….	1
Situtunga…………………………………………………………….	1
Hippotrague…………………………………………………………..	1
Cob defassa……………………………………………………………......	1
Cob de Buffon………………………………………………………...	1
Bubale………………………………………………………………	1
Léopard ou panthère…………………………………………………..	1
Lion…………………………………………………………………………	1

3° Les animaux partiellement protégés, annexe I, classe B, à titre unitaire et sans taxe d'abattage excepté pour le colobe magistrat, le céphalophe à dos jaune et l'hylochère.

PERMIS DE GRANDE CHASSE

Article 6 :

Il ne peut être délivré qu'à des personnes âgées d'au moins 21 ans, titulaires d'un permis de port d'armes rayées d'un calibre supérieur à 7 millimètres.

Sa durée de validité est limitée à la saison de chasse en en cours. Il est délivré par le directeur des services compétents du ministère de l'agriculture.

Il donne à son titulaire, dans le cadre de la réglementation de la chasse, le droit de tirer sur l'ensemble du territoire :

1° Les animaux cités aux annexes II, III, IV dans les limites prévues à ces annexes sans taxe d'abattage ;

2° Les animaux partiellement protégés, annexe I, classe C, dans les limites indiqués ci-dessous et moyennant paiement après abattage d'une taxe dont le montant par animal abattu est fixé par arrêté du ministre délégué à l'Agriculture pris sur avis du ministre délégué aux Affaires économiques et financières.

Nombre total d'animaux dont l'abattage est autorisé
par permis de grande chasse

Espèces :

Eléphant…………………………………………………………………	2
Buffle…………………………………………………………………	2
Hippopotame………………………………………………………………………	1
Bongo…………………………………………………………………	2
Situtunga………………………………………………………………………	2
Hippotrague…………………………………………………………………..	2
Cob defassa…………………………………………………………………......	2
Bubale…………………………………………………………………………	2
Cob de Buffon……………………………………………………………...	3
Léopard ou panthère………………………………………………………..	2
Lion…………………………………………………………………………	1

3° Les animaux partiellement protégés, annexe I, classe B, à titre unitaire sans taxe d'abattage excepté pour :

- Colobe magistrat ;
- Céphalophe à dos jaune ;
- Hylochère.

TITRE III
PERMIS SPECIAL DE CAPTURE COMMERCIALE

Article 7 :

Il ne être délivré qu'à une personne ou une société présentant du point de vue technique, toutes les garanties jugées nécessaires et suffisantes pour se livrer à ce genre d'activité et titulaire d'une patente commerciale.

Il est accordé par le ministre délégué à l'Agriculture et est valable pour l'année en cours. Mention y est portée des moyens de capture autorisés.

En ce qui concerne les animaux intégralement protégés, les permis de capture commerciale ne pourront être accordés qu'à des titulaires de permis scientifiques de capture dans les conditions prévues à l'article 8.

Ces permis précisent exactement les droits conférés à son détenteur et le périmètre dans lequel ils peuvent s'exercer.

Les titulaires de permis de capture commerciale d'animaux intégralement et partiellement protégés sont obligés de tenir un carnet de capture qui sera présenté de même que le permis, à toute réquisition des agents habilités à cet effet et dans lesquels seront enregistrés au jour le jour, les animaux protégés qu'ils auront capturés, incidemment abattus ou acquis dans les limites autorisées par leur permis.

Mention sera porté sur ce carnet de l'espèce de l'animal capturé, de son sexe, des caractéristiques permettant son identification, des circonstances de la capture, notamment la date et la localité de la délivrance du certificat d'origine puis, en cas d'exportation, de la date et du point de sortie, du visa sanitaire, du visa de service compétent du ministère de l'Agriculture contrôlant l'exportation des animaux protégés et du visa de la Douane constatant la sortie.

En cas de vente sur place ou de don d'un animal protégé à un parc zoologique du gouvernement, la cession ou le don seront mentionnés sur le carnet de capture au lieu et place de l'exportation.

Le titulaire du permis de capture commerciale sera autorisé à détenir jusqu'à leur vente ou leur exportation, les animaux régulièrement acquis durant la validité du permis et dûment inscrits au carnet de capture s'il s'agit d'animaux protégés.

Ces animaux devront être maintenus en bon état de soin et d'hygiène.

Les permis de capture ne donnent aucun des droits équivalents à un permis de chasse et ne permettent pas l'utilisation d'armes à feu.

En plus de la patente commerciale et du permis de capture commerciale, le bénéficiaire pourra être astreint au paiement de droits et de taxes fixés annuellement.

TITRE IV
PERMIS SCIENTIFIQUE DE CHASSE OU DE CAPTURE

Article 8 :

Ils sont délivrés par le ministre délégué à l'Agriculture.

La demande de permis doit indiquer le nom et la qualité du bénéficiaire et du titulaire, les motifs invoqués, le nombre d'animaux de chaque espèce intégralement protégés dont l'abattage ou la capture est sollicitée.

Le permis précise exactement la durée de validité, les droits conférés à son détenteur et le périmètre dans lequel il peut s'exercer.

TITRE V
OBLGATIONS DES TITULAIRES DE PERMIS

Article 9 :

Les permis sont essentiellement personnels ; ils ne peuvent être ni cédés, ni vendus.

Il ne peut être délivré à la même personne qu'un seul permis dans la même année. Cependant, il peut être accordé, pendant la période de validité d'un permis, un permis d'une catégorie supérieure, moyennant le versement de la différence de prix entre les deux permis. Le total des latitudes d'abattage ainsi accordé ne pourra jamais dépasser le total de celles prévues par le permis de la catégorie la plus élevée.

Toute personne désirant obtenir un permis doit adresser au sous-préfet du lieu de sa résidence ou au préfet, dans le cas prévu à l'article 3, une demande indiquant ou comprenant :

1° Son nom, prénom, date et lieu de naissance, nationalité, domicile et profession ;

2° Nature du permis ;

3° Une déclaration certifiant que l'intéressé n'a jamais été condamné pour délit de chasse ou indiquant, s'il y a lieu, la date, le lieu et la nature de la condamnation prononcée.

A cette demande doivent être joints :

1° Le récépissé du droit afférent au permis demandé ;
2° deux photographies types « photo d'identité » ;
3° Le permis de port d'armes du demandeur ;
4° S'il y a lieu, le précédent permis obtenu.

Le sous-préfet statue en ce qui concerne les permis de chasse pour armes de traite et de petite chasse et transmet les autres demandes avec avis à l'autorité qualifiée.

Aucun permis ne peut être délivré sans que la demande ait été déposée, instruite, visé et transmise dans ces conditions.

Leur délivrance peut être refusée par l'Autorité administrative compétente.

En cas de perte du permis, une déclaration doit être faite par l'intéressé, à la sous-préfecture de son lieu habituel de résidence ou à la préfecture pour le cas visé à l'article 3. Un duplicatum pourra être délivré moyennant le versement d'une taxe spéciale fixée au dixième du droit afférent prévu pour le permis correspondant.

REDEVANCE

Article 10 :

Les redevances prévues à l'occasion de la délivrance des permis et des duplicata et les taxes d'abattage sont fixées par arrêté du ministre délégué à l'Agriculture pris sur avis du ministre délégué aux Affaires économiques et financières.

PUBLICITE DES PERMIS

Article 11 :

La publication des permis scientifiques et des permis de capture commerciale, sera fait au *Journal officiel*, avec indication des noms et qualités des titulaires des permis, de la nature et de la validité de ceux-ci.

DECHEANCE DES PERMIS

Article 12 :

La publication de la déchéance ou de la privation d'octroi de permis de chasse ou des permis de capture sera faite au *Journal officiel*, avec indication des noms et qualités des titulaires des permis, de la nature et de la validité de ceux-ci.

De plus, quiconque aura obtenu un permis de chasse en trompant la bonne foi de l'autorité administrative, bien qu'il ait été déchu de ses droits et qui sera convaincu de fraude, verra le nouveau permis confisqué et, s'il a chassé sous son couvert, sera considéré comme à nouveau en contravention avec des dispositions du présent décret.

CARNET DE CHASSE

Article 13 :

Les titulaires d'un permis quelconque autre que le permis pour arme de traite et le permis de petite chasse national sont obligés de tenir un carnet de chasse qui sera présenté de même que le permis, à toute réquisition des agents de l'autorité et où seront enregistrés au jour le jour, les animaux qu'ils auront abattus dans les limites autorisées par les latitudes d'abattage afférentes à chaque sorte de permis.

Pour chaque animal seront précisés :
- la date et le lieu de l'abattage (ou de la capture) et le sexe ;
- pour les éléphants, le poids, la longueur de la courbe externe, la circonférence de base de chaque pointe.

Tout animal capturé vivant par suite de circonstance fortuites compte, quelque soit son âge, pour un animal tué.

Les taxes d'abattage doivent être payées dans les délais les plus brefs, dans la limite de quinze jours francs après l'abattage, sous peine de confiscation des trophées et dépouilles et de l'arme ayant servi à l'abattage.

Les taxes seront payées sur présentation du permis de chasse à la sous-préfecture du lieu d'abattage.

En cas de force majeure, le paiement pourra avoir lieu à la sous-préfecture de la résidence du chasseur. Ce dernier devra alors informer les autorités de la sous-préfecture où a eu lieu l'abattage, du règlement de la taxe en indiquant le numéro, la date et le montant du récépissé.

Le montant de la perception, le numéro, la date et le lieu de délivrance de la quittance doivent être mentionnés sur le carnet de la chasse par l'agent percepteur.

ANIMAUX BLESSES

Article 14 :

Toute personne qui a blessé un animal est tenue de tout mettre en œuvre pour le retrouver et l'achever, même s'il s'agit d'un animal qu'elle n'avait pas l'autorisation de chasser, à l'exception toutefois de la poursuite dans une réserve de faune où il se serait réfugié. Elle doit, dans ce dernier cas, faire une déclaration circonstanciée de l'autorité chargée de la gestion de la réserve.

L'animal blessé doit être inscrit le jour même sur le carnet de chasse avec la mention « blessé ». Si deux animaux de la même espèce ont été blessés, la latitude d'abattage de l'espèce est diminuée d'une unité.

Si l'animal blessé est un éléphant, un buffle, un lion ou un léopard et qu'il n'a pas été trouvé, ni tué dans un délai de vingt-quatre heures après, le moment où il a été blessé, déclaration circonstanciée doit être faite immédiatement à l'autorité administrative la plus proche.

TITRE VI
INFRACTIONS ET PENALITES

Article 15 :

Les infractions aux dispositions du présent décret sont recherchées, constatées, réprimées et réparées conformément aux règles générales en vigueur et prévues par la loi sur la protection de la faune et l'exercice de la chasse.

Article 16 :

Le ministre délégué à l'Agriculture, le ministre des Forces armées et du Service civique et le ministre de l'Intérieur sont chargés, chacun en ce qui le concerne, de l'application du présent décret qui sera publié au *Journal officiel* de la République.

Fait à Abidjan, le 15 septembre 1966.

FELIX HOUPHOUËT-BOIGNY.

Décret n° 66-424 du 15 septembre 1966
relatif à la licence de guide de chasse

LE PRESIDENT DE LA REPUBLIQUE,

Sur proposition du ministre délégué à l'Agriculture,

Vu la loi n° 65-255 du 4 août 1965, relative à la protection de la faune et l'exercice de la chasse, en particulier ses 13 et 14 ;

Le Conseil des ministres entendu,

DÉCRÈTE:

Article premier :

Fait acte de guide de chasse quiconque loue ses propres services, directement ou par l'intermédiaire d'un employeur, à titre principal ou accessoire, pour conduire ou accompagner une expédition de chasse afin de faire profiter autrui de ses connaissances cynégétiques et le protéger contre les dangers qu'il pourrait encourir.

Article 2 :

Nul ne peut faire acte de guide de chasse sur le territoire de la République de Côte d'Ivoire, que ce soit de manière habituelle ou occasionnelle, s'il n'est titulaire de la licence spéciale correspondante.

Article 3 :

Peuvent seuls se porter candidats à l'obtention de la licence de guide de chasse, les personnes remplissant les conditions suivantes :
- Etre citoyen de la Côte d'Ivoire ou, pour les étrangers, résidé depuis au moins trios ans en Côte d'Ivoire ou dans un Etat voisin ;
- Etre âgé d'au moins vingt et un ans révolus ;
- N'avoir encouru aucune peine pouvant entraîner la perte des droits civiques.

Les demandes d'inscription, accompagnées des pièces justificatives correspondantes et indiquant les noms, prénoms, profession, nationalité et domicile des candidats, doivent parvenir avant le 1er décembre de chaque année au ministère de l'Agriculture.

Ce dernier dresse par arrêté prenant effet au 1er janvier, la liste des candidats inscrits.

Article 4 :

Tout candidat doit effectuer une période d'apprentissage durant une saison de chasse à compter de la date de son inscription.

Pendant cette période, nonobstant les dispositions de l'article 2 ci-dessus et sous réserve que déclaration préalable en soit faite aux chefs des inspections forestières intéressés, le candidat peut accompagner des expéditions de chasse en qualité d'apprenti, sous la responsabilité et en compagnie d'un guide de chasse licencié. La présence de ce dernier n'étant toutefois obligatoire à ses côtés que pour la recherche et la poursuite des animaux suivants : éléphants, buffles, lions, léopards. Les déclarations mentionnées ci-dessus doivent être visée par le guide de chasse employeur lequel est également tenu, après chaque expédition, de délivrer un certificat circonstanciel à son apprenti.

Pendant la même période, le candidat peut également, sur sa demande, se voir éventuellement confier dans le cadre de son apprentissage, l'exécution de chasses de destruction ou de divers travaux d'ordre cynégétique sous la direction et le contrôle du service compétent du ministère de l'Agriculture.

Article 5 :

Une fois achevée sa période d'apprentissage, chaque candidat doit subir un examen devant une commission composée comme suit :

Président :
Le ministre délégué à l'Agriculture ou son représentant.

Membres :

- Le directeur des services compétents du ministère de l'Agriculture ou son représentant ;
- Le directeur de l'Office du Tourisme ou son représentant ;
- Un guide de chasse licencié ou à défaut, un représentant d'une profession ayant des activités en rapport avec le tourisme cynégétique.

Cette commission se réunit sur convocation de son Président. Elle peut, à la diligence de son président faire appel au concours d'examinateurs pris en dehors d'elle ; ceux-ci n'ont que voix consultative.

Lors des délibérations et en cas de partage égal de voix, celle du président est prépondérante.

Article 6 :

L'examen comprend une épreuve théorique, une épreuve pratique et une appréciation des activités du candidat pendant sa période d'apprentissage.

a) Matières obligatoires	Coefficient
- Notions simples de zoologie, écologie des animaux sauvages, cynégétique	2
- Réglementation sur la chasse et la protection de la faune	3
- Epreuve de tir	3
- Armes et munitions, réglementation en la matière	1
- Géographie des régions de chasse	1
- Hygiène, prophylaxie, soins courants et d'urgence	1

b) Matières facultatives	Coefficient
- Langues étrangères (anglais, allemand, espagnol)	2
- Langues vernaculaires véhiculaires	2

L'épreuve pratique comprend :	Coefficient
- Dépannage d'un véhicule	2
- Tir sur cible	1
- L'appréciation des activités du candidat pendant sa période d'apprentissage est faite au vu de ses carnets de chasse des certificats mentionnés à l'article 4 ci-dessus et des rapports techniques rédigés par les services compétents du ministère de l'agriculture et visés par l'Office du tourisme	4

Article 7 :

Chaque matière examinée donne lieu à l'attribution d'une note chiffrée comprise entre 0 et 10.Toute note inférieure à 3 attribuée dans une matière obligatoire est éliminatoire. Le nombre minimum de points que doit totaliser un candidat pour obtenir la licence de guide de chasse est de 105.

Les résultats des épreuves sont consignés dans un procès-verbal. Tous les candidats qui ne sont pas susceptibles d'obtenir la licence de guide de chasse, peuvent être autorisés à prolonger leur apprentissage durant une nouvelle saison de chasse.

Article 8 :

La licence de guide de chasse est accordée par l'arrêté du ministre délégué à l'Agriculture. Cette licence est définitive, sauf sanction disciplinaire de retrait prise en application de l'article 15 ci-dessous.

Article 9 :

Nonobstant les dispositions des articles précédents et à titre transitoire, le ministre délégué à l'Agriculture pourra accorder la licence de guide de chasse aux personnes ayant une solide expérience cynégétique et ayant déjà exercé ce métier en Côte d'Ivoire antérieurement à la publication du présent décret.

Les candidats ne remplissant que la première de ces conditions seront soumis à l'examen mais pourront être dispensés de l'apprentissage prévu à l'article 4.

Il sera mis fin à cette période transitoire par l'arrêté du ministre délégué à l'Agriculture.

Article 10 :

Les guides chasse ont la stricte obligation de :

- Faire observer par leurs clients, la réglementation en vigueur en matière de chasse et de protection de la faune ;
- Protéger leurs clients contre tous risques inhérents à la chasse ;
- Achever les animaux blessés ;
- Hors des deux cas mentionnés ci-dessus, ne tirer eux-mêmes qu'avec le consentement exprès de leurs clients ;
- Conserver en toutes circonstances à la chasse son caractère sportif ;
- Avoir toujours une conduite et une tenue correctes à l'égard des clients, du personnel employé et des populations rencontrées.

Article 11 :

Tout guide de chasse est tenu de contracter auprès d'une compagnie d'assurance agréée, préalablement à toute expédition de chasse, une assurance couvrant intégralement sa responsabilité civile et celle des apprentis-guides et du personnel qu'il emploie pour tout accident ou dommage qui pourrait survenir à ses clients ou des tiers au cours de l'expédition.

En cas d'accident concernant un client ou un tiers du fait d'un client, le guide de chasse doit aviser immédiatement l'Autorité administrative la plus proche qui procède aussitôt à une enquête.

Article 12 :

Les guides de chasse doivent déclarer chaque expédition qu'ils sont appelés à conduire ou à accompagner au chef de l'Inspection forestière du lieu de départ initial ou du point d'entrée en Côte d'Ivoire de l'expédition.

Sauf en cas de force majeure dont la preuve incombe au guide intéressé cette déclaration doit parvenir à destination quinze jours au moins avant la date prévue pour le commencement de l'expédition. Elle précise, outre les noms du guide et de ses clients, les dates et lieux prévus pour le début et la fin de l'expédition ou, éventuellement, son entrée et sa sortie du territoire ivoirien.

Article 13 :

Les guides de chasse ne peuvent conduire ou accompagner une expédition de chasse sans être munis de permis de grande chasse.

Il est toutefois interdit d'utiliser ces permis pour accroître de quelque manière que ce soit les latitudes d'abattage de leurs clients et qu'un guide est amené à achever doit obligatoirement être inscrit au compte et sur le carnet de chasse du client.

Article 14 :

Les guides de chasse sont responsables civilement des infractions à la réglementation de la protection de la faune et de l'exercice de la chasse commises par leurs clients au cours des expéditions de chasse qu'ils conduisent ou accompagnent.

Ils peuvent toutefois s'affranchir de cette responsabilité civile dans les conditions prévues par l'article 26 de la loi réglementant la protection de la faune et l'exercice de la chasse.

Les guides de chasse demeurent toujours et en toutes circonstances responsables du paiement en Côte d'Ivoire des taxes d'abattage pouvant être dues pour les animaux tués par leurs clients sur le territoire de la République.

Article 15 :

Indépendamment des responsabilités ainsi que des poursuites et sanctions judiciaires qu'ils peuvent éventuellement encourir, les guides de chasse sont passibles de sanctions disciplinaires pour infractions aux règlements en vigueur en matière de chasse et de protection de la faune, négligence ou inobservation des règles éditées aux articles 9, 10, 11 et 12 ci-dessus, délit de droit commun ou incompétence notoire.

Ces sanctions sont, par ordre de gravité croissante :

- L'avertissement ;
- Le blâme ;
- Le retrait de la licence pour une durée déterminée ;
- Le retrait définitif de la licence.

Elles sont prononcées par arrêté du ministre délégué à l'Agriculture les deux premières directement, les autres après consultation et sur proposition de la commission prévue à l'article 5.

Cette commission établit ses propositions au vu des documents qui lui sont soumis et des explications ou justifications que le guide mis en cause doit avoir obligatoirement été invité à donner par écrit sur les faits qui lui sont reprochés.

Article 16 :

Les infractions aux dispositions du présent décret sont réprimées conformément à la loi n° 65-255 du 4 août 1965.

Article 17 :

Sous réserve d'accord de réciprocité, les licences de guide de chasse délivrées dans les Etats voisins pourront être valables en République de Côte d'Ivoire.

Article 18 :

Le ministre délégué à l'Agriculture, le ministre de l'Intérieur, le ministre des Forces armées et du Service civique et le ministre chargé du Tourisme sont chargés, chacun en ce qui le concerne, de l'application du présent décret qui sera publié au *Journal officiel* de la République de Côte d'Ivoire.

Fait à Abidjan, le 15 septembre 1966.

FELIX HOUPHOUËT-BOIGNY.

Décret n° 66-425 du 15 septembre 1966
réglementant le trafic, la circulation, l'importation, l'exploitation des trophées d'animaux protégés et spectaculaires et de leurs dépouilles

LE PRESIDENT DE LA REPUBLIQUE,

Sur proposition du ministre délégué à l'Agriculture,

Vu la loi n° 65-255 du 4 août 1965, relative à la protection de la faune et l'exercice de la chasse et en particulier son articles 15 ;

Le conseil des ministres entendu,

DÉCRÈTE:

DEPOUILLES ET TROPHEES

Article premier :

Les titulaires des permis de chasse donnant droit à l'abattage d'animaux mentionnés aux annexes I et II de la loi sur la protection de la faune et l'exercice de la chasse ainsi que les titulaires de permis spéciaux de capture et scientifiques peuvent librement disposer des dépouilles d'animaux régulièrement abattus ou capturés par eux.

La circulation des dépouilles d'animaux intégralement protégés (sauf pour les titulaires d'un permis scientifique mentionnant d'une façon précise ces animaux), est strictement prohibée et donne lieu à saisie.

CERTIFICAT D'ORIGINE

Article 2 :

Aucun animal partiellement protégé mort ou vif, aucun trophée ou dépouille de ces animaux ne peut être détenu, circuler ou être exporté de Côte d'Ivoire sans être accompagné d'un certificat d'origine permettant son indentification.

Les certificats sont délivrés par les services compétents du ministère de l'Agriculture ou à défaut, par les chefs de circonscriptions administratives.

Les pointes d'éléphant devront porter des mentions indélébiles :

a) Le numéro de permis suivi des lettres A ou B pour différentier les pointes, suivi des deux derniers chiffres de l'année de délivrance du permis (ex. : 37 A 65) ;

b) Le poids de la pointe.

Ces mentions ainsi que les courbes externes et la circonférence à la base des pointes devront figurer sur le certificat d'origine et sur le carnet de chasse.

Les certificats d'origines devront mentionner les numéros, date, montant et lieu de versement des taxes d'abattage lorsqu'ils sont prévus. Une ampliation de chaque certificat d'origine devra obligatoirement être adressée aux services compétents du ministère de l'Agriculture.

En ce qui concerne les animaux vivants, trophées ou dépouilles provenant d'un territoire sera délivré par le poste administratif ou par le poste de Douane frontière ivoirien, sur production d'une pièce émanant des autorités étrangères et justifiant la légitimité de la possession des animaux, trophées ou dépouilles. Mention de cette pièce sera faite sur le certificat d'origine.

MASSACRES TROUVES

Article 3 :

Il est interdit de s'approprier :
- L'ivoire des éléphants trouvés morts ;
- Les massacres et trophées des animaux protégés prouvés morts.

Ces dépouilles doivent être remises au premier poste administratif atteint.

Elles sont expédiées au receveur des Domaines qui, après publicité, procède à la vente aux enchères publique au profit du budget de l'Etat.

Article 4 :

Les personnes qui remettront à l'autorité administrative des défenses d'éléphants trouvées, percevront une prime par kilogramme dont le montant sera fixé par arrêté du ministre délégué à l'Agriculture, pris sur avis du ministre délégué aux Affaires économiques et financières.

Un procès-verbal sera immédiatement dressé par l'autorité administrative qui recevra l'ivoire en dépôt. Ce document indiquera le nom du déposant ainsi que la date, le lieu et les circonstances de la trouvaille, de façon aussi précise que possible, le poids et la longueur de chaque défense ainsi que le numéro d'immatriculation au registre de dépôt d'ivoire de la circonscription administrative.

Un exemplaire de ce procès-verbal sera toujours adressé, dans les meilleurs délais, au service local compétent du ministère de l'Agriculture.

DEPOUILLES SAISIES

Article 5 :

Sont expédiés au receveur des Domaines et vendu aux enchères publiques, l'ivoire et les dépouilles provenant de la confiscation ou saisie pour infraction à la réglementation sur la chasse.

Le receveur des Domaines dresse au préfet un extrait du procès-verbal de vente aux enchères publiques.

Article 6 :

Les infractions aux dispositions du présent décret sont recherchées constatées, réprimées et réparées conformément aux règles générales en vigueur et prévues par la loi sur la protection de la faune et l'exercice de la chasse.

Article 7 :

Le ministre délégué à l'Agriculture, le ministre des Forces armées et du Service civique et le ministre de l'Intérieur sont chargés, chacun en ce qui concerne, de l'exécution du présent décret qui sera publié au *Journal officiel* de la République.

Fait à Abidjan, le 15 septembre 1966.

FELIX HOUPHOUËT-BOIGNY.

Décret n° 66-433 du 15 septembre 1966
portant statut et réglementation de la procédure de classement des réserves naturelles, intégrales ou partielles et des parcs nationaux

LE PRESIDENT DE LA REPUBLIQUE,

Sur le rapport du ministre délégué à l'Agriculture,

Vu la loi n° 65-255 du 4 août 1965, relative à la protection de la faune et l'exercice de la chasse en particulier en ses articles 4 et 5 ;

Vu la loi n° 65-425 du 20 décembre 1965, portant Code forestier ;

Le conseil des ministres entendu,

DÉCRÈTE:

Article premier :

Les réserves naturelles intégrales font partie du domaine forestier classé de la République de Côte d'Ivoire.

Sont strictement interdits sur toute leur étendue :

- Toute exploitation forestière agricole ou minière ;
- Toutes fouilles ou prospections, sondages terrassements ou constructions et généralement tous travaux tendant à modifier l'aspect du terrain ou de la faune végétation ;
- Tout acte de nature à nuire ou à apporter des perturbations à la faune ou à la flore.

Les réserves naturelles intégrales sont affranchies de tout droit d'usage.

La pénétration, la circulation y compris par voie aérienne à des altitudes inférieures à 200 mètres, le campement dans les réserves naturelles intégrales sont strictement interdits, sauf aux personnes suivantes ;

1° Sans autorisation spéciale, les fonctionnaires et agents des services compétents du ministère de l'Agriculture spécialement chargés de la conservation de ces réserves et le personnel placé sous leurs ordres et les accompagnant. Ces fonctionnaires peuvent faire usage d'armes à feu pour leur légitime défense et celle des personnes qu'ils escortent ;

2° Sur autorisation écrite délivrée par le ministre délégué à l'Agriculture ou son délégué, des personnalités scientifiques dans des buts strictement scientifiques et sous escorte désignée dans l'autorisation.

L'autorisation pourra prévoir des récoltes d'échantillons minéraux ne devant pas modifier apparemment les lieux ;

- Les récoltes d'échantillons botaniques devront se limiter aux organes nécessaires à l'identification des espèces ;
- Les captures d'animaux ne pourront en aucun cas donner lieu à usage d'armes à feu.

RESERVES NATURELLES PARTIELLES

Article 2 :

En vue de la conservation de la nature, certaines zones pourront faire l'objet de restrictions quant à la chasse, la nature des animaux, l'exploitation des végétaux ou l'installation des bâtiments.

Les réserves partielles comprennent :

- Les réserves à caractères scientifiques, telles que les réserves botaniques, zoologiques ou paléontologiques ;
- Les réserves à caractères touristiques ou climatiques ;
- Les sources naturelles d'énergie hydro-électrique.

Ces réserves font partie du domaine forestier classé.

PARCS NATIONAUX

Article 3 :

Les parcs nationaux font partie du domaine forestier classé. Ils sont consacrés à la propagation, à la protection de la vie animale et de la végétation sauvage, à la conservation d'objets d'intérêt esthétique, géologique, historique ou scientifique au profit du public, pour sont éducation et sa récréation.

La direction, la gestion et la surveillance en sont confies aux services compétents du ministère de l'Agriculture.

Les parcs nationaux sont affranchis de tout droit d'usage. Y sont strictement prohibés, la chasse, la pêche, ou la capture de tous animaux, le prélèvement d'espèces végétales ou d'objets quelconques, l'exploitation ou la prospection minière.

Les règles éditées à l'article premier précédent s'applique à la pénétration, à la circulation y compris par voie aérienne et aux recherches scientifiques dans les parcs nationaux.

Toutefois les parcs nationaux pourront en outre être ouverts au public aux conditions suivantes :

- Contrôle effectif des entrées et des sorties ;
- Circulation en véhicule limitée aux routes et aux pistes ouvertes au public ;
- Circulation à pied, chasse photographique et cinématographique uniquement sous escorte et limitées à certains secteurs ;
- Circulation de nuit par quelque moyen que ce soit interdite sauf sur certaines routes d'intérêt général ;
- Stationnement de jour aux emplacements indiqués par le personnel de surveillance ;
- Campement de nuit aux emplacements réservés à cet effet ;
- Interdiction de port d'armes. Celles dont les visiteurs se trouveraient éventuellement munis doivent, avant l'entrée dans le parc être démontées et mises dans les étuis. Déclaration devra en être faite au poste de contrôle et le surveillant pourra y apposer les scellés.

Pour chaque parc national, un règlement intérieur précisera les modalités d'application du présent article.

Dans les parcs nationaux, les services compétents du ministère de l'Agriculture pourront entreprendre tous les travaux et aménagements nécessaires à leur équipement dans des buts scientifiques, éducatifs et touristiques.

Les personnes désirant visiter un parc national doivent être munies d'un permis de visite délivré par les services compétents du ministère de l'Agriculture.

RESERVE DE FAUNE

Article 4 :

Dans les réserves zoologiques « Réserves de faune », tout acte de chasse est strictement interdit sauf le cas de légitime défense ou de protection des personnes et des biens.

Dans les réserves de faune demeure libre l'exercice du droit de pêche, de pâturage, de passage de récolte de miel, de la cire et des fruits sauvages pour les ayants droit.

S'il y a lieu, pour chaque réserve de faune, un règlement intérieur précise les conditions de pénétration, de circulation et de campement.

La circulation aérienne à une altitude inférieure à 200 mètres est toujours interdite.

Dans les réserves de faune aménagées, un permis de visite délivré par les services compétents et le paiement d'une redevance pourra être exigé.

PROCEDURE DE CLASSEMENT DES RESERVES NATURELLES INTEGRALES ET PARTIELLES ET DES PARCS NATIONAUX

Article 5 :

Les procédures de classement des réserves naturelles, intégrales et partielles et des parcs nationaux sont fixées comme suit :

Les avant-projets émanant soit des préfets ou sous-préfets, soit des maires, soit des services techniques du ministère de l'Agriculture doivent être transmis avant toute autre procédure au ministère délégué à l'Agriculture.

Chaque projet doit fournir les précisions suivantes concernant les réserves envisagées.

1° But, durée, Espèces qui y seront protégées (pour les réserves de faune). Limite ;

2° Inventaire des droits d'usage s'exerçant à l'intérieur des limites protégées, accompagné des propositions tendant :

- Soit la reconnaissance pure et simple de leur plein exercice ;
- Soit à leur abandon, limitation, cantonnement ou rachat ;

3° Inventaire des droits, autre que les droits d'usage dont pourraient être grevés les terrains à réserver, accompagné des propositions tendant :

- Soit la reconnaissance pure et simple de leur plein exercice ;
- Soit à leur rachat amiable ;

4° Conditions dans lesquelles pourront s'effectuer, à l'intérieur des limites protégées, l'installation de nouveaux villages ou l'octroi de toutes concessions ou autres droits d'occupation ;

5° Conditions de circulation et de stationnement.

Le projet est soumis à l'agrément du ministre délégué à l'Agriculture qui, après approbation, la porte à la connaissance du public par tous les moyens de publicité réglementaire et par affichage, un mois durant, aux chefs-lieux des préfectures et sous préfectures intéressées.

Passé ce délai d'affichage, prouvé par des certificats des préfets et sous-préfets intéressés, si aucune contestation ne s'est manifestée, le projet est soumis au Conseil des ministres et la réserve créée par décret.

Si des contestations ont été soulevées par des personnes ayant pu faire opposition, le ministre délégué à l'Agriculture désigne une commission présidée par le préfet et comprenant deux députés originaires de la circonscription et le représentant du ministre délégué à l'Agriculture.

Cette commission est chargée d'étudier dans quelles conditions peuvent être abrogés, limités, cantonnés ou rachetés les droits d'usage et éventuellement de régler à l'amiable les contestations soulevées.

Cette commission doit entendre obligatoirement tous les chefs de village et collectivités locales intéressées. Elle se transporte sur place à cet effet. Elle établit sur un procès-verbal de ses débats qui est joint au projet soumis au Conseil des ministres.

Les personnes qui auraient des droits, autres que les droits d'usage, à faire valoir, pourront former opposition pendant les délais d'affichage du projet ; ainsi que pendant les trente jours qui suivent l'arrivée aux chefs-lieux des préfectures intéressées du *Journal officiel* contenant le décret de classement. Les oppositions seront enregistrées pour prendre date aux chefs-lieux des préfectures.

Les contestations pourront être réglées à l'amiable par la soumission prévue ci-dessus, sans quoi les opposants devront porter leurs revendications devant les tribunaux compétents.

DECLASSEMENT DES RESERVES NATURELLES INTEGRALES ET PARTIELLES ET DES PARCS NATIONAUX.

Article 6 :

Les projets de déclassement, total ou partiel ou de modification des statuts des réserves naturelles intégrales et partielles et des parcs nationaux sont étudiés et mis en forme comme les projets de classement, puis transmis au Conseil des ministres.

Article 7 :

Les infractions aux dispositions du présent décret sont recherchées, constatées, réprimées et réparées conformément aux règles générales en vigueur et prévues par la loi sur la protection de la faune et l'exercice de la chasse et le code forestier.

Article 8 :

Le présent décret sera publié au *Journal officiel* de la République.

Fait à Abidjan, le 15 septembre 1966.

FELIX HOUPHOUËT-BOIGNY.

Arrêté n° 1712 AGRI/EFC du 29 décembre 1966 fixant les conditions d'élimination ou d'éloignement des animaux nuisibles

LE MINISTRE DELEGUE A L'AGRICULTURE,

Vu la loi n° 65-255 du 4 août 1965, relative à la protection de la faune et à l'exercice de la chasse, en particulier en ses articles 20 et 21,

ARRÊTE :

CHASSE DE DESTRUCTION

Article premier :

Au cas où certains animaux protégés ou non constitueraient un danger pour des vies humaines ou causeraient des dégâts aux cultures ou au bétail, des autorisations de chasse individuelle ou de battue seront accordées par le ministre délégué à l'Agriculture après enquête sur place du chef de l'inspection forestière du ressort.

En cas d'urgence absolue, les préfets pourront accorder ces autorisations. Ils devront alors en rendre compte immédiatement au ministre délégué à l'Agriculture.

Par dérogation aux dispositions précédentes, les chasses ou battues aux animaux intégralement protégés ne pourront être autorisées que par le ministre délégué à l'Agriculture.

Les chasses de destruction doivent être sérieusement motivées. Elles sont temporaires exceptionnelles. Les faits de chasse qu'elles rendent possible sont soumis au contrôle étroit des agents de l'Administration.

Sauf exception à motiver, ces destructions effectuées par le moyen de chasse individuelle au fusil ; les battues n'étant qu'un pis aller à utiliser seulement lorsque les chasses individuelles sont impossible ou ont échoué.

Autant que possible, les chasses seront confiées aux agents des Eaux, Forêts et Chasse ou, à défaut, les sous-préfets ayant demandé l'autorisation de destruction, doivent rendre compte des chasses et des battues dans les moindres délais.

Ils indiqueront les motifs détaillés de chasse et la battue, les noms et qualités des chasseurs autorisés aux abattages, les jours et les lieux aux précis que possible des chasses ou des battues, les armes employées, les accidents s'il y a lieu, le nombre, le sexe et l'âge (adulte, jeune ou nourrisson) des animaux abattues et s'il s'agit d'éléphants, le poids et les dimensions des pointes. Le rapport sera adressé directement au ministre délégué à l'Agriculture.

Le chasseur volontaire pourra inscrire les animaux tués en chasse de destruction sur son carnet de chasse moyennant le paiement des taxes d'abattage.

Dans le cas contraire, les trophées et dépouilles doivent être remis à l'Administration.

La viande des animaux abattus sera laissée aux habitants et travailleurs des localités ayant subi des dommages.

Article 2 :

Pour le cas particulier des troupeaux nomadisant qui subiraient des dommages réels des faits des espèces prédatrices (hyène en particulier), il pourra être accordé annuellement par le ministre délégué à l'Agriculture, une autorisation de destruction de ces carnivores par les moyens appropriés.

La demande d'autorisation devra être présentée par le ministère de la Production animale qui indiquera les lieux où ces destructions doivent être opérées. Le service local des Eaux, Forêts et Chasse sera toujours avisé par avance, fera une enquête préalable et donnera un avis. Cette demande sera instruite par la direction des Eaux, Forêt et Chasse qui soumettra au ministre délégué à l'Agriculture avec avis motivé.

LEGITIME DEFENSE

Article 3 :

Aucune infraction ne peut être relevée contre quiconque a fait acte de chasse indûment, dans la nécessité immédiate de sa défense, de celle d'autrui ou de celle de son propre cheptel domestique ou de sa propre récolte. Toutefois, la provocation préalable des animaux demeure formellement interdite.

La preuve du cas de la légitime défense doit être fournie dans les délais les plus brefs aux agents locaux de l'Administration des Eaux, Forêt et Chasse.

Les dépouilles et trophées recueillis dans ce cas doivent être remis à l'Administration.

En cas d'abattage de bonne foi, par le titulaire d'un permis de grande chasse ou d'un permis de passager, d'un éléphant ayant des défenses de moins de cinq Kilo, son auteur ne sera pas considéré comme ayant commis un délit, s'il fait une déclaration administrative et se comporte en tous autres points comme dans un cas de légitime défense.

L'animal abattu figurera pour deux unités au décompte des animaux tués et les taxes d'abattage seront perçues en conséquence.

METHODE DE DETRUCTION AUTORISEES

Article 4 :

Pour assurer la protection de leurs récoltes et de leur cheptel domestique, les cultivateurs sont autorisés à utiliser des fosses et des pièges dans un rayon d'un kilomètre autour de leurs villages ou sur leurs terrains en cours de culture.

Ces pratiques sont toutefois interdites à l'intérieur des réserves de faune et parcs nationaux.

Les peaux de panthères éventuellement capturées par ces méthodes, pourront être librement commercialisées à charge pour l'acheteur d'en faire la déclaration immédiatement au poste administratif le plus proche.

Il sera perçu à cette occasion, une taxe dont le montant sera fixé annuellement et délivré un certificat d'origine identifiant la peau et qui sera exigée à l'occasion de toutes les transactions successives dont elle pourrait faire l'objet.

Article 5 :

Les infractions aux dispositions du présent arrêté sont recherchées, constatées, réprimées et réparées conformément aux règles générales en vigueur et prévues par la loi sur la production de la faune et l'exercice de la chasse.

Article 6 :

Les préfets, sous-préfets et le directeur des Eaux, Forêts et Chasse sont chargés, chacun en ce qui le concerne, de l'application du présent arrêté qui sera publié au *Journal officiel* de la République.

A. SAWADOGO.

Arrêté n° 621 AGRI/EFC du 29 mai 1967 réglementant la destination des produits de la chasse

LE MINISTRE DELEGUE A L'AGRICULTURE,

Vu la loi n° 65-255 du 4 août 1965, relative à la protection de la faune et l'exercice de la chasse, en particulier en son article 16,

ARRÊTE :

Article premier :

Les titulaires de permis de chasse peuvent librement disposer du gibier ou de la viande des animaux régulièrement abattus par eux dans la limite de leur consommation personnelle et familiale de celle de leurs employés les accompagnants à l'occasion de la chasse.

Le transport de la viande est donc limité à la quantité correspondant à ces consommations.

Le surplus de la viande doit être laissé à la disposition des usagers de la terre où a lieu l'abattage. La part qui revient aux ayants droit est déterminée par la tradition locale. Ceux-ci sont toujours valablement représentés par le chef du village ou un notable.

Article 2 :

L'achat, la vente, la cession ou l'échange de toute viande de chasse ou de tout gibier, quelle que soit l'origine ne sont autorisés qu'à titre strictement individuel et sans intermédiaire, entre celui qui a légalement abattu un animal à la chasse et toute personne qui désire se procurer de la viande de cet animal pour sa consommation personnelle ou celle de sa famille.

Le transport de cette viande ou de ce gibier est autorisé.

Ces opérations sont de toute façon et pour l'ensemble du territoire, interdites sur les marchés, dans le commerce ainsi qu'en faveur ou au profit de l'Administration civile ou militaire, ou des entreprises agricoles ou industrielles.

Article 3 :

Les infractions aux dispositions du présent arrêté sont recherchées, constatées et réprimées conformément aux règles générales en vigueur et prévues par la loi sur la protection de la faune et l'exercice de la chasse.

Article 4 :

Les préfets, sous-préfets et le directeur des Eaux, Forêts et Chasse sont chargés, chacun en ce qui le concerne, de l'application du présent arrêté qui sera publié au *Journal officiel* de la République.

A. SAWADOGO.

Arrêté n° 1068 du 29 septembre 1967
réglementant la chasse des crocodiles et varans dans un but commercial

LE MINISTRE DELEGUE A L'AGRICULTURE,

Vu la loi n° 65-255 du 4 août 1965, relative à la protection de la faune et l'exercice de la chasse, en particulier en son article 4,

ARRÊTE :

PERMIS SPECIAL DE CHASSE AUX CROCODILES ET VARANS

Article premier :

Tout individu désirant se livrer à la chasse des crocodiles et varans dans le but de commercialiser leurs peaux, doit être muni d'un permis spécial.

Ce permis est délivré par le directeur des Eaux, Forêts et Chasse, après avis favorable obligatoire du chef de la Région forestière intéressée.

Il donne le droit de tuer le nombre de crocodiles ou de varans précisé sur le permis, moyennant le paiement préalable d'une taxe d'abattage fixée à 200 francs par crocodile ou varan.

Article 2 :

Sont interdites, la destruction, la chasse et la capture des crocodiles et varans dont la peau est d'une largeur inférieure à 25 centimètres. Cette largeur se mesure :

- Pour les crocodiles, sur la face ventrale et se rapporte à la distance comprise entre les écailles cornées des deux flancs.
- Pour les varans, sur la largeur totale de la peau.

Article 3 :

Les crocodiles et varans, détruits, capturés ou tués en dehors des normes fixées ci-dessus, devront être remis dans plus brefs délais, à l'Administration la plus proche.

Article 4 :

Nonobstant les dispositions des articles ci-dessus, durant une période transitoire d'une année, les commerçants en peaux de crocodiles et varans seront autorisés à acheter et exporter des lots dont le pourcentage en peaux de tailles inférieures au minimum fixé n'excèdera pas 25 %.

Article 5 :

Les préfets, sous-préfets et le directeur des Eaux, Forêts et Chasse sont chargés, chacun en ce qui le concerne, de l'application du présent arrêté qui sera publié au *Journal officiel* de la République.

A. SAWADOGO.

Arrêté n° 1069 du 29 décembre 1967
réglementant la détention des animaux vivants par des particuliers

LE MINISTRE DELEGUE A L'AGRICULTURE,

Vu la loi n° 65-255 du 4 août 1965, relative à la protection de la faune et l'exercice de la chasse, en particulier son article 17,

ARRÊTE :

INTEGRALEMENT PROTEGES

Article premier :

La détention, le commerce et l'exportation des animaux intégralement protégés sont strictement interdits sauf par les titulaires de permis scientifiques ou de capture qui en auront reçu l'autorisation.

ANIMAUX PARTIELLEMENT PROTEGES ET OISEAUX SPECTACULAIRES

Article 2 :

Les titulaires de permis spéciaux de chasse sportive sont autorisés à détenir sous leur propre responsabilité, jusqu'à expiration de leur permis et sans autre formalité, dans la limite maximum de deux bêtes en même temps les animaux partiellement protégés dont l'abattage est autorisé par leur permis.

Il est entendu que tout animal détenu doit compter pour un animal tué figuré au carnet de chasse, toutefois, il ne donne pas lieu au paiement de la taxe d'abattage.

Les personnes non titulaires de permis spéciaux de chasse sportive, doivent obligatoirement déclarer à l'Autorité administrative, les animaux partiellement protégés qu'elles peuvent être amenées à recueillir. Elles peuvent être autorisées, sur leur responsabilité. La validité des autorisations délivrées par le Directeur des Eaux, Forêts et Chasse est interrompue par le départ de leur bénéficiaire.

A l'expiration des permis ou des autorisations de détention, les détenteurs d'animaux partiellement protégés doivent les remettre aux parcs zoologiques de la République ou aux détenteurs de permis scientifiques ou de capture autorisés à détenir des animaux d'espèces correspondantes. Ces derniers peuvent indemniser les détenteurs sans que cette indemnisation constitue un droit.

Le commerce et l'exportation des animaux partiellement protégés sont interdits, sauf pour les détenteurs de permis scientifiques ou de permis de capture.

Toutefois, l'exportation à titre de don d'animaux partiellement protégés à destination d'un parc zoologique ou d'un organisme scientifique peut être exceptionnellement autorisée par le ministre délégué à l'Agriculture. Il appartient dans ce cas à l'exportateur d'apporter la preuve du don et de payer les droits de sortie.

ESPECES PREDATRICES ET PETIT GIBIER

Article 3 :

La détention par des particuliers d'animaux non protégés sous leur propre responsabilité, est autorisée sans formalité.

L'exportation et le commerce des animaux non protégés sont possibles dans les conditions suivantes :

- Exportation sans but commercial par des particuliers d'animaux non protégés, précédemment régulièrement détenus par eux, sur autorisation du directeur des Eaux, Forêts et Chasse, sous réserve du paiement des droits de sortie et du respect de la réglementation sanitaire en vigueur ;

- Cession contre rémunération des animaux non protégés détenus par des particuliers aux seuls titulaires des permis scientifiques ou de capture et aux parcs zoologiques de la République.

Article 4 :

Les taxes annuelles pour la détention d'animaux sauvages en captivité prévues à l'article 77 de la loi sur la protection de la faune et l'exercice de la chasse seront fixées par arrêté conjoint du ministre délégué aux Affaires économiques et financières et du ministre délégué à l'Agriculture.

Article 5 :

Les infractions aux dispositions du présent arrêté sont recherchées, constatées et réprimées conformément aux règles générales en vigueur et prévues par la loi sur la protection de la faune et l'exercice de la chasse.

Article 6 :

Le présent arrêté sera publié au *Journal officiel* de la République.

A. SAWADOGO.

Arrêté n° 68 du 23 janvier 1967 fixant les tarifs des taxes et redevances en matière de chasse et de capture des animaux sauvages

LE MINISTRE DELEGUE A L'AGRICULTURE,

Vu la loi n° 65-255 du 4 août 1965, relative à la protection de la faune et l'exercice de la chasse, en particulier ses articles 9, 10 et 17 ;

Vu le décret n° 66-423 du 15 septembre 1966 fixant le régime des permis de chasse et les modalités de leur attribution en Côte d'Ivoire, en particulier son article 10,

ARRÊTE :

Article premier :

Les redevances perçues en application de la réglementation de la chasse à l'occasion de la délivrance des permis et les taxes d'abattage et droits complémentaires de capture sont fixées comme suit :

A – PERMIS DE CHASSE

Permis de petite chasse pour

Arme de traite	1.000 FCFA
Permis national de petite chasse	1.500 FCFA
Permis de moyenne chasse	5.000 FCFA
Permis de passager	10.000 FCFA
Permis de grande chasse	15.000 FCFA

B – TAXE D'ABATTAGE

Premier éléphant	10.000 FCFA
Deuxième éléphant	20.000 FCFA
Hippopotame	10.000 FCFA
Bongo	10.000 FCFA
Situtunga	5.000 FCFA
Hylochère	5.000 FCFA
Léopard ou panthère	3.000 FCFA
Buffle	2.000 FCFA
Lion	2.000 FCFA
Céphalophe à dos jaune	2.000 FCFA
Hippotrague	2.000 FCFA
Colobe magistrat	2.000 FCFA
Bubale	1.000 FCFA
Cob deffassa	1.000 FCFA
Cob de Buffon	1.000 FCFA

C – PERMIS DE CAPTURE

Pour les animaux protégés	30.000 FCFA
Pour les oiseaux protégés	5.000 FCFA
Pour les animaux non protégés	5.000 FCFA
Permis d'oisellerie valable pour 10.000 couples d'oiseaux non protégés	5.000 FCFA

D – DROITS COMPLEMENTAIRES DE CAPTURE

Exigibles au moment de la délivrance du permis de capture pour la moitié des animaux inscrits au permis.

MAMMIFERES

Mammifères intégralement protégés :

Eléphant (jeune accompagnant sa mère et femelle suitée)	50.000 FCFA
Hippopotame nain	40.000 FCFA
Lamantin	40.000 FCFA
Chimpanzé	20.000 FCFA

Mammifères partiellement protégés

Bongo, Situtunga	20.000 FCFA
Tous les autres (excepté Bubale, Cob de Buffon et Pangolin)	10.000 FCFA
Bubale, Cob de Buffon	5.000 FCFA
Pangolin	2.000 FCFA
Mammifères non protégés	1.000 FCFA

OISEAUX

Oiseaux intégralement protégés	5.000 FCFA
Oiseaux partiellement protégés	1.000 FCFA
Oiseaux spectaculaires	200 FCFA

REPTILES

Reptiles vivants	500 FCFA

E – TAXE DE DETENTION PAR DES PARTICULIERS D'ANIMAUX VIVANTS EN CAPTIVITE

La taxe de détention par les particuliers d'animaux vivants en captivité est fixée au 1/5 du droit complémentaire de capture correspondant. Cette taxe est payable chaque année et est valable pour l'année en cours.

F – TAXE D'ABATTAGE DES PANTHERES PRISES AUX PIEGES ET DONT LA PEAU EST COMMERCIALISEE

(Arrêté n° 1712 AGRI. EFC. du 29 décembre 1966, article 4) 3.000 francs.

Article 2 :

Le présent arrêté sera enregistré et publié au *Journal officiel* de la République.

A. SAWADOGO.

Arrêté nº 15 SEPN / SEB du 26 décembre 1972
portant modification de l'Arrêté n° 68 du 23 janvier 1967 fixant les tarifs des taxes et redevances en matière de chasse et de capture des animaux sauvages

LE SECRETAIRE D'ETAT CHARGE DES PARCS NATIONAUX,

LE SECRETAIRE D'ETAT AU BUDGET,

Vu la loi nº 65-255 du 4 août 1965, relative à la protection de la faune et l'exercice de la chasse, en particulier ses articles 9,10 et 17 ;

Vu le décret n° 66-423 du 15 septembre 1966, fixant le régime des permis de chasse et les modalités de leur attribution en Côte d'Ivoire, en particulier son article 10,

ARRÊTENT :

Article premier

Les redevances perçues en application de la réglementation de la chasse à l'occasion de la délivrance des permis et les taxes d'abattage sont fixées comme suit :

A – PERMIS DE CHASSE

Permis de petite chasse pour arme de traite	1.000 FCFA
Permis de petite chasse nationale	2.000 FCFA
Permis de moyenne chasse	20.000 FCFA
Permis de passager	15.000 FCFA
Permis de grande chasse	30.000 FCFA

B – TAXE D'ABATTAGE

Premier éléphant	15.000 FCFA
Deuxième éléphant	30.000 FCFA
Hippopotame	15.000 FCFA
Situtunga	5.000 FCFA
Bongo	20.000 FCFA
Hylochère	5.000 FCFA
Léopard ou panthère	20.000 FCFA
Buffle	10.000 FCFA
Lion	20.000 FCFA
Céphalophe à dos jaune	5.000 FCFA
Hippotrague	10.000 FCFA
Colobe magistrat	5.000 FCFA
Bubale	10.000 FCFA
Cobe deffassa	10.000 FCFA
Cobe de Buffon	5.000 FCFA

Pour les animaux protégés	50.000 FCFA
Pour les oiseaux protégés	10.000 FCFA
Pour les animaux non protégés	10.000 FCFA
Permis d'oisellerie valable pour 1000 couples d'oiseaux non protégés	10.000 FCFA

Article 2 :

Le présent arrêté annule toutes dispositions antérieures et notamment l'arrêté n° 68 du 23 janvier 1967 des paragraphes A, B, et C de son article premier.

Article 3 :

Le présent arrêté sera enregistré et publié au *Journal Officiel* de la République de Côte d'Ivoire.

Les secrétaires d'Etat chargés du Budget et des Parcs Nationaux

Le Ministre de l'Economie et des Finances

Arrêté n° 003/SEPN/CAB du 20 Février 1974
portant fermeture de la chasse

LE SECRETAIRE D'ETAT CHARGE DES PARCS NATIONAUX

Vu le décret 71-478 fixant les attributions du Secrétaire chargé des Parcs Nationaux et portant organisation du Secrétariat d'Etat ;

Vu l'arrêté n° 6 SEPN/CAB du 21 Décembre 1973 portant ouverture de la chasse pour la saison 1974 ;

Vu l'arrêté 1615 du 27 Décembre 1965 fixant la période d'ouverture de la chasse ;

Vu la loi nº 65-255 du 4 Août 1965, relative à la protection de la Nature.

ARRÊTE :

ARTICLE : 1 - La chasse est fermée sur toute l'étendue de la République de Côte d'Ivoire pour compter du 1er Janvier 1974.

ARTICLE : 2 - L'ouverture de la chasse fera l'objet d'un arrêté ultérieur pris par le Secrétaire d'Etat chargé des Parcs Nationaux.

ARTICLE : 3 - Les Préfets, Sous-Préfets, les Commandants de la Gendarmerie, les Officiers de la Police Judiciaire, le Secrétaire d'Etat chargé de la Reforestation, le Directeur des Douanes, le Directeur de la Protection de la Nature, le Directeur des Parcs Nationaux, les Chefs des Régions Forestières et les Chefs d'Inspection chasse sont chargés chacun en ce qui le concerne, de l'exécution du présent arrêté qui sera enregistré, publié et communiqué partout où besoin sera./-

DIFFUSION :

- Prési/République ……………………….3
- Assemblée Nationale ………………...3
- Conseil Economique ……………………3
- Cours Suprême ……………………….30
- Tous les Ministères ………………….30
- Ministère de l'Intérieur ………………..119
- Ministère de l'Agriculture …………….5
- Réforestation …………………………119
- A.I.P. …………………………………2
- Commandant Gendarmerie …………119
- Fraternité-Matin ……………………….3
- Hebdomadaire …………………………3
- Parcs Nationaux ………………………30
- Direction Protection Nature …………30
- Direction Douanes ……………………30
- Direction Sureté Nationale ………….30
- J.O.C.I. ………………………………3

LE SECRETAIRE D'ETAT

K. ATTOBRA.
