# Supplementary material for: Ivory Coast without ivory: Massive extinction of African forest elephants in Côte d’Ivoire
Source: PLoS One. 2020 Oct 14;15(10):e0232993. doi: 10.1371/journal.pone.0232993 (PMC7556483; doi:10.1371/journal.pone.0232993)
Supplement: S6 File — (PDF) [file pone.0232993.s009.pdf]

MINISTERE DES EAUX ET FORETS **REPUBLIQUE DE COTE D'IVOIRE**

------------------- **Union – Discipline - Travail**

DIRECTION GENERALE

DES EAUX ET FORETS

DIRECTION DE LA FAUNE ET

DES RESSOURCES CYNEGETIQUES

SOUS-DIRECTION DE LA FAUNE

ET DE LA CHASSE

TEXTS GOVERNING WILDLIFE PROTECTION AND THE HUNTING EXERCISE

May 2013

SUMMARY

Law No. 94-442 of August 16, 1994 amending Law No. 65-255 of August 4, 1965 1
relating to the protection of wildlife and the practice of hunting 1
Law n ° 65-255 of August 4, 1965 relating to the protection of fauna 8
and the practice of hunting 8
Decree n ° 97-130 of March 07, 1997 24
regulating the possession of ivories 24
Decree n ° 66-423 of September 15, 1966 28
fixing the hunting license regime and the terms of their attributions in 28
Republic of Côte d'Ivoire 28
Decree n ° 66-424 of September 15, 1966 38
relating to the hunting guide license 38
Decree n ° 66-425 of September 15, 1966 44
regulating the traffic, movement, import, exploitation of animal trophies 44
protected and spectacular and their remains 44
Decree n ° 66-433 of September 15, 1966 47
on the status and regulations of the classification procedure for nature reserves, ... 47
full or partial and national parks 47
Order n ° 1712 AGRI / EFC of December 29, 1966 52
setting the conditions for the elimination or removal of pests 52
Order n ° 621 AGRI / EFC of May 29, 1967 55
regulating the destination of hunting products 55
Order n ° 1068 of September 29, 1967 57
regulating the hunting of crocodiles and monitor lizards for commercial purposes 57
Order n ° 1069 of December 29, 1967 59
regulating the keeping of live animals by individuals 59
Order No. 68 of 23 January 1967 61
fixing the tariffs of taxes and royalties on hunting 61
and capture of wild animals 61
Order No. 15 SEPN / SEB of 26 December 1972 64
amending Decree No. 68 of 23 January 1967 fixing the rates of taxes and 64
fees for hunting and capturing wildlife 64
Order n ° 003 / SEPN / CAB of February 20, 1974 66

wearing hunting closure 66

Law No. 94-442 of August 16, 1994 amending Law No. 65-255 of August 4, 1965 relating to the protection of wildlife and the practice of hunting

THE NATIONAL ASSEMBLY HAS ADOPTED

THE PRESIDENT OF THE REPUBLIC PROMULGATES THE LAW WHOSE CONTENT FOLLOWS:

First article :

Article 4 of Law No. 65-255 of August 4, 1965 on the protection of wildlife and the practice of hunting is thus complete:

New Article 4:

Wildlife protection is ensured by the following processes:

1. Constitution and maintenance of integral or partial natural reserves and national parks, such as defined in Article 2 of the International Conservation of London of 8 November 1933 relating to the conservation of African flora and fauna in their natural state;
2. Constitution and maintenance of total or partial reserves of fauna established either for all fauna, either for certain species only or under certain conditions;
3. Determination and development of wildlife areas;
4. Full or partial protection of rare or endangered animal species, or presenting scientific interest, or necessary for biological balance, or particularly useful for humans, or whose operation for cynegetic or visual tourism has an economic interest or educative ;
5. Technical measures to limit the practice of hunting, in particular protection of females and young, eggs and broods, ban on night hunting, fixing of closing periods, limitation of hunting tables, limitation of the number of weapons or the use of certain weapons;
6. Prohibition of certain means of hunting and in particular land or air motor vehicles, encircling fires, dazzling lights, poisons, drugs, explosives, nets, pits and traps;
7. Supervision carried out by specialized staff with the help of the various departments with responsibilities repression control: Forest Police, Gendarmerie, Customs, National and municipal police;
8. Repression the effectiveness of which will be sought by the application of legal presumptions of guilt, by the exemplarity of penalties and the speed of intervention;

9. Global education of the population, both by teaching at different levels and by means audiovisual media with a view to raising national awareness of the concept of protection of nature ;
10. Raising of wild animals in specially equipped fixed places.

Article 2:

Article 9 of Law No. 65-225 of August 4, 1965 is repealed and replaced by the new Article 9:

New Article 9:

Five categories of permits are created:

1. Small hunting licenses which have two degrees;
2. The local permit at the level of the sub-prefecture, for the exclusive benefit of farmers or breeders holders of a permit to carry milking weapons and giving the right to hunt only the animals mentioned in Annexes III of this law and this outside the wildlife management zones provided for in Article ;
3. The national license valid for unprotected animals giving the right to hunt with a weapon improved, throughout the territory, the animals of Annexes II and III, within the limits provided to these annexes;
4. Sport hunting licenses authorizing the slaughter of a determined number of so-called animals partially protected and comprising three degrees;
5. The medium hunting license;
6. Short-term passenger tourist hunting permit;
7. Big game hunting license;
8. Commercial capture permits authorizing the capture, possession, transfer, export of live wild animals excluding fully protected species;
9. Scientific hunting or capture permits granted exceptionally to representatives official scientific bodies for slaughter or capture for specific scientific purposes animals of fully protected species;
10. Hunting permits for farmed wild animals;

Article 3:

The title of Chapter V and the provisions of Articles 15 and 16 are amended as follows:

CHAPTER V (NEW)

Hunting products

New Article 15:

The competent administrative authority regulates, if necessary, the marketing, import and the export of the remains of wild animals, including trophies. The expression "trophies" means any dead animal mentioned in Annexes I and II or any part not perishable or naturalized of this animal, whether or not incorporated into a worked object. The term "meat" refers to fresh or preserved meat, fat and blood from wild animals.

New Article 16:

The competent authority sets the conditions under which it authorizes marketing in all its forms of hunting meat.

Article 4:

Article 39 of Law No. 65-255 of August 4, 1965 is supplemented as follows:

The conditions of application of this law are determined by decree and in particular as regards:

- The constitution of reserves, national parks and wildlife development zones;
- Representation of the Administration before the courts;
- The applicable transaction procedure;
- The conditions for issuing hunting and capture permits, hunting licenses and the methods of granting the right to hunt;
- The conditions for setting up and operating wild animal breeding farms.

Article 5:

The annexes to Law No. 65-255 of August 4, 1965 are repealed and replaced by the new annexes below.

Article 6:

This law will be executed as state law and published in the Official Gazette of the Republic of Côte d'Ivoire.

Done in Abidjan, August 16, 1994.

Henri Konan BEDIE.

APPENDICES

To law n ° 94-442 of August 16, 1994 amending law n ° 65-255 of August 04, 1965 relating to wildlife protection and hunting.

APPENDIX I
FULLY PROTECTED SPECIES

Fully protected wild animals whose capture and hunting (including those of their young or of their eggs) are prohibited except to holders of scientific permits within the limits and with the means registered with the license:

Mammals

- Elephant (*Loxodonta africana africana*, *Loxodonta africana cyclotis*);
- Dwarf hippopotamus (*Choeropsis liberiensis*);
- Lamatin (*Trichechus senegalensis*);
- Chimpanzee (*Pan troglotydes*);
- Aquatic chevrotain (*Hyemesenus aquaticas*);
- Magistrate Colobus (*Colobus polykomos*);
- Zebra duiker (*Cephalophus zebra*);
- Giant land pangolin (*Manis gigantea*);
- Microprotamogale (*Microprotamogale lamottei*);
- Royal antelope (*Neotragus pygmeus*);
- Hylochère (*Hylochoerus meinerzhageni*);
- Potto de Bosman (*Perodictucus potto*);
- Galago de Demidof (*Galago demidovii*);
- Bongo (*Tragelaphus euryceros*);
- Leopard (*Phanthera pardus*);
- Lion (*Panthera leo*);
- Cercopithecus Diane (*Cercopithecus diana*);
- Colobus Bai (*Colobus badius badius*);
- Orycterope (*Orycteropus afer*);
- Jentink's duiker (*Cephalophus jentinki*);
- Yellow-backed duiker (*Cephalophus sylvicultor*).

Reptiles

- Nile crocodiles (Crocodylus niloticus);
- Long-nosed crocodiles (Crocodylus cataphractus);
- Forest or marsh crocodiles (Osteolaemus tetraspis);
- Sea turtles (Chelonidae).

Birds

- Small serpentine (Polyboroides radiatus);
- White-breasted guinea fowl (Agelastes meleagrides);
- All vultures;
- Great Abyssinian Hornbill (Bucorvus abyssinicus);
- Marabou (Leptoptilos crumeniferus);
- Great egret (Egretta aiba);
- Little Egret white form (Egretta garzetta);
- Little Egret gray form (Egretta gularis);
- Crowned crane (Balearica pavonina);
- Jabiru (Ephippiorhynchus senegalensis);
- All Herons, Storks and Ibis;
- All the Eagles.

APPENDIX II

List of partially protected animals whose hunting and capture are authorized by holders of sport hunting license or capture license within the limits indicated on the licenses:

Mammals

- Pangolin with tricuspid scales (Manis tricuspis);
- Long-tailed pangolin (manis tetradactyla);
- Dwarf anomalures (Idiurus macrotis);
- Buffalo (Syncerus caffer);
- Hippotrague (Hippotragus equinus);
- Cobe defassa (Kobus ellispsiprymnus defassa);
- Bubale (Alcephalus buselaphus);
- Lycaon (Lycaon pictus);
- Spotted hyena (Crocuta crocuta);
- Jackal with striped flanks (Canis adustus);
- Serval (Leptairus serval liposticta);

- White-cheeked otter (Aonyx capensis);
- Ratel (Meilivora capensis);
- Common Zoril (Ictonyx striatus);
- Nandinie (Nandinia binotata);
- Mone (Cercopithecus mona);
- Green monkey (Cercopithecus aethiops);
- Black duiker (Cephalophus niger);
- River hog (Potamochoerus porcus).

Reptiles

- Seba Python (Python sebae);
- Royal python (Python regius).

Birds

- Suitane hen (Porphyrio porphyrio);
- Jacana (Actophilomis africana);
- All diurnal raptors except Serpentines, Vultures, eagles, Accipitriformes;
- Nocturnal raptors: all Owls;
- Parrots: all Psittaciformes;
- Touracos, Musophages, Cuckoos;
- Green-cheeked Eurasian (Apaloderma narina);
- All Peaks and Beards;
- All Kingfishers, Rolliers, Calaos (except the great hornbill and Bee-eaters) ;
- Metal work, Loriot and Souimangas.

APPENDIX III

Wild animals including hunting authorized for customary users, for license holders small hunting and special sports permits within the limits of general hunting latitudes authorized by law:

Mammals

- Harnache bushbuck (Tragelaphus scriptus);
- Buffon cob (Kobus kob) ;
- Redunca (Redunca redunca);
- Grimm's duiker or doe pig (Cephalophus grimmia);

- Black-banded duiker (Cephalophus niger);
- Red-sided duiker (Cephalophus rufilatus);
- Maxwell's duiker (Cephalophus maxwelli);
- Ourebi (Ourebia ourebi);
- Warthog (Phacochoerus aethiopicus) ;
- Tree hyrax (Dendrohyrax arboreus);
- Lievre (Lepus whytei);
- Grassclover (Tryonomis swinderianus);
- Porcupine (Hystrix cristata);
- Atherurus (Atherurus africanus):
- All Squirrels;
- White-bellied hedgehog (Erinaceus aibiventris);
- Golden cat (Profelis aurata);
- Wild cat (Felis silvestris);
- Civet (Viverra civetta);
- Genettes (Genette pardina, Genetta tigrina);
- Poiane (Poiana richardsoni);
- Mongoose ichneumon (Herpestes ichneumon);
- Red mongoose (Herpestes sanguineus);
- Brown mongoose (Mungos obscurus);
- Mango (Mungos gambianus);
- Cynocephalus (Papio cynocephalus);
- Patas (Erythrocebus patas);
- Nodding (Cercopithecus petaurista);
- Potamogale (Potamogale velox).

Reptiles

- All Turtles (except Chelonidae);
- Savannah monitor lizard (Varanus exanthematicus);

Birds

- All: Ducks, Birds, Teals;
- All: Francolins, Pintades, Quail, Poule de roche;
- All: Plovers, Lapwing, Knights, Curlew, Oedicnemes, Becassines.

Law n ° 65-255 of August 4, 1965 relating to the protection of fauna
and the practice of hunting

THE NATIONAL ASSEMBLY HAS ADOPTED,
THE PRESIDENT OF THE REPUBLIC PROMULGATES THE LAW WHOSE CONTENT FOLLOWS:

TITLE ONE
GENERAL

First article :

- Under the terms of this law and the texts which will be taken for its application, the fauna is constituted by all wild animals living in freedom in their natural environment, classified among mammals (to except bats, rats and mice), and among birds, crocodiles, monitor lizards and pythons.

The animals that make up the fauna are divided as follows:

- Species said to be protected, classified and listed in Appendix I, rare or threatened with extinction or very localized or of scientific interest whose disappearance would constitute an irremediable loss, or presenting a particular utility for man and his activities including the interest of sport hunting and the value of trophies;
- The so-called spectacular species listed in Appendix II, birds in particular which, by their shape, their habitat, their way of life, constitute the adornment of nature and thus intervene in the tourist interest the regions where they live;
- The so-called predatory species, listed in Appendix III, which participate in the biological balance in wildlife areas;
- The so-called small game species, listed in Appendix IV, which are neither protected nor mentioned in the previous categories, which are sought after for traditional hunting and small-scale hunting and which participate traditionally locally sourced;
- So-called harmful species, which constitute a permanent danger or cause damage in residential or agricultural or pastoral areas, which will be designated by the administrative authority notwithstanding their belonging to Annexes III and IV.

Article 2:

Animals held in captivity or the remains of wild animals do not become the property of individuals who if these animals were captured or killed in accordance with the regulations made for the exercise of capture or hunting, or for duly authorized disposal or destruction.

The remains include all or part of the dead animal, including fresh or preserved meat.

TITLE II: PROTECTION OF WILDLIFE

Article 3:

The protection of fauna tends to ensure the conservation and the qualitative and quantitative enrichment of animals wild species living naturally in the country, both on areas falling within the domain of the State than on private land.

Article 4:

Wildlife protection ensured by the following process:

1° Establishment and maintenance of integral or partial nature reserves and national parks, such as defined in article 2 of the London International Convention of 8 November 1933, relating to the conservation African fauna and flora in their natural state;

2° Constitution and maintenance of total or partial wildlife reserves established either for all wildlife, or for certain species only or under certain conditions;

3° Determination and development of wildlife areas;

4° Full or partial protection of rare or endangered animal species, or those presenting a scientific interest, or necessary for biological balance, or particularly useful for humans, or of which the exploitation for hunting or visual tourism has an economic or educational interest;

5° Technical measures to limit the practice of hunting, in particular protection of females and young, eggs and broods, ban and hunting at night, fixing of closing period, limitation of hunting boards, limitation of the number of weapons or the use of certain weapons;

6° Prohibition of certain means of hunting and in particular of land or air motor vehicles, fires encirclers, dazzling lights, poisons, explosive narcotics, pit nets and traps;

7° Supervision carried out by specialized staff with the help of the various departments with responsibilities of control and repression: water, forests and hunting, gendarmerie, customs, national and municipal police;

8° Repression the effectiveness of which will be sought by the application of legal provisions of guilt, by the exemplary nature of the penalties and the speed of intervention;

9° Global education of the population both through education at different levels and by means audiovisual media with a view to raising national awareness of the concept of nature protection.

Article 5:

The classification of full or partial nature reserves and national parks is decided by decree. Strict nature reserves and national parks are exempt from all user rights and are part of the classified forest estate.

Article 6:

The competent administrative authority sets the conditions for issuing special authorizations written in which it is forbidden to enter, to travel, including by air at low altitude, to camp and carry out any scientific research in nature reserves and regulate the circulation and camp inside national parks.

TITLE III: HUNTING AND CAPTURE

FIRST CHAPTER

ACTS OF HUNTING AND CAPTURE

Article 7:

Under the terms of this law, "hunting" means any act tending either to injure or to kill, for appropriate or not all or part of his body, a wild animal within the meaning of Article 1 of the this law, or to destroy the eggs of the birds or reptiles mentioned in this same first article.

Any act tending to deprive of its liberty, a wild animal referred to in article first or to collect and remove from their natural place of hatching, the eggs of the birds or reptiles mentioned in article 1.

Article 8:

No one may, apart from the exceptions provided for in Articles 11 and 12 (traditional hunting) and Articles 20, 21 and 22 in self-defense, engaging in any act of hunting or capturing without a permit.

CHAPTER II

HUNTING AND CAPTURE LICENSE

Article 9:

Four categories of permits are created:

1° Small hunting licenses which have two degrees:

a) The local permit at the level of the sub-prefecture for the exclusive benefit of farmers or breeders holders of a permit to carry milking weapons and giving the right to hunt only the animals mentioned in Annexes III and IV wildlife development zones provided for in Article 18.

b) The national license valid for unprotected animals and giving the right to hunt with a weapon improved throughout the territory the animals, Annexes II, III and IV, within the limits provided for in these annexes.

2° Special sport hunting permits authorizing the slaughter of a determined number of so-called animals partially protected and comprising three degrees:

a) The medium hunting license;
b) Short-term passenger tourist hunting permit;
c) Big game hunting license.

3° Special commercial capture permits authorizing the capture, detention, transfer, export live wild animals, excluding fully protected species.

4° Specific hunting or capture permits granted exceptionally to representatives official scientific bodies for the slaughter or capture for specific scientific purposes of animals fully protected species.

Article 10:

The provisions relating to the nature, allocation, slaughter latitudes, control, publicity, duration, upon expiry of these various permits as well as the quality and responsibilities of the holders, are defined by decree.

CHAPTER III

TRADITIONAL HUNTING

Article 11:

The small hunt for unprotected animals practiced according to the tradition, outside the reserves and its zones of protection, with traditional weapons of local manufacture excluding any firearm and any process prohibited by this law and its implementing decrees, is qualified as "traditional hunting".

Article 12:

Anyone within the limits of the sub-prefecture of their location is considered a “traditional hunter”. of residence, hunting for his food and that of his family, under the conditions provided for in Article 11.

By way of derogation from Article 8, the traditional hunter is authorized to hunt without a license, however respecting when hunting is closed.

CHAPTER IV

HUNTING GUIDES

Article 13:

Anyone who organizes for a fee on behalf of others, hunting operations is deemed to be a "hunting guide", hunting or capture or wildlife photography expeditions.

Article 14:

No one can exercise the profession of hunting guide unless he holds a special license.

CHAPTER V

HUNTING PRODUCTS

TROPHIES AND DROPPINGS

HUNTING MEAT

Article 15:

The administrative authority regulates the traffic and movement of trophies of protected and spectacular animals and the remains of unprotected animals.

The expression "trophies" designates any dead or alive animal mentioned in Annexes I and II, its teeth, tusks, horns, bones, scales, claws, hooves, skin, hair, eggs, plumage or any non-perishable part of the animal whether or not included in a worked or transformed object, unless they have lost their original identity; the term "Meat" means fresh and preserved meat, fat and blood.

Article 16:

The exchange, transfer, purchase, barter and sale in any form of game meat on markets and in commerce, as well as for or for the benefit of the civil or military administration or agricultural or industrial enterprises are prohibited.

The administrative authority determines the tolerances in favor of traditional hunters and holders of local hunting license, within the village limits, and in particular for the benefit of the village hunter, when his profession is consecrated by tradition, and regulates the transport of hunting meat.

CHAPTER VI

DETENTION OF WILD ANIMALS IN CAPTIVITY

Article 17:

The competent administrative authority sets the tolerances and modalities of detention by individuals, in apart from any commercial purpose, a small number of animals in captivity

obtained under conditions regulatory or incidental. Keeping wild animals in captivity by persons other than license holders scientific, are subject to the payment of annual fees.

CHAPTER VII
WILDLIFE DEVELOPMENT ZONES

Article 18:

For the execution of article 4, paragraph 3, of this law, the administrative authority determines areas assigned to wildlife management and in which the practice of traditional hunting will be prohibited and small-scale hunting as well as the systematic destruction of predators.

In these areas, hunting and capture will only be allowed to holders of special permits or under the control of the Administration.

Article 19:

The right to hunt in wildlife development zones may be the subject of a concession in favor of hunting companies within the framework of the specifications of the planning regulations.

CHAPTER VIII
PROTECTION OF PERSONS AND PROPERTY
SELF-DEFENSE

Article 20:

The conditions for the elimination or removal of animals harmful to people and property are set by the administrative authority which determines the conditions under which hunting will be prohibited in harvests during or in certain permanent plantations as a safety measure for people or crop protection.

Article 21:

In the event that certain animals, protected or not, constitute a danger or cause damage, the administrative authority may, by temporary and exceptional measure, ensure or authorize its continuation or destruction after an on-site investigation.

Article 22:

No offense can be noted except prior provocation of animals against anyone who has committed hunting unduly, but in the immediate need of his own defense or that of others or the protection of his domestic livestock or his own harvest. In the event of the slaughter of a fully protected animal, the proof self-defense must be provided as soon as possible to agents of the Administration.

CHAPTER IX
WEAPONS AND AMMUNITION

Article 23:

Weapons and munitions of war composing or having composed the regulatory armament of forces military, militia or police cannot be used for hunting.

Article 24:

The use of automatic repeating weapons capable of firing in bursts is prohibited for hunting.

Article 25:

No one may, save for the exceptions provided for in Articles 26 and 27, obtain a hunting license if he does not hold a license to carry a weapon valid as a title.

Article 26:

Licensed hunting guides, holders of the license provided for in Article 14, may use weapons of hunting available to their clients and obtain special sport hunting permits for them notwithstanding the provisions of Article 25.

In the event of offenses committed by their clients, the hunting guides are responsible for the payment of fines that may be imposed, except for them to prove that they have done everything in their power to prevent the commission of the offense.

Article 27:

Minor children between the ages of eighteen and twenty-one and the spouse of a holder of a license to carry a weapon may obtain a hunting license upon written request from the holder proving the age and parentage of the beneficiary.

TITLE IV
REPRESSION
FINDINGS OF OFFENSES

Article 28:

Any individual found in breach of this law by an authorized agent but not having the status of a judicial police, will, if he cannot validly prove his identity and his residence, conducted immediately before the nearest judicial police officer or, where applicable, before the Public Prosecutor Republic or the judge of the court section.

Article 29:

The flagrante delicto procedure will be applicable in this matter.

ACTIONS AND PROURSES

Article 30:

Actions and prosecutions are exercised directly by the administrative authority before the courts competent without prejudice to the right which belongs to the public prosecutor to these courts.

PRESCRIPTIONS

Article 31:

Hunting offenses are prescribed by one year, from the day on which these offenses were noticed.

PRESUMPTIONS OF CRIMES

Article 32:

Is presumed guilty of an offense against the legislation on hunting and will be prosecuted under the same conditions that if the alleged criminal act had actually been noted that anyone:

1° Is found carrying a loaded weapon on the limits of an integral reserve or a national park or a special wildlife reserve;

2° Is found carrying a weapon, even unloaded, accompanied by ammunition within said areas reserved;

3° Outside a closed property or an urban agglomeration or the inhabited limits of a village is found at night carrying a weapon, even unloaded, and a dazzling lamp, installed or not, at the same time, adaptable to forehead, head, hairstyle or gun;

4° Outside the same places and outside a cultivated land carrying crops is found carrying a weapon of busy, either when hunting is closed, or at night;

5° At all times and in all places, is in possession of a living or dead protected animal or a part of this animal if it cannot be proven by the exhibition of a regulatory permit, or in any other way, that he was authorized either to slaughter or to keep the said animal.

PENALTIES

Article 33:

Violations of this law and its implementing decrees are punished:

1°A fine of 3,000 francs to 300,000 francs, and imprisonment for two months to one year or one of these two penalties only;

2° The confiscation of injured or captured animals or the remains of killed animals, or a order to pay an amount equal to their value if they cannot be conveniently seized.

These penalties may also be accompanied by:

1° The confiscation of arms, ammunition, devices, materials having served to commit the offense. The vehicle automobile or other, having been used deliberately for hunting purposes is considered as material liable to confiscation, especially when it has been used as a means of chasing game, such as device dazzling by its headlights or to transport delinquent hunters inside a reserve or of a national park;

2° The forfeiture of the permit and, possibly, the temporary or definitive deprivation of the granting of any other hunting or capture permit or license.

Article 34:
The fines or imprisonment provided for in the previous article are doubled when one of the following three conditions is met:

1° Offense committed in a reserve or national park;

2° Offense committed at night with illuminating device;

3° Recurrence.

Article 35:
The penalties are tripled when two of the three circumstances provided for in Article 34 are found gathered at the time of the offense.

Article 36:
Imprisonment will be compulsory, without the benefit of a suspension and without mitigating circumstances, when the perpetrator of an offense committed in a reserve or national park will have already been convicted for the first time for acts analogues within the time limits for recidivism provided for by this law.

JUDGMENTS AND TRANSACTIONS

Article 37:
Except in cases where the prison sentence is compulsory, offenses against hunting regulations and wildlife protection may be the subject of a transaction between the administrative

authority and the offender. The transaction can take place before or after judgment. However, the judgment become final, the transaction can only relate to the pecuniary convictions he pronounces.

Article 38:

There is a recurrence in matters of hunting and wildlife protection when, in the three years preceding the offense, the offender has already been the subject of a final conviction or has benefited from a settlement for an offense provided for by this law and its implementing regulations.

In the event of a transaction, the competent administrative authority will provide the court with a copy of the deed signed by the person concerned and by the competent administrative authority.

Article 39:

The conditions of application of this law are determined by decree and in particular as regards:

- The constitution of reserves, national parks and wildlife development zones;
- Representation of the Administration before the courts;
- The applicable transaction procedure;
- The conditions for issuing hunting and capture permits, hunting guide licenses and the terms of concession of the right to hunt.

Article 40:

This law will be executed as state law and published in the Official Journal of the Republic of Côte d'Ivoire.

Done in Abidjan, August 4, 1965.

FELIX HOUPHOUET BOIGNY.

APPENDIX I: PROTECTED SPECIES

CLASS A

List of fully protected wild animals, including capture, including those of their young or their eggs, are prohibited except to holders of scientific permits within the limits and with the means registered with the permit.

MAMMALS:

Dwarf hippopotamus: Choeropsis liberiensis.
Elephant (young accompanying its mother and followed female): *Loxodonta africana*.
Manatee: Trichechus senegalensis.
Chimpanzee: Pan satryrus verus.

BIRDS :

Serpentary Messenger: Sagittarius serpentarius.
White-breasted guinea fowl: Agelastes meleagrides.

CLASS B

List of so-called specific partially protected wild animals including hunting and capture, including including those of their young or their eggs, are only allowed to holders of capture permits in the limits set out in permits and holders of special big game and tourist hunting permits passenger, but only individually as a trophy or collector's item.

MAMMALS:

Aquatic chevrotain: Hyemoschus aquaticus.
Orycterope: Orycteropus afer.
Magistrate Colobus: Colobus polykomos.

Jentink's duiker: Cephalophus jentinki.
Yellow-backed duiker: Cephalophus sylvivultor.
Zebra duiker: Cephalophus zebra.
Giant land pangolin: Smutsia (syn. Manis) gigantea.
Common tree pangolin: Phataginus (syn. Manis) tricuspis.
Long-tailed tree pangolin: Uromanis (syn.Manis) longicaudata.
Potamogale: Potamogale velox.
Micropotamogale: Micropotamogale lamottei.
Pygmy Neotrague or Royal Antelope: Neotragus pygmaeus.
Hylochère: Perodicticus potto.
Galapos: Genus Galago.
Anomalures or Flying Squirrels: Genus Anomalurus, Anomalurops.

BIRDS :

All vultures: Aegypriidae family.
Great Abyssinian Hornbill: Bucorvus Abyssinicus.
Marabout: Leptoptilos crumenirefus.
Intermediate egret: Egretta (syn. Casmerodius) alba.
Gazette white form: Egretta garzetta garzetta.
Gazette gray form: Egretta garzetta gularis.
Crowned crane: Balearica pavonica.
Jabiru: Ephippiorhyncus senegalensis.
Fish eagle: Haliaetus (syn. Cuncuma) vocifer.

CLASS C

List of partially protected wild animals known as hunting, including hunting only individuals adults is authorized for holders of special sport hunting permits within the limits of the table set for each degree and whose capture including that of their young is authorized to holders of capture permits within the limits indicated in the permits.

MAMMALS:

Elephant: Loxodonta (syn. Elephas) africana.
Buffalo: Bubalus (syn. Syncerus) caffer.
Amphibious hippopotamus: Hippopotamus amphibius.
Bongo: Boocercus euryceros.
Situtonga: Linnotrgus spokoi.

Hippotrague: Hippotragus equinus.
Cob defassa (Waterbuck): Kobus deffa defassa.
Bubale: Alcolaphus major, Acolaphus lelwel.
Buffon's Cob: Adenota kob.
Leopard or African Panther: Panthera pardus.
Leo: Leo leo.

APPENDIX II: SPECTACULAR BIRDS

List of so-called spectacular birds whose hunting is prohibited to customary users and whose slaughter by holders of national small-game hunting permits and special sports permits is only authorized in restricted limits as trophies.

BIRDS :

Herons, Cigognes and Ibis: among the Ardeiformes.
Sultana hens and Jacanas: among the Ralliformes.
Diurnal raptors (other than Serpentaire, Vautorus, Fish eagles which are protected): all Accipitriformes.
Nocturnal raptors: all Owls.
Parrots: all Psittaciformes.
Touracos, Musophages and Cuckoos: among the Cuculiformes.
Couroucou or Trognon: among the Trogoniformes.
Woodpeckers and Barbus: among the Piciformes.
Kingfishers, Rollers, Calaos and Bee-eaters: among the Coraciadiformes.
Metallic blackbirds, Oriole and Souimangas (improperly called flybirds): among the Passeriformes.

APPENDIX III: PREDATORY SPECIES

List of so-called predatory species whose slaughter is normally authorized in residential areas and of agricultural, poultry or pastoral operations, under the conditions provided for customary hunting for permits for hunting of all categories as well as for the defense of crops or domestic livestock, but including hunting will be regulated in areas designated for wildlife and hunting development:

CARNASSIERES:

Lycaon or Cynhyène: Lycaon Pictus.

Spotted hyena: Crocuta crocuta.

Jackals: Canis adustus, Canis aureus.

Serval and servalin: Felis serval; Felis brachyura.

Wild cat: Felis libyca (sylvestris).

Golden cat: Felis aurata.

Otters: Lutra maculicollis, Anyx capensis.

Honey badger: Mellivora capensis.

Zorilla: Zorilla striatus.

Civet: Civettictis civetta.

Genettes and Pseudogenette: Genus Gentta, genus Pseudogenetta.

Nandinie: Nandina binotata.

Poïane: Poïana richardsoni.

Mongooses: Genres Herpostes, Ichneumia.

Mangoes and Mungos: Genus Mungos.

PRIMATES:

Bay colobus: Colobus badius.

True colobus or van beneden: Colobus verus.

Cynocephali: Genus Papio.

Patas or red monkey: Erythrocebus patas.

Cercocèbes or Mangabeys: Genus Cercocebus.

Callitriche or green monkey: Cercopithecus aethiops.

Mone: Cercopithecus mona.

Nodding or sealing bar: Cercopithecus.

Diane: Cercopithecus diana.

REPTILES:

Nile crocodile: Crocodylus nilticus.

Gharial-snouted crocodile, Cabinda: Crocodylus oataphractus.

Forest or marsh crocodile: Osteolaemus tretrapis.

Nile Monitor Lizard: Varanus niloticus.

Savannah monitor lizard: Varanus exanthematicus.

Seba Python: Python sebae.

Royal Python: Python regius.

APPENDIX IV: SMALL GAME

List of so-called small game wild animals whose hunting is authorized for customary users and for holders of small hunting licenses and special sporting licenses within the limits of the general latitudes of hunting authorized by law.

MAMMALS:

ANTILOPES:

Guib or Harnaché Mina: Tragelaphus scriptus.

Redunca or Reed Cob: Redunca.

Grimm's duiker or doe pig: Sylvicapra grimmia.

Duiker (with black dorsal stripe): Cephalophus dorsalis.

Maxwell's duiker or gray doe: Philanthomba Maxwelli.

Duiker (red-sided or doe): Cephalophus rufilatus.

Black duiker: Cephalobus niger.

Ourébi or Oribi: Ourebia ourebi.

SUIDES:

Warthog: Phacochoerus aethiopicus.

River hog: Potamochoerus porcus.

DAMANS:

Rock hyrax: Procavia ruficeps.

Tree hyrax: Dendrohyrax dorsalis.

RODENTS:

African hare, improperly called rabbit: Lepus aegypticus.

Grasscutter, improperly called agouti: Aulacodus (syn. Thryonomys), swinderianus.

Porcupine: Hystrix cristata.

Atheruria: Atherura africana.

All squirrels: Genus Xerus, Protexerus, Epixerus, Funisciurus, Heliosciurus.

INSECTIVORES:

White-bellied hedgehog: Atelerix albiventris.

GIIER BIRDS:

Geese, ducks, teals: Order Anseriformes, family Anatidae.

Guinea fowl, francolins, quail, rock hen: Order of Galloformes.

Plovers, lapwings, knights, curlews, Oedicnemes, snipe: among the Chanadriidormes.

REPTLES:

Tortures.

Decree n ° 97-130 of March 07, 1997
regulating the possession of ivories

THE PRESIDENT OF THE REPUBLIC,

On the report of the Minister of Agriculture and Animal Resources, the Minister of Housing, the Life and Environment, the Minister of Culture and the Minister of Commerce

Seen the constitution

Seen the law n ° 65-255 of August 4, 1965 relating to the protection of fauna and the exercise of hunting such as amended by law n ° 94-442 of August 16, 1994

Seen the law n ° 87-806 of July 28, 1987 on the protection of the cultural heritage having regard to Decree No. 94-448 of 25 August 1994 on the accession of the Republic of Côte d'Ivoire to the convention on International Trade in Endangered Species of Wild Fauna and Flora disappearance, signed in Washington on March 3, 1973, together with decree n ° 94-449 of August 25, 1994 on the publication of the said Convention

Seen the decree n ° 96-PR / 002 of January 26, 1996 appointing the members of the Government such as amended by decree n ° 96-PR / 10 of August 10, 1996

Seen the decree n ° 96-179 of March 1st, 1996 relating to the attributions of the members of the Government

The Council of Ministers heard

DECREED

CHAPTER I: DEFINITIONS

Article 1 - Under the terms of this decree are "ivories"

- elephant tusks

   - in raw state
   - Worked
   - Incorporated into other objects
- ivory objects
- objects of which ivory is the main component in quantity or value.

Article 2 - Collector of Ivories within the meaning of this decree is any person, public or private, owner of one or more legally owned Ivories.

Article 3 -Ivories owned by the State and public authorities whether or not they are kept in national or regional public museums constitute the National Collection of Ivories.

Article 4 -The National Collection of Ivories of Côte d'Ivoire and the private collections of Ivoires constitute "the Eburnean Collection of Ivories" hereinafter referred to by the acronym "Ivoires CI".

CHAPTER II: CONSTITUTION AND NATURE OF CI IVORIES

Article 5 - Ivories held on national territory must be declared to the Water Administration and Forests by filing an identification sheet in accordance with the annex to this decree.

Certificates of origin issued by the Water and Forestry Administration in application of Decree No. 66-425 above do not exempt their owners from the declaration provided for above.

Each declared ivory is entered in a special register called the "Ivory Directory" and has a ID number.

An extract from the Directory is issued to each Collector as far as he is concerned.

The extract has the value of a certificate of origin and constitutes a legal presumption of ownership in favor of the collector he mentions.

Article 6 - Ivoire-CI is part of the national cultural capital. As such, the Ivories that compose it are national property notwithstanding the quality of the persons of which they are the property.

CHAPTER III: RIGHTS AND OBLIGATIONS OF IVORY COLLECTORS

Article 7 - Any holder of ivories has the obligation to make the declaration as stated in article 5 above.

Article 8 - Declared and listed ivories may be:

- a permanent or exceptional exhibition
- a free or expensive transfer

In the latter case, the transfer to the Ivory Directory is mandatory and must be done without

deadlines.

Article 9 - Any collector of Ivory can freely modify the medium or integrate it into another object under reserve to preserve its entirety.

Processing of ivory is subject to joint authorization from the Minister of Water and Forests and the Minister in charge of Culture.

CHAPTER IV: EXIT OF IVORIES FROM THE NATIONAL TERRITORY

Article 10 - The exit of Ivories from the national territory is prohibited except exceptional authorization issued by the Minister in charge of Water and Forests.

Only natural persons can request such authorization on a strictly personal basis. having legally resided in Côte d'Ivoire for a minimum uninterrupted period of six months.

The authorization can only relate to ivories registered in the Directory of Ivories in the name of the applicant.

CHAPTER V: INTRODUCTION OF IVOIRES INTO NATIONAL TERRITORY

Article 11 - The importation of ivories may be authorized by the Minister in charge of Water and Forests in view of a express and explicit authorization from the competent authorities of the country of origin. This authorization must be obtained prior to boarding the Ivories. It can only be granted to a natural person as personal items.

Ivories concerned by the authorization must be declared on their entry into the national territory for lack of which they constitute a prohibited goods seizable at any time.

CHAPTER VI: MARKETING OF IVORIES

Article 12 - The trade in ivories is prohibited.

However, traders regularly installed, with permanent open infrastructure to customers and trading in ivories prior to the date of publication of this decree can continue this activity provided:

- to have made themselves known to the Water and Forests Administration
- to have duly registered their Ivories.

Until this double condition is satisfied, the exhibition of their ivories will only be possible under reservation to clearly state: "these goods are not offered for sale".

CHAPTER VII: OFFENSES AND SANCTIONS

Article 13 - Any holder of an unlisted ivory is, by application of Article 32 of Law No. 65-255 above, presumed guilty of an offense against the provisions of this law and prosecuted under the same conditions only if the criminal act had actually been noted.

The exhibitor is prosecuted in the same way, even temporarily of an unlisted ivory.

Article 14 - Without prejudice to the penalties provided for in Article 13 above, any transaction having as its object a Unlisted ivory, any attempt to export an ivory, any attempt to import ivories by fraud to the provisions of this decree is liable to the penalties provided for by law n ° 87-806 of July 28, 1987 above.

CHAPTER VIII: TRANSITIONAL MEASURES

Article 15 - Public or private persons holding Ivories on the date of publication of this decree have a period of two months to make a declaration to the Water and Forests Administration either at the Directorate of Nature Protection in Abidjan or to the Head of the Forest Region of the domicile of the declaring.

CHAPTER IX: FINAL PROVISIONS

Article 16 - This decree cancels all previous contrary provisions.

Article 17 - The Minister of Agriculture and Animal Resources, the Minister of Culture, the Minister of Commerce and the Minister of Economy and Finance are each responsible for the execution of this decree which will be published in the Official Gazette of the Republic of Côte d'Ivoire.

Done in Abidjan, March 07, 1997

Henri Konan BEDIE

Decree n ° 66-423 of September 15, 1966
fixing the hunting license regime and the terms of their attributions in Republic of Côte d'Ivoire

THE PRESIDENT OF THE REPUBLIC,

On the proposal of the Minister for Agriculture,

Seen the law n ° 65-255 of August 4, 1965, relating to the protection of fauna and the exercise of hunting and in particular articles 9 and 10 thereof;

Having heard the Council of Ministers,

DECREED:

First article :

The hunting license regime provided for in Article 9 of the Law on the Protection of Wildlife and the Exercise of hunting is set as follows:

TITLE ONE

SMALL HUNTING LICENSE FOR MILKING WEAPONS

Article 2:

The small hunting license for a trafficking weapon can only be issued to persons aged at least twenty and one year old, holders of a permit to carry a trafficking weapon.

The validity period is limited to the current hunting season. It is issued by the sub-prefects who can delegate their powers to the competent representatives of the Ministry of Agriculture.

It gives its holder, within the framework of hunting regulations, the right to shoot only in the sub-prefecture. of his residence, the animals listed in appendices III and IV of the law on the protection of wildlife and the practice of hunting.

This hunting of trafficking weapons will be prohibited in the wildlife management zones provided for in Article 19 of the above-mentioned law.

NATIONAL PERMIT

Article 3:

It can only be issued to people who are at least twenty-one years old, holding a license to carry weapons perfected.

Its validity is limited to the current hunting season. It is issued by the sub-prefect who can delegate its powers to the competent representative of the Ministry of Agriculture.

In fully-fledged municipalities, national permits are issued by the prefect who can also delegate its powers to the competent representative of the Ministry of Agriculture.

Any national license gives the right to shoot throughout the territory the animals of Annexes II, III, IV in the limits provided for in these appendices.

TITLE II
SPORTS HUNTING LICENSE
MEDIUM HUNTING LICENSE

Article 4:

It can only be issued to people who are at least twenty-one years old, holding a license to carry weapons Advanced, rifled with a caliber of 7 millimeters or greater.

The validity period is limited to the current hunting season. It is issued by the director of the competent services of the Ministry of Agriculture.

It gives its holder, within the framework of the hunting regulations, the right to shoot throughout the territory, except in wildlife management zones;

1° Animals in Annexes II., III and IV within the limits provided for in these annexes without slaughter taxes;

2° partially protected animals;

Annex I, class C within the limits indicated below and against payment, after slaughtering, of a tax including the amount per animal slaughtered is set by order of the Minister for Agriculture taken on the advice of the Minister for economic and financial affairs.

Total number of animals whose slaughter
is authorized by an average hunting license

Species :	
Elephant	1
Buffalo	1
Hippopotamus	1
Hippotrague	1
Cob de Buffon	1
Cob defassa	1
Bubale	1
Leopard	1
Lion	1

PASSENGER PERMIT

Article 5:

It can only be issued to people who are at least 21 years old, holding a permit to carry a striped weapon of a caliber greater than 7 millimeters or capable of justifying the rental of a rifled weapon in accordance with current regulations.

It is issued by the director of the competent services of the Ministry of Agriculture and valid for one month.

It gives its holder, within the framework of the hunting regulations, the right to shoot on the entire territory:

1° The animals listed in Annexes II, III, IV within the limits provided for in these annexes without slaughter tax;

2° Partially protected animals in Annex I, class C, within the limits indicated below and in return for payment after slaughter of a tax, the amount of which per animal slaughtered is

fixed by order of Minister Delegate for Agriculture taken on the advice of the Minister Delegate for Economic and Financial Affairs.

Total number of animals whose slaughter
is permitted by passenger permit

Species:

Elephant ………………………………………………………………………..…… 1

Buffalo……………………………………………………………………………… 1

Hippopotamus…………………………………………………………….………… 1

Bongo …………………………………………………………………….…………… 1

Situtunga ……………………………………………………………………………….. 1

Hippotrague ………………………………………………………………………… .. 1

Cob defassa ………………………………………………………………… 1

Cob de Buffon ………………………………………………………………….... 1

Bubale ……………………………………………………………………………… 1

Leopard or panther ……………………………………………………...…… .. 1

Leo ………………………………………………………………………….……… 1

3° Partially protected animals, annex I, class B, on a unit basis and without slaughter tax except
for the master colobus, the yellow-backed duiker and the alcoholic beetle.

BIG HUNTING LICENSE

Article 6:

It can only be issued to persons aged at least 21 years old, holders of a permit to carry weapons rifled with a caliber greater than 7 millimeters.

Its validity is limited to the current hunting season. It is issued by the director of services officials of the Ministry of Agriculture.

It gives its holder, within the framework of the hunting regulations, the right to shoot on the entire territory:

1° The animals listed in Annexes II, III, IV within the limits provided for in these annexes without slaughter tax;

2° Partially protected animals, appendix I, class C, within the limits indicated below and with payment after slaughter of a tax, the amount of which per animal slaughtered is set by order of the Minister Delegate to Agriculture taken on the advice of the Minister for Economic and Financial Affairs.

Total number of animals authorized for slaughter
by big game hunting license

Species:

Elephant ……………………………………………………………. …… 2

Buffalo……………………………………………………………. ………. 2

Hippopotamus……………………………………………………………………………. 1

Bongo ………………………………………………………………. ……. 2

Situtunga ……………………………………………………………. …………. 2

Hippotrague ………………………………………………………………………… .. 2

Cob defassa ……………………………………………………………………. 2

Bubale ……………………………………………………………………………………. 2

Cob de Buffon ………………………………………………………………………. ... 3

Leopard or panther ……………………………………………………………. … .. 2

Leo ………………………………………………………………………………………. … 1

3° Partially protected animals, annex I, class B, on a unit basis without slaughter tax except for:

- Magistrate Colobus;
- Yellow-backed duiker;
- Hylochère.

TITLE III

SPECIAL PERMIT FOR COMMERCIAL CAPTURE

Article 7:

It can only be issued to a person or a company that has, from a technical point of view, all guarantees deemed necessary and sufficient to engage in this type of activity and holder of a commercial license.

It is granted by the Minister for Agriculture and is valid for the current year. Mention is made authorized means of capture.

As regards fully protected animals, commercial capture permits cannot be granted only to holders of scientific catch permits under the conditions provided for in Article 8.

These permits specify exactly the rights conferred on its holder and the perimeter in which they can practice.

Holders of fully and partially protected commercial capture permits are obliged to keep a catch booklet which will be presented along with the license, at any request from the agents authorized for this purpose and in which will be recorded on a day-to-day basis, the protected animals they will have captured, incidentally slaughtered or acquired within the limits allowed by their license.

Mention will be made in this notebook of the species of the captured animal, its sex, characteristics allowing its identification, the circumstances of the capture, in particular the date and place of issue of the certificate of origin then, in case of export, the date and point of exit, the health visa, the visa of competent department of the Ministry of Agriculture controlling the export of protected animals and the visa of the customs noting the exit.

In the event of sale on site or donation of a protected animal to a government zoo, the transfer or the donation will be mentioned on the catch book instead of the export.

The holder of the commercial capture license will be authorized to hold until their sale or export, animals regularly acquired during the validity of the license and duly entered in the catch book if it is of protected animals.

These animals must be maintained in a good state of care and hygiene.

Capture permits do not give any of the rights equivalent to a hunting permit and do not allow the use of firearms.

In addition to the commercial license and the commercial capture license, the beneficiary may be subject to payment of duties and taxes fixed annually.

TITLE IV
SCIENTIFIC HUNTING OR CAPTURE PERMIT

Article 8:

They are issued by the Minister for Agriculture.

The permit application must indicate the name and capacity of the beneficiary and the holder, the reasons given, the number of animals of each species fully protected whose slaughter or capture is requested.

The permit specifies exactly the period of validity, the rights conferred on its holder and the perimeter in which he can practice.

TITLE V
OBLGATIONS OF PERMIT HOLDERS

Article 9:

The permits are essentially personal; they cannot be transferred or sold.

It cannot be issued to the same person as one license in the same year. However, it can be granted, during the period of validity of a license, a license of a higher category, with the payment of the price difference between the two permits. The total slaughter latitudes thus granted shall not may never exceed the total of those provided for in the license of the highest category.

Anyone wishing to obtain a permit must address to the sub-prefect of their place of residence or to the prefect, in the case provided for in Article 3, a request indicating or including:

1° His name, first name, date and place of birth, nationality, domicile and profession;

2 ° Nature of the permit;

3 ° A declaration certifying that the person concerned has never been convicted of a hunting offense or indicating, if applicable, the date, place and nature of the conviction.

To this request must be attached:

1 ° The receipt for the fee relating to the requested permit;
2 ° two typical "identity photo" photographs;
3 ° The applicant's license to carry weapons;
4 ° If applicable, the previous permit obtained.

The sub-prefect rules with regard to hunting permits for milking and small hunting weapons and forwards other requests with notice to the qualified authority.

No permit may be issued without the application having been filed, investigated, endorsed and transmitted in these conditions.

Their issuance may be refused by the competent administrative authority.

In the event of loss of the permit, a declaration must be made by the person concerned, to the sub-prefecture of his place usual place of residence or at the prefecture in the case referred to in Article 3. A duplicate may be issued subject to payment of a special tax set at tenth of the related fee provided for the permit corresponding.

ROYALTY FEE

Article 10:

The fees provided for on the occasion of the issuance of permits and duplicates and slaughter taxes are set by order of the Minister for Agriculture taken on the advice of the Minister for Business economic and financial.

PERMIT ADVERTISING

Article 11:

The publication of scientific permits and commercial capture permits will be made in the Official Journal, with indication of the names and qualities of the permit holders, of the nature and validity of these.

FORFEITURE OF PERMITS

Article 12:

The publication of the forfeiture or deprivation of the granting of hunting permits or capture permits will be made in the Official Journal, indicating the names and qualities of the license holders, the nature and validity of these.

In addition, anyone who has obtained a hunting license by deceiving the good faith of the administrative authority, although he has been stripped of his rights and who will be convicted of fraud, will see the new license confiscated and, if he hunted under cover, will be considered again in contravention of the provisions of the this decree.

HUNTING LOG

Article 13:

Holders of any license other than the license for a milking weapon and the small hunting license nationals are obliged to keep a hunting log which will be presented together with the license, at any requisition of the agents of the authority and where the animals that they have slaughtered will be registered on a daily basis within the limits authorized by the latitudes of slaughter associated with each type of permit.

For each animal will be specified:

- the date and place of slaughter (or capture) and sex;
- for elephants, the weight, the length of the outer curve, the base circumference of each point.

Any animal captured alive as a result of fortuitous circumstances counts, whatever its age, as an animal kill.

Slaughter taxes must be paid as quickly as possible, within the limit of fifteen clear days after slaughter, on pain of confiscation of trophies and remains and the weapon used in slaughter.

Taxes will be paid on presentation of the hunting license to the sub-prefecture of the place of slaughter.

In the event of force majeure, payment may take place at the sub-prefecture of the hunter's residence. This the latter must then inform the authorities of the sub-prefecture where the slaughtering took place, of the payment of the tax indicating the number, date and amount of the receipt.

The amount of the collection, the number, the date and the place of issue of the receipt must be mentioned on the hunting log by the tax collector.

INJURED ANIMALS

Article 14:

Anyone who has injured an animal is required to do everything possible to find and finish it, even if it is an animal she did not have the authorization to hunt, with the exception of the pursuit in a wildlife reserve where he would have taken refuge. In the latter case, it must make a declaration details of the authority responsible for managing the reserve.

The injured animal must be entered on the hunting log the same day with the mention "injured". If two animals of the same species have been injured, the species's slaughter latitude is reduced by one.

If the injured animal is an elephant, buffalo, lion or leopard and has not been found or killed in a within twenty-four hours after the time he was injured, a full statement must be made immediately to the nearest administrative authority.

TITLE VI

OFFENSES AND PENALTIES

Article 15:

Violations of the provisions of this decree are sought, noted, repressed and repaired in accordance with the general rules in force and provided for by the law on the protection of wildlife and the exercise of hunting.

Article 16:

The Minister for Agriculture, the Minister for the Armed Forces and Civic Service and the Minister for the Interior are responsible, each as far as he is concerned, with the application of this decree which will be published in Official Journal of the Republic.

Done in Abidjan, September 15, 1966.

FELIX HOUPHOUËT-BOIGNY.

Decree n ° 66-424 of September 15, 1966
relating to the hunting guide license

THE PRESIDENT OF THE REPUBLIC,

On the proposal of the Minister for Agriculture,

Seen to law No. 65-255 of August 4, 1965, relating to the protection of wildlife and the practice of hunting, in especially its 13 and 14;

Having heard the Council of Ministers,

DECREED:

First article :

Acts as a hunting guide anyone who hires his own services, directly or through a employer, as principal or accessory, to lead or accompany a hunting expedition in order to make others benefit from their hunting knowledge and protect them from the dangers they might incur.

Article 2:

No one may act as a hunting guide in the territory of the Republic of Côte d'Ivoire, whether usual or occasional, if not the holder of the corresponding special license.

Article 3:

The only candidates who can apply for a hunting guide license are those who fulfill the following conditions:

- Be a citizen of Ivory Coast or, for foreigners, resided for at least three years in Ivory Coast or in a neighboring state;
- Be at least twenty-one years old;
- Not having incurred any penalty that could result in the loss of civic rights.

Registration requests, accompanied by the corresponding supporting documents and indicating the names, first names, profession, nationality and domicile of the candidates, must be received before December 1 of each year at the Ministry of Agriculture.

The latter draws up by decree taking effect on January 1, the list of registered candidates.

Article 4:

All candidates must complete a period of apprenticeship during a hunting season from the date of its registration.

During this period, notwithstanding the provisions of Article 2 above and subject to the declaration prior to being made to the heads of the forestry inspectorates concerned, the candidate may accompany hunting expeditions as an apprentice, under the responsibility and in the company of a hunting guide dismissed. The presence of the latter being however obligatory at his side only for research and pursuit of the following animals: elephants, buffaloes, lions, leopards. The statements mentioned above must be covered by the employer's hunting guide which is also required, after each expedition, to issue a circumstantial certificate to his apprentice.

During the same period, the candidate may also, at his request, possibly be entrusted with as part of his apprenticeship, the execution of destruction hunts or various hunting activities under the direction and control of the relevant department of the Ministry of Agriculture.

Article 5:

After completing their apprenticeship period, each candidate must undergo an examination by a committee composed as follows:

President :

The Minister for Agriculture or his representative.

Members :

- The director of the competent services of the Ministry of Agriculture or his representative;
- The director of the Tourist Office or his representative;
- A licensed hunting guide or, failing that, a representative of a profession with activities in relationship with hunting tourism.

This committee meets when convened by its Chairman. It can, at the behest of its president, do call for outside examiners; these have only an advisory role.

During the deliberations and in the event of an equal vote, that of the president is preponderant.

Article 6:

The exam includes a theoretical test, a practical test and an assessment of the activities of the candidate during his apprenticeship period.

a) Compulsory subjects	Coefficient
Simple notions of zoology, ecology of wild animals, hunting	2
Regulations on hunting and wildlife protection	3
Shooting test	3
Weapons and ammunition, relevant regulations	1
Geography of hunting regions	1
Hygiene, prophylaxis, routine and emergency care	1
**b) Optional subjects**	**Coefficient**
Foreign languages (English, German, Spanish)	2
Common vernacular languages	2
**The practical test includes:**	**Coefficient**
Breakdown of a vehicle	2
Target shooting	1
The assessment of the candidate's activities during his apprenticeship period is made in view of his hunting logs, certificates mentioned in article 4 above and technical reports written by the competent services of the Ministry of	4

Agriculture and referred to by the Tourist Office

Article 7:

Each subject examined gives rise to the attribution of a numerical mark between 0 and 10.

less than 3 awarded in a compulsory subject is eliminatory. The minimum number of points that must total number of candidates for the license of hunting guide is 105.

The results of the tests are recorded in a report. All applicants who are not likely to obtain a hunting guide license, may be authorized to extend their apprenticeship during a new hunting season.

Article 8:

The hunting guide license is granted by order of the Minister for Agriculture. This license is final, except disciplinary sanction of withdrawal taken in application of Article 15 below.

Article 9:

Notwithstanding the provisions of the preceding articles and on a transitional basis, the Minister for Agriculture may grant a hunting guide license to people with solid hunting experience and having already exercised this profession in Côte d'Ivoire prior to the publication of this decree.

Candidates who meet only the first of these conditions will be subject to the examination but may be exempt from the apprenticeship provided for in Article 4.

This transitional period will be brought to an end by the decree of the Minister for Agriculture.

Article 10:

Hunting guides have the strict obligation to:

- Ensure that their customers observe the regulations in force in terms of hunting and protection of wildlife ;
- Protect their customers against all risks inherent in hunting;
- Finish off injured animals;
- Apart from the two cases mentioned above, only shoot with the express consent of their clients ;

- Maintain the sporting character of hunting in all circumstances;
- Always behave correctly and behave correctly with regard to customers, employees and populations encountered.

Article 11:

Any hunting guide is required to contract with an approved insurance company, prior to any hunting expedition, insurance covering full civil liability and that of apprentice guides and the staff he employs for any accident or damage that may occur to his customers or third parties during shipment.

In the event of an accident involving a client or a third party caused by a client, the hunting guide must notify immediately the nearest administrative authority which immediately carries out an investigation.

Article 12:

Hunting guides must declare each expedition they are called upon to lead or accompany to the head of the Forest Inspectorate of the initial place of departure or the point of entry into Ivory Coast of the expedition.

Except in cases of force majeure, the proof of which lies with the guide concerned, this declaration must reach destination at least fifteen days before the date scheduled for the start of the shipment. She specifies, in addition to the names of the guide and his clients, the dates and places planned for the start and end of the expedition or, possibly, its entry into and exit from Ivorian territory.

Article 13:

Hunting guides cannot lead or accompany a hunting expedition without being equipped with big game hunting license.

However, it is forbidden to use these permits to increase the latitudes in any way. slaughter of their customers and that a guide is required to complete must be registered in the account and on the client's hunting log.

Article 14:

Hunting guides are civilly liable for breaches of the regulations for the protection of the wildlife and hunting practice by their clients during the hunting expeditions they lead or

accompany. They can however be exempted from this civil liability under the conditions provided for in Article 26 of the law regulating the protection of wildlife and the practice of hunting.

The hunting guides remain always and in all circumstances responsible for the payment in Coast Ivory slaughter taxes that may be due for animals killed by their customers in the territory of

Republic.

Article 15:

Regardless of responsibilities as well as legal proceedings and sanctions they may possibly incur, hunting guides are liable to disciplinary sanctions for breaches of regulations in force in matters of hunting and wildlife protection, neglect or non-observance of rules published in articles 9, 10, 11 and 12 above, common law offense or notorious incompetence.

These sanctions are, in order of increasing severity:

- The warning;
- The blame ;
- The withdrawal of the license for a fixed period;
- The final withdrawal of the license.

They are pronounced by decree of the Minister for Agriculture, the first two directly, the others after consultation and on the proposal of the committee provided for in Article 5.

This commission draws up its proposals on the basis of the documents submitted to it and the explanations or justifications that the guide in question must have been invited to give in writing on the facts that are criticized.

Article 16:

Breaches of the provisions of this decree are punished in accordance with law n ° 65-255 of August 4 1965.

Article 17:

Subject to reciprocal agreement, hunting guide licenses issued in neighboring states may be valid in the Republic of Côte d'Ivoire.

Article 18:

The Minister for Agriculture, the Minister of the Interior, the Minister of the Armed Forces and the Service civic and the Minister in charge of Tourism are responsible, each as far as it is concerned, for the application of the this decree which will be published in the Official Journal of the Republic of Côte d'Ivoire.

Done in Abidjan, September 15, 1966.

FELIX HOUPHOUËT-BOIGNY.

Decree n ° 66-425 of September 15, 1966
regulating the traffic, movement, import, exploitation of animal trophies
protected and spectacular and their remains

THE PRESIDENT OF THE REPUBLIC,

On the proposal of the Minister for Agriculture,

Seen the law n ° 65-255 of August 4, 1965, relating to the protection of fauna and the exercise of hunting and in particular its Articles 15;

Having heard the Council of Ministers,

DECREED:

EXCAVATIONS AND TROPHIES

First article :

Holders of hunting permits giving the right to the slaughter of animals mentioned in Annexes I and II of the Law on the protection of wildlife and the practice of hunting as well as the holders of special capture permits and Scientists can freely dispose of the remains of animals regularly slaughtered or captured by them.

The circulation of the remains of fully protected animals (except for holders of a scientific permit specifically mentioning these animals), is strictly prohibited and gives rise to seizure.

CERTIFICATE OF ORIGIN

Article 2:

No partially protected animal, dead or alive, no trophy or remains of these animals may be detained, moving or being exported from Ivory Coast without being accompanied by a certificate of origin allowing its identification.

Certificates are issued by the competent services of the Ministry of Agriculture or failing that, by the chefs administrative districts.

Elephant points must bear indelible mentions:

a) The license number followed by the letters A or B to differentiate the points, followed by the last two digits of the year of issue of the permit (eg: 37 to 65);
b) The weight of the point.

These particulars as well as the external curves and the circumference at the base of the points must appear on the certificate of origin and on the hunting log.

The certificates of origin must mention the numbers, date, amount and place of payment of taxes slaughter when scheduled. An amplification of each certificate of origin must be addressed to the competent services of the Ministry of Agriculture.

With regard to live animals, trophies or remains from a territory will be issued by the administrative post or by the Ivorian border customs post, upon production of a document from foreign authorities and justifying the legitimacy of the possession of animals, trophies or remains. Mention of this part will be made on the certificate of origin.

MASSACRES FOUND

Article 3:

It is forbidden to appropriate:

- Ivory from elephants found dead;
- The massacres and trophies of protected animals proven dead.

These remains must be returned to the first administrative post reached.

They are sent to the receiver of Domains who, after advertising, proceeds to the auctionpublic for the benefit of the state budget.

Article 4:

Those who hand over found elephant tusks to the administrative authority will receive a premium per kilogram, the amount of which will be set by order of the Minister for Agriculture, taken on the advice of Minister for Economic and Financial Affairs.

A report will immediately be drawn up by the administrative authority which will receive the ivory in deposit. This document shall indicate the name of the depositor as well as the date, place and circumstances of the find, so as precise as possible, the weight and length of each tusk as well as the registration number in the ivory deposit register of the administrative district.

A copy of this report will always be sent to the local service as soon as possible responsible for the Ministry of Agriculture.

SEIZURES SEIZED

Article 5:

The ivory and the remains are sent to the Receiver of Domains and sold at public auctionfrom confiscation or seizure for breach of hunting regulations.

The Receiver of Domains draws up an extract from the public auction minutes to the Prefect.

Article 6:

Violations of the provisions of this decree are sought, noted, repressed and repaired in accordance with the general rules in force and provided for by the law on the protection of wildlife and the exercise of hunting.

Article 7:

The Minister for Agriculture, the Minister for the Armed Forces and Civic Service and the Minister for the Interior are responsible, each with regard to the execution of this decree which will be published in official Journal of the Republic.

Done in Abidjan, September 15, 1966.

FELIX HOUPHOUËT-BOIGNY.

Decree n ° 66-433 of September 15, 1966
on the status and regulations of the classification procedure for nature reserves, full or partial and national parks

THE PRESIDENT OF THE REPUBLIC,

On the report of the Minister for Agriculture,

Seen the law n ° 65-255 of August 4, 1965, relating to the protection of fauna and the exercise of hunting in in particular in Articles 4 and 5;

Seen the law n ° 65-425 of December 20, 1965, relating to the Forest Code;

Having heard the Council of Ministers,

DECREED:

First article :

The integral nature reserves are part of the classified forest domain of the Republic of Côte d'Ivoire.

The following are strictly prohibited over their entire extent:

- Any forestry, agricultural or mining exploitation; - All excavations or prospecting, soundings, earthworks or constructions and generally all works tending to modify the appearance of the land or the vegetation;
- Any act likely to harm or disturb the flora or fauna.

Strict nature reserves are free from any right of use.

Penetration, traffic including by air at altitudes below 200 meters, the camping in the Strict Nature Reserves is strictly prohibited, except for the following people;

1 ° Without special authorization, officials and agents of the competent services of the Ministry of Agriculture specially responsible for the conservation of these reserves and the staff under their orders and accompanying them. These officials may use firearms for their own self-defense and that of the people they escort;

2 ° On written authorization issued by the Minister for Agriculture or his delegate, personalities scientific for strictly scientific purposes and under the escort designated in the authorization.

The authorization may provide for the collection of mineral samples that should not apparently modify the places ;

- The collections of botanical samples should be limited to the organs necessary for the identification of cash;
- Catches of animals may in no case give rise to the use of firearms.

PARTIAL NATURAL RESERVES

Article 2:

With a view to nature conservation, certain areas may be subject to restrictions on hunting, the nature of the animals, the use of plants or the installation of buildings.

Partial reserves include:

- Scientific reserves, such as botanical, zoological or paleontological;
- Reserves of a tourist or climatic nature;
- Natural sources of hydroelectric energy.

These reserves are part of the classified forest domain.

NATIONAL PARKS

Article 3:

National parks are part of the classified forest domain. They are devoted to the propagation, to the protection of animal life and wild vegetation, the conservation of objects of aesthetic interest, geological, historical or scientific for the benefit of the public, for its education and its recreation.

Direction, management and supervision are entrusted to the competent services of the Ministry of Agriculture.

National parks are free from any right of use. Hunting, fishing, or the capture of all animals, the removal of plant species or any objects, the exploitation or mining prospecting.

The rules published in the previous article 1 apply to penetration, to traffic including by road aerial and scientific research in national parks.

However, national parks may also be open to the public under the following conditions:

- Effective control of entries and exits;
- Vehicle circulation limited to roads and tracks open to the public;
- Foot traffic, photographic and cinematographic hunting only under escort and limited to certain sectors;
- Night driving by any means whatsoever is prohibited except on certain roads of general interest;
- Daytime parking at locations indicated by security staff;
- Night camp at the sites reserved for this purpose;
- Prohibition of carrying weapons. Those with which visitors may find themselves equipped must, before the entrance to the park be dismantled and put in the cases. Declaration must be made to the post of control and the supervisor can affix the seals.

For each national park, internal regulations will specify the terms of application of this article.

In national parks, the competent services of the Ministry of Agriculture may undertake all works and arrangements necessary for their equipment for scientific, educational and tourism.

People wishing to visit a national park must be in possession of a visit permit issued by the competent departments of the Ministry of Agriculture.

WILDLIFE RESERVE

Article 4:

In zoological reserves "Wildlife reserves", any act of hunting is strictly prohibited except in the case of self-defense or protection of people and property.

In wildlife reserves, the exercise of the right to fish, graze, and harvest of honey, wax and wild fruits for rights holders.

If applicable, for each wildlife reserve, internal regulations specify the conditions for entry, traffic and camping.

Air traffic at an altitude below 200 meters is still prohibited.

In the developed wildlife reserves, a visit permit issued by the competent services and the payment of a royalty may be required.

PROCEDURE FOR CLASSIFICATION OF INTEGRAL NATURAL RESERVES AND PARTIALS AND NATIONAL PARKS

Article 5:

The procedures for classifying natural, integral and partial reserves and national parks are

fixed as follows:

The preliminary projects emanating either from the prefects or sub-prefects, or from the mayors, or from the technical services of the

Ministry of Agriculture must be sent before any other procedure to the Ministry responsible for Agriculture.

Each project must provide the following details concerning the envisaged reserves.

1 ° Purpose, duration, Species which will be protected there (for wildlife reserves). Limit ;

2 ° Inventory of user rights exercising within protected limits, accompanied by proposals tending:

- Or the pure and simple recognition of their full exercise;
- Either on their abandonment, limitation, confinement or redemption;

3 ° Inventory of rights, other than user rights which could be imposed on the land to be reserved, accompanied by proposals for:

- Or the pure and simple recognition of their full exercise;
- Or their amicable redemption;

4 ° Conditions under which, within the protected limits, the installation of new villages or the granting of any concessions or other rights of occupation;

5 ° Traffic and parking conditions.

The project is subject to the approval of the Minister for Agriculture who, after approval, brings it to theknowledge of the public by all means of regulatory advertising and by display, for a month, at chief towns of the prefectures and sub-prefectures concerned.

After this posting deadline, proven by certificates from the interested prefects and sub-prefects, if no No dispute arose, the project is submitted to the Council of Ministers and the reserve created by decree.

If disputes have been raised by people who have been able to object, the Minister responsible for agriculture appoints a commission chaired by the prefect and comprising two deputies from the constituency and the representative of the Minister for Agriculture.

This commission is responsible for studying under what conditions may be abrogated, limited, confined or bought back the rights of use and possibly to settle the disputes amicably. This commission must necessarily hear all the village heads and local communities concerned. It is transported on site for this purpose. It establishes on a report of its debates which is attached to the project submitted to the Council of Ministers.

People who have rights, other than user rights, to assert, may file an opposition during the project posting deadlines; as well as during the thirty days following arrival at the administrative centers of the interested prefectures of the Official Journal containing the classification decree. The oppositions will be recorded to date in the prefectural capitals.

The disputes may be settled amicably by the submission provided for above, otherwise the opponents will have to bring their claims before the competent courts.

DECLARATION OF INTEGRAL AND PARTIAL NATURAL RESERVES AND NATIONAL PARKS.

Article 6:

Projects for the total or partial decommissioning or modification of the statutes of integral nature reserves and partial and national parks are studied and formatted as classification projects, then forwarded to the Council of Ministers.

Article 7:

Violations of the provisions of this decree are sought, noted, repressed and repaired in accordance with the general rules in force and provided for by the law on the protection of wildlife and the exercise of hunting and the forest code.

Article 8:

This decree will be published in the Official Journal of the Republic.

Done in Abidjan, September 15, 1966.

FELIX HOUPHOUËT-BOIGNY.

Order n ° 1712 AGRI / EFC of December 29, 1966
setting the conditions for the elimination or removal of pests

THE DELEGATE MINISTER OF AGRICULTURE,

Seen the law n ° 65-255 of August 4, 1965, relating to the protection of fauna and the exercise of hunting, in in particular in Articles 20 and 21,

STOPPED :

DESTRUCTION HUNT

First article :

In the event that certain animals, whether protected or not, constitute a danger to human life or cause damage to crops or livestock, permits for individual hunting or beating will be granted by the Minister for Agriculture after an on-site investigation by the head of the forest inspectorate of spring.

In cases of absolute urgency, the prefects may grant these authorizations. They will then have to report it immediately to the Minister for Agriculture.

By way of derogation from the preceding provisions, hunts or beatings of fully protected animals cannot be can only be authorized by the Minister for Agriculture.

Destruction hunts must be seriously motivated. They are exceptional temporary. The hunting events which they make possible are subject to close control by officials of the Administration.

Unless there are exceptions to be justified, this destruction carried out by means of individual rifle hunting; the beaten ones As a last resort to use only when individual hunts are not possible or have failed.

As far as possible, the hunts will be entrusted to the Water, Forestry and Hunting agents or, failing that, the sub-prefects who requested the authorization for destruction, must report on hunts and hunts in the shortest possible time.

They will indicate the detailed hunting motives and the beatings, the names and qualities of the hunters authorized to slaughterings, the days and places as precise as possible of the hunts or beatings, the weapons used, the accidents, if any, the number, sex and age (adult, juvenile or infant) of animals slaughtered and whether these are elephants, the weight and dimensions of the spikes. The report will be sent directly to the Minister delegate for Agriculture.

The volunteer hunter will be able to register the animals killed in destruction hunting in his hunting log upon payment of slaughter taxes.

Otherwise, the trophies and remains must be returned to the Administration.

The meat of slaughtered animals will be left to the inhabitants and workers of localities having suffered damage.

Article 2:

For the particular case of nomadic herds which would suffer real damage from the facts of the species predators (hyenas in particular), it may be granted annually by the Minister for Agriculture, an authorization for the destruction of these carnivores by appropriate means.

The authorization request must be submitted by the Ministry of Animal Production which will indicate the places where this destruction must be carried out. The local Water, Forests and

Hunting service will always be notified in advance, investigate and give notice. This request will be examined by the management of Water, Forestry and Hunting, which will submit to the Minister for Agriculture with a reasoned opinion.

SELF-DEFENSE

Article 3:

No offense can be raised against anyone who has done an act of hunting unduly, in necessity immediate defense of his own, that of others or that of his own domestic livestock or of his own harvest. However, the prior provocation of animals remains strictly prohibited.

Proof of the case of self-defense must be provided as soon as possible to local officials the Water, Forest and Hunting Administration.

The remains and trophies collected in this case must be returned to the Administration.

In the event of good faith slaughter, by the holder of a big game hunting license or a passenger license, of an elephant with tusks of less than five kilo, its perpetrator will not be considered to have committed an offense, if he makes an administrative statement and behaves in all other respects as in a self-defense.

The slaughtered animal will count for two units in the count of killed animals and the slaughter taxes will be perceived accordingly.

PERMITTED METHOD OF DESTRUCTION

Article 4:

To ensure the protection of their crops and domestic livestock, farmers are allowed to use pits and traps within a kilometer radius of their villages or on their land culture course.

However, these practices are prohibited inside wildlife reserves and national parks.

The skins of panthers possibly captured by these methods, can be freely marketed at the expense of the buyer to report it immediately to the administrative post on closer.

A tax will be collected on this occasion, the amount of which will be fixed annually and issued a certificate of origin identifying the skin and which will be required during all the successive transactions it may make the object.

Article 5:

Infringements of the provisions of this decree are sought, noted, punished and repaired. in accordance with the general rules in force and provided for by the law on wildlife production and exercise of hunting.

Article 6:

The prefects, sub-prefects and the director of Water, Forests and Hunting are responsible, each in what concerns, the application of this decree which will be published in the Official Journal of the Republic.

A. SAWADOGO.

Order n ° 621 AGRI / EFC of May 29, 1967
regulating the destination of hunting products

The Delegate Minister Of Agriculture,

Seen to Law No. 65-255 of August 4, 1965, relating to the protection of wildlife and the practice of hunting, in in particular in its article 16,

STOPPED :

First article :

Hunting license holders can freely dispose of game or animal meat regularly slaughtered by them within the limits of their personal and family consumption of that of their employees accompanying them during the hunt.

The transport of meat is therefore limited to the quantity corresponding to this consumption.

Surplus meat should be left available to users of the land where the slaughter takes place. The part that returns to rights holders is determined by local tradition. These are always validly represented by the village chief or a notable.

Article 2:

The purchase, sale, transfer or exchange of any hunting meat or game, whatever the origin are only allowed on a strictly individual basis and without an intermediary, between the one who legally killed a hunting animal and any person who wishes to obtain meat from this animal for consumption personal or that of his family.

The transport of this meat or game is authorized.

These operations are in any case and for the whole of the territory, prohibited on the markets, in the trade as well as in favor or for the benefit of the civil or military administration, or agricultural enterprises or industrial.

Article 3:

Breaches of the provisions of this decree are sought, noted and punished in accordance with the general rules in force and provided for by the law on the protection of wildlife and the practice of hunting.

Article 4:

The prefects, sub-prefects and the director of Water, Forests and Hunting are responsible, each in what concerns, the application of this decree which will be published in the Official Journal of the Republic.

A. SAWADOGO.

Order n ° 1068 of September 29, 1967
regulating the hunting of crocodiles and monitor lizards for commercial purposes

The Delegate Minister Of Agriculture,

Seen to Law No. 65-255 of August 4, 1965, relating to the protection of wildlife and the practice of hunting, in in particular in its article 4,

STOPPED :

SPECIAL CROCODILE AND VARAN HUNTING PERMIT

First article :

Anyone wishing to engage in the hunting of crocodiles and monitor lizards in order to market their skins, must have a special permit.

This permit is issued by the Director of Water, Forests and Hunting, after compulsory favorable opinion from the head of the Forest Region concerned.

It gives the right to kill the number of crocodiles or monitor lizards specified on the permit, against payment prior to a slaughter tax set at 200 francs per crocodile or monitor lizard.

Article 2:

The destruction, hunting and capture of crocodiles and monitor lizards with skin wide less than 25 centimeters. This width is measured:

- For crocodiles, on the ventral side and refers to the distance between the horny scales on both sides.
- For monitor lizards, over the entire width of the skin.

Article 3:

Crocodiles and monitor lizards, destroyed, captured or killed outside the standards set above, must be returned as soon as possible, to the nearest Administration.

Article 4:

Notwithstanding the provisions of the above articles, during a transitional period of one year, the traders in crocodile skins and monitor lizards will be authorized to buy and export lots whose percentage of skins smaller than the minimum set will not exceed 25%.

Article 5:

The prefects, sub-prefects and the director of Water, Forests and Hunting are responsible, each in what concerns, the application of this decree which will be published in the Official Journal of the Republic.

A. SAWADOGO.

Order n ° 1069 of December 29, 1967
regulating the keeping of live animals by individuals

The Delegate Minister Of Agriculture,

Vu to Law No. 65-255 of August 4, 1965, relating to the protection of wildlife and the practice of hunting, in in particular its article 17,

STOPPED :

FULLY PROTECTED

First article :

The keeping, trade and export of fully protected animals is strictly prohibited except by holders of scientific or capture permits who have received the authorization.

PARTLY PROTECTED ANIMALS AND SPECTACULAR BIRDS

Article 2:

Holders of special sport hunting permits are authorized to hold under their own responsibility, until their license expires and without any other formality, within the maximum limit of two animals at the same time partially protected animals whose slaughter is authorized by their permit.

It is understood that any animal detained must count as a killed animal appearing in the hunting log, however, it is does not give rise to the payment of the slaughter tax.

People who do not hold special sport hunting permits must declare to the Administrative Authority, the partially protected animals that they may be required to collect. They may be authorized, on their responsibility. The validity of authorizations issued by the Director of Eaux, Forêts et Chasse is interrupted by the departure of their beneficiary.

At the expiration of permits or authorizations to keep, the owners of partially protected animals must hand them over to the zoos of the Republic or to the holders of scientific permits or catches authorized to keep animals of the corresponding species. The latter can compensate holders without this compensation constituting a right.

Trade and export of partially protected animals is prohibited, except for holders of scientific permits or capture permits.

However, the export as donation of partially protected animals to a zoological park or of a scientific body may exceptionally be authorized by the Minister for Agriculture. He In this case, it is up to the exporter to provide proof of the donation and to pay the exit duties.

PREDATORY SPECIES AND SMALL GAME

Article 3:

The detention by private individuals of unprotected animals under their own responsibility is authorized without formality.

The export and trade of unprotected animals is possible under the following conditions:

- Non-commercial export by individuals of unprotected animals, previously regularly held by them, with the authorization of the Director of Water, Forests and Hunting, under reservation of payment of exit fees and compliance with current health regulations;
- Transfer for remuneration of unprotected animals held by individuals to the sole holders of scientific or capture permits and zoos in the Republic.

Article 4:

The annual fees for keeping wild animals in captivity provided for in Article 77 of the Law on protection of wildlife and the practice of hunting will be fixed by joint order of the Minister for Economic and Financial Affairs and the Minister for Agriculture.

Article 5:

Breaches of the provisions of this decree are sought, noted and punished in accordance with the general rules in force and provided for by the law on the protection of wildlife and the practice of hunting.

Article 6:

This decree will be published in the Official Journal of the Republic.

A. SAWADOGO.

Order No. 68 of 23 January 1967 fixing the tariffs of taxes and royalties on hunting and capture of wild animals

The Delegate Minister Of Agriculture,

Seen to Law No. 65-255 of August 4, 1965, relating to the protection of wildlife and the practice of hunting, in in particular Articles 9, 10 and 17;

Seen the decree n ° 66-423 of September 15, 1966 fixing the hunting license regime and the terms of their allocation in Côte d'Ivoire, in particular Article 10,

STOPPED :

First article :

The fees collected in application of the hunting regulations on the occasion of the issuance of permits and slaughter taxes and additional capture rights are set as follows:

A - HUNTING LICENSE

Small game license for

Milking weapon	1,000 FCFA
National small hunting permit	1,500 FCFA
Medium hunting license	5,000 FCFA
Passenger permit	10,000 FCFA
Big game hunting license	15,000 FCFA

B - SLAUGHTER TAX

First elephant	10,000 FCFA
Second elephant	20,000 FCFA
Hippopotamus	10,000 FCFA
Bongo	10,000 FCFA
Situtunga	5,000 FCFA
Hylochère	5,000 FCFA
Leopard or panther	3,000 FCFA
Buffalo	2,000 FCFA
Lion	2,000 FCFA
Yellow-backed duiker	2,000 FCFA
Hippotrague	2,000 FCFA
Magistrate colobus	2,000 FCFA
Bubale	1,000 FCFA
Cob deffassa	1,000 FCFA
Cob de Buffon	1,000 FCFA

C - CAPTURE LICENSE

For protected animals	30,000 FCFA
For protected birds	5,000 FCFA
For unprotected animals	5,000 FCFA
Birds permit valid for 10,000 pairs of unprotected birds	5,000 FCFA

D - ADDITIONAL CAPTURE RIGHTS

Payable when the capture permit is issued for half of the animals listed on the permit.

Mammals

Fully protected mammals:

Elephant (young accompanying his mother and followed female)	50,000 FCFA
Dwarf hippopotamus	40,000 FCFA
Manatee	40,000 FCFA
Chimpanzee	20,000 FCFA

Partially protected mammals

Bongo, Situtunga	20,000 FCFA
All others (except Bubale, Cob de Buffon and Pangolin)	10,000 FCFA
Bubale, Cob de Buffon	5,000 FCFA
Pangolin	2,000 FCFA
Unprotected mammals	1,000 FCFA

BIRDS

Fully protected birds	5,000 FCFA
Partially protected birds	1,000 FCFA
Spectacular birds	200 FCFA

REPTILES

Live reptiles	500 FCFA

E - TAX FOR DETENTION BY INDIVIDUALS OF LIVING ANIMALS IN CAPTIVITY

The tax on the detention by individuals of animals living in captivity is set at 1/5 of the duty complementary catch corresponding. This tax is payable each year and is valid for the year In progress.

F - SLAUGHTER TAX FOR PANTHERS TAKEN IN TRAPS AND WHOSE SKIN IS MARKETED

(Order n ° 1712 AGRI. EFC. Of December 29, 1966, article 4) 3,000 francs.

Article 2:

This decree will be registered and published in the Official Journal of the Republic.

A. SAWADOGO.

Order No. 15 SEPN / SEB of 26 December 1972
amending Decree No. 68 of 23 January 1967 fixing the rates of taxes and fees for hunting and capturing wildlife

THE SECRETARY OF STATE IN CHARGE OF NATIONAL PARKS,

THE BUDGET SECRETARY OF STATE,

Seen to Law No. 65-255 of August 4, 1965, relating to the protection of wildlife and the practice of hunting, in in particular Articles 9, 10 and 17;

Seen the decree n ° 66-423 of September 15, 1966, fixing the hunting license regime and the modalities of their allocation in Côte d'Ivoire, in particular Article 10.

STOP:

First article

The fees collected in application of the hunting regulations on the occasion of the issuance of permits and slaughter taxes are set as follows:

A - HUNTING LICENSE

Small hunting license for milking weapons	1,000 FCFA
Small national hunting license	2,000 FCFA
Medium hunting license	20,000 FCFA
Passenger permit	15,000 FCFA
Big game hunting license	30,000 FCFA

B - SLAUGHTER TAX

First elephant	15,000 FCFA
Second elephant	30,000 FCFA
Hippopotamus	15,000 FCFA
Situtunga	5,000 FCFA
Bongo	20,000 FCFA
Hylochère	5,000 FCFA
Leopard or panther	20,000 FCFA
Buffalo	10,000 FCFA
Lion	20,000 FCFA
Yellow-backed duiker	5,000 FCFA
Hippotrague	10,000 FCFA
Magistrate Colobus	5,000 FCFA
Bubale	10,000 FCFA
Cobe deffassa	10,000 FCFA
Cobe de Buffon	5,000 FCFA
For protected animals	50,000 FCFA

For protected birds	10,000 FCFA
For unprotected animals	10,000 FCFA
Birds permit valid for 1000 pairs of unprotected birds	10,000 FCFA

Article 2:

This decree cancels all previous provisions and in particular Decree No. 68 of 23 January 1967 of paragraphs A, B, and C of its article 1.

Article 3:

This decree will be registered and published in the Official Gazette of the Republic of Côte d'Ivoire.

Secretaries of State in charge of the Budget and National Parks

The Minister of Economy and Finance

Order n ° 003 / SEPN / CAB of February 20, 1974
wearing hunting closure

THE SECRETARY OF STATE IN CHARGE OF NATIONAL PARKS

Seen the decree 71-478 fixing the attributions of the Secretary in charge of National Parks and bearing organization of the State Secretariat;

Seen the decree n ° 6 SEPN / CAB of December 21, 1973 opening the hunt for the 1974 season;

Seen the decree 1615 of December 27, 1965 fixing the opening period of hunting;

Seen to Law No. 65-255 of August 4, 1965, relating to the protection of Nature.

STOPPED :

ARTICLE: 1 - Hunting is closed throughout the Republic of Ivory Coast from the 1st January 1974.

ARTICLE: 2 - The opening of the hunt will be the subject of a subsequent order issued by the Secretary of State in charge of National parks.

ARTICLE: 3 - The Prefects, Sub-Prefects, the Commanders of the Gendarmerie, the Officers of the Police Judicial, the Secretary of State in charge of Reforestation, the Director of Customs, the Director of Nature Protection, Director of National Parks, Heads of Forest Regions and Hunting Inspection Chiefs are each responsible for concerns, the execution of this decree which will be recorded, published and communicated everywhere where needed will be./-

BROADCAST: THE SECRETARY OF STATE

- President / Republic ……………………3
- National Assembly ………………… ... 3
- Economic Council ……………………. 3
- Supreme Court …………………. … .30
- All Ministries ……………………..…. 30
- Ministry of the Interior ……………..119
- Ministry of Agriculture ………………5

K. ATTOBRA.

- Reforestation ………………………… 119
- A.I.P. ……………………………. ……… .2
- Commander Gendarmerie …………….. .119
- Fraternity-Morning ………………………. 3
- Weekly ………………….. .. …… …..….3

- National Parks …………………………..…. .30
- Nature Protection Department ……… ..… ..30
- Customs Department ………………… ..… .30
- National Security Department ………… ..… 30
- J.O.C.I. …………………………… ... …… .3
