# Supplementary material for: Ivory Coast without ivory: Massive extinction of African forest elephants in Côte d’Ivoire
Source: PLoS One. 2020 Oct 14;15(10):e0232993. doi: 10.1371/journal.pone.0232993 (PMC7556483; doi:10.1371/journal.pone.0232993)
Supplement: Attachment — Submitted filename: Ivory Coast elephants.pdf [file pone.0232993.s010.pdf]

This is the most depressing paper I've read in years. I cannot imagine how much worse it must have been for the authors to do all these surveys and find no evidence of elephants in so many of the areas. That said, this is an important survey and deserves publication.

My only concerns are with the English. Yes, I know this is likely a second or third language for the authors. I think they've done a good job, but they need to use the tools available to them. Word itself provides suggestions, but Grammarly is excellent and I use it extensively. I've made some suggestions here — I cannot do a complete edit, so I hope these will give some idea of what's needed.

The main problems are the excessive use of the passive voice and often very long sentences.

In pre-colonial and colonial times Co⍰te d'Ivoire probably hosted one of the largest elephant populations in West Africa, resulting in the country's English name Ivory Coast. An estimated number of 1790 individuals' savannah elephant and 3050 of forest elephants was reported. Numbers declined further and by the early 1990s it was estimated that the total number of savannah and forest elephants had fallen to only 63 to 360 individuals respectively in the entire country. Here, we present updated information on the distribution and conservation status of forest elephant in Co⍰te d'Ivoire based on multiple sources. We used dung counts along line transects, records of human–elephant conflict, media reports, sign and interview surveys during the period 2011–2017.

Deleted: —Co⍰te d'Ivoire

Commented [MOU1]: By whom? And when?

Commented [MOU2]: Passive voice.

Deleted: on

Correlation between the presence of forest elephant and site variables (size of the forest, percentage of area converted into plantation, size of the forest left, size of human population inside the PA, poaching index, distance to the nearest road, population density in the Department, level of protection of the PA) was investigated with Pearson correlation. To examine the effect of ecological traits on elephant extirpation, we used Principal Components Analysis (PCA) to check for multicollinearity among variables. Based on dung count we confirmed elephant presence in only 5 of the 25 protected areas surveyed. Of the 25 PAs surveyed, 20 (80 %) have lost their entire population of forest elephant. PAs with higher level of protection have higher probability of having elephants. The viability of these populations is uncertain. Their size is much reduced and their populations are isolated. Aggressive conservation actions including law enforcement for the protection of their remaining habitat and anti-poaching actions are needed to protect the remaining forest elephant populations.

Commented [MOU3]: Passive voice — and a sentence that's too long.

Commented [MOU4]: You don't need to say this twice

Deleted: questionable

Introduction

Africa is home to at least 400,000 elephants, an estimated 5,458 of which are found in West Africa [1]. While some genetic evidence suggests that the West African elephant is a distinct species [2]. Others argue that there is insufficient evidence to prove this hypothesis [3] and encourage further research.

Among the large mammals of Africa, elephant is probably one of the most affected by human activities. The greatest threat to them in West Africa comes from habitat loss due to human encroachment [4]. Elephant habitat in West Africa has been fragmented, most of which are subjected to significant pressures from human population growth, the spread of agriculture, and livestock production surrounding the parks [4].

In pre-colonial times Co⍰te d'Ivoire probably hosted one of the largest elephant populations in West Africa Despite the early harmful effects of human activities, elephant numbers remained high until

Deleted: elephants

Deleted: ecosystem changes related to

Deleted: into small habitats

Deleted: in terms of the growth of

Deleted: numbers

Deleted: negative

colonial time in West Africa, resulting in the country's English name — Ivory Coast. Given by the colonial powers to this area in West Africa it was richest in elephants (or at least elephant tusks) [5]. However, during the three last decades, elephant populations have been sharply reduced, mainly because of forest agricultural clearing [6]. Intensive poaching poses a further threat, especially to larger bodied-sized species. An estimated total population of 1790 individuals in savannah and 3050 forest elephants scattered throughout Co�te d'Ivoire was estimated [7].

Numbers declined further by the late 1980s when [8] presented data on the remaining 20 isolated populations of forest elephants. There was a further 50 % decline in numbers. The early 90s saw even further declines when the total number of both savannah and forest elephants dropped to 63 to 360 individuals respectively, in 24 populations across the entire country [1]. These populations are confined to protected areas and continue to decline. Indeed, at the outset of the 20th century, there were sixteen million hectares of high canopy forest in Co�te d'Ivoire. Today, that number is four million ha and declining due to an annual deforestation rate of approximately 1 % [9, 10, 11, 12]. The remaining forest of Co�te d'Ivoire is highly fragmented and largely consists of nominally protected national parks and forest reserves. Wildlife in these protected areas is threatened by hunting, the encroachment of cocoa plantations on reserve borders, and expansion of illegal cocoa farming within the parks and reserves themselves [13, 14, 15, 16].

Elephant numbers in Co�te d'Ivoire have decreased at an alarming rate since the first population surveys were conducted. Warnings that this species might be lost were put forward in 1984 [17]. The situation is probably even worse, with only seven elephant populations confirmed in the last decade, comprising approximately 270 elephants [17]. The outbreak of civil war in Co�te d'Ivoire in September 2002 harmed the management of certain protected areas and conservation in general [18]. Although not quantified, it can be assumed that the effect on the small population of elephants was harmful if not catastrophic [17]. Within the scope of the national strategy of elephant conservation in Co�te d'Ivoire, a need to survey the main habitats for elephants and the identification of viable population was recommended [19]. The most recent data on the population of Co�te d'Ivoire elephants are at least one decade ago and most of these data did not follow a standardized protocol. The aim of this paper is to get updated information on the distribution and conservation status of the forest elephants of Co�te d'Ivoire.

Methods Study sites

We used historical distribution data and information from elephant surveys to identify twenty- six protected areas (PA) in central and southern Co�te d'Ivoire known to contain elephants (Fig 1). The surveyed localities belong to the Guinean forest zone below the V-Baoule� in the southern part of Co�te d'Ivoire. Protected areas are categorized into National Parks, which normally are under strong protection restriction, and Forest Reserves that are partially protected. We selected the PAs to be surveyed based on the presence of forest habitat. The exceptions were Marahoue� National Park and Koba Forest Reserve in central Co�te d'Ivoire. These both consist of both dense forest and savanna woodland.

Prior to the surveys, we received research permits from SODEFOR (Society of Forest Development) and OIPR (Ivorian Office of Parks and Reserves), respectively in charge of the management of Co�te d'Ivoire's forest reserves and parks. SODEFOR and OIPR reviewed and approved the study was reviewed

Assessment of forest elephant distribution

Deleted: name
Deleted: —Co�te d'Ivoire—g
Deleted: the
Deleted: that
Deleted: and i
Commented [MOU5]: Passive voice and say when
Deleted: with
Deleted: , and even further by t
Deleted: had reached
Deleted: elephants
Deleted: existing
Deleted: in
Deleted: that are continuously on
Deleted: an estimated
Deleted: existed
Deleted: .
Commented [MOU6]: Passive voice
Deleted: old
Deleted: two items —
Deleted: f
Deleted: r
Deleted: T
Deleted: were originally selected
Deleted: , excepted
Deleted: ,
Deleted: which
Deleted: T
Deleted: and approved by
Deleted: SODEFOR and OIPR.

From 2011–2017, we collected data on elephant occurrence from multiple sources including dung counts along line transects, records of human-elephant conflict, media reports, sign and interview surveys. We also collected data on elephant distribution through structured interviews. Interviews were with key informants from the surrounding local communities of PAs as well as field staff of the Forest and Wildlife Department. Before the interview, we explained the research purpose to the informants. We received a verbal consent of their acceptance to participate to the study. We gathered data on locations of elephant from news reports from newspapers and Internet searches for verifiable news reports of elephant presence in localities close PAs where elephants' presence was confirmed. We also talked to researchers who conducted scientific or conservation work in these areas and others. We focused mainly on dung counts along line transects carried out across eight surveyed sites (Dassioko Sud FR, Port Gauthier FR, Monogaga FR, Nie□gre□ FR, Okroumodou FR, Azagny NP, Bolo Ouest, Marahoue□ NP) during the wet period (from April to July) and the dry one (from December to March) of 2011 to 2017.

Deleted: W

Deleted: on

Deleted: obtained during from 2011–2017

Deleted: D

Deleted: were also collected

«Insert Fig 1»

We made efforts to place line-transects within each forest where elephants were known to occur. The field protocol involved surveyors in a team of two persons walking once along each transect of 2-9 km length. We recorded elephant dung piles when encountered, their number, and the perpendicular distance from transect to the center of the dung piles in addition to data on vegetation type [20, 21]. These data were used to infer elephant distribution but also to compute dung densities for each transect (in dung piles/ha), which we treated as an index of elephant density.

Deleted: E

Deleted: were made

During surveys, we also recorded all signs of poaching (e.g., number of gunshots heard, snares, discarded cartridges, etc.) and used these to calculate a poaching index, which is defined as the number of poaching signs per kilometer walked in each PA.
