# Supplementary material for: Ivory Coast without ivory: Massive extinction of African forest elephants in Côte d’Ivoire
Source: PLoS One. 2020 Oct 14;15(10):e0232993. doi: 10.1371/journal.pone.0232993 (PMC7556483; doi:10.1371/journal.pone.0232993)
Supplement: Attachment — Submitted filename: Response to reviewers_27aout20.docx [file pone.0232993.s011.docx]

**‘PONE-D-20-09118
Ivory Coast without ivory: massive extinction of African forest elephants in Côte d’Ivoire**

**Journal requirements:**

When submitting your revision, we need you to address these additional requirements.

1. Please ensure that your manuscript meets PLOS ONE's style requirements, including those for file naming. The PLOS ONE style templates can be found at <https://journals.plos.org/plosone/s/file?id=wjVg/PLOSOne_formatting_sample_main_body.pdf> and <https://journals.plos.org/plosone/s/file?id=ba62/PLOSOne_formatting_sample_title_authors_affiliations.pdf>

Adressed

2. Please include additional information regarding the survey or questionnaire used in the study and ensure that you have provided sufficient details that others could replicate the analyses. For instance, if you developed a questionnaire as part of this study and it is not under a copyright more restrictive than CC-BY, please include a copy, in both the original language and English, as Supporting Information.

Added as supporting information

3. In your Methods section, please state where the people interviewed were recruited for your study.

The people interviewed were recruited among local communities in the localities surrounding the surveyed PAs.

4. Thank you for stating the following in your Ethics Statement that "Prior to the surveys, we received research permits from SODEFOR (Society of Forest Development) and OIPR (Ivorian Office of Parks and Reserves), respectively in charge of the management of Côte d’Ivoire’s protected areas. The study was reviewed and approved by SODEFOR and OIPR."

We noted that in your methodology you report that "Data on elephant distribution were also collected through structured interviews. Interviews with key informants from the surrounding local communities of PAs as well as field staff of the Forest and Wildlife Department" (lines 107-110).

For research involving human participants, such as conducting face-to-face interviews, we would expect approval from your institutional review board (IRB) or equivalent ethics committee(s) and reporting of participant consent. We would like to know whether all the research reported in your manuscript, in particular the work with human participants, has been performed at all times with ethical oversight by an ethics committee. Could you please clarify if your research permits included ethics oversight for conducting the face-to-face interviews?

If ethical approval was not required, please provide a clear statement of this and the reason why, and any relevant regulations under which the work is exempt from the requirement for approval. If the ethics approval was waived by your ethics committee, please provide a copy of this documentation formally confirming that ethical approval was not needed in this case, in the original language and in English translation as supporting information files. Please note that an email from your ethics committee will suffice. Please note that this is for internal use only and will not be published.

Ethical approval in our country is not necessarily required for study dealing with animals. Indeed in appendix I of the law n ° 94-442 of August 16, 1994 amending the law n ° 65-255 of August 4, 1965 relating to the protection of fauna and hunting practices, fully protected wild animals including the elephant are prohibited from hunting and capture. Our study fully respected Ivorian regulations in force because it did not in any way affect the integrity of elephants nor the disturbance of that integrity. The authorizations issued by the authorities in charge of the protection of protected areas serve as their agreement regarding the regulations in force in our country.

In addition, please provide additional details regarding participant consent. In the ethics statement in the Methods and online submission information, please ensure that you have specified (1) whether consent was informed and (2) what type you obtained (for instance, written or verbal). If the need for consent was waived by your ethics committee, please include this information.

Before the interview, we explained the research purpose to the informants. We received a verbal consent of their acceptance to participate in the study.

Thanks for your attention to our requests.

5. Please upload a new copy of Figure 2 as the detail is not clear. Please follow the link for more information: <https://blogs.plos.org/plos/2019/06/looking-good-tips-for-creating-your-plos-figures-graphics/>" <https://blogs.plos.org/plos/2019/06/looking-good-tips-for-creating-your-plos-figures-graphics/>

Done

6. Please include captions for your Supporting Information files at the end of your manuscript, and update any in-text citations to match accordingly. Please see our Supporting Information guidelines for more information: http://journals.plos.org/plosone/s/supporting-information.

Reviewers' comments:

Reviewer's Responses to Questions

**Comments to the Author**

1. Is the manuscript technically sound, and do the data support the conclusions?

The manuscript must describe a technically sound piece of scientific research with data that supports the conclusions. Experiments must have been conducted rigorously, with appropriate controls, replication, and sample sizes. The conclusions must be drawn appropriately based on the data presented.

Reviewer #1: Yes

Reviewer #2: Partly

2. Has the statistical analysis been performed appropriately and rigorously?

Reviewer #1: Yes

Reviewer #2: No

3. Have the authors made all data underlying the findings in their manuscript fully available?

The [PLOS Data policy](http://www.plosone.org/static/policies.action#sharing) requires authors to make all data underlying the findings described in their manuscript fully available without restriction, with rare exception (please refer to the Data Availability Statement in the manuscript PDF file). The data should be provided as part of the manuscript or its supporting information, or deposited to a public repository. For example, in addition to summary statistics, the data points behind means, medians and variance measures should be available. If there are restrictions on publicly sharing data—e.g. participant privacy or use of data from a third party—those must be specified.

Reviewer #1: Yes

Reviewer #2: Yes

4. Is the manuscript presented in an intelligible fashion and written in standard English?

PLOS ONE does not copyedit accepted manuscripts, so the language in submitted articles must be clear, correct, and unambiguous. Any typographical or grammatical errors should be corrected at revision, so please note any specific errors here.

Reviewer #1: No

Reviewer #2: Yes

 5. Review Comments to the Author

Please use the space provided to explain your answers to the questions above. You may also include additional comments for the author, including concerns about dual publication, research ethics, or publication ethics. (Please upload your review as an attachment if it exceeds 20,000 characters)

**Reviewer #1: See attached**

I've attached my review as indicated.

We made corrections as indicated by the reviewer.

**Reviewer #2: Questions/Issues**

1) How did you differentiate forest from savanna elephant dung or are you just assuming that there are no savanna elephants in the study area?

The PAs surveyed extend in the southern zone of Côte d’Ivoire, the historical range of forest elephants, thus we assume that the population surveyed are forest elephants.

2) Did you correct for the number of correlations run and the likely lack of independence between some of your variables? I also question some of your signs and p-values for your correlations; it would be helpful to see the sample sizes.

The correlation between the presence of elephant and site variables (size of the forest, percentage of area converted into plantation, size of the forest left, size of human population inside the PA, poaching index, distance to the nearest road, population density in the Department, level of protection of the PA) was evaluated with Pearson correlation. These variables were log transformed as needed to improve normality. In total 25 PAs were surveyed.

3) I’m confused by the locations where elephants were found. The abstract indicates in 5 PAs (34). The results state 5 PAs and 3 unprotected areas (166). The conclusion states 4 PAs (384) and says nothing about the unprotected areas.

Elephants were found in 4 PAs and 3 unprotected areas. This has been corrected in the document.

4) The Discussion is overly long. The paper becomes a history lesson and a review. There are 7 pages of discussion compared to two pages of introduction. While it is preferred to have a longer discussion than introduction, much of the discussion goes well beyond the data of the paper. It could easily be trimmed by several pages by reducing repetition and tightening the message. The 96 citations is perhaps a bit excessive for the depth of the article.

The discussion section has been reduced. Efforts were made to reduce repetition. Citations have been reduced to 57.

Minor Edits
22 delete “further” – since no numbers are given in 19-20 it is impossible for the reader to know if the value provided in 21 are “one of the largest” or a decline.

Done

24 The authors should say something about savanna elephants given the previous lines

As the study focus on forest elephant we think that there is no need to say more on savannah elephant in the abstract.

31 You do not investigate with a statistical test – reword

We used Pearson correlation to determine the correlation between the presence of forest elephant and site variables

36 Reword – improper grammatically – the Pas are not inhabiting elephants

PAs with higher level of protection have higher probability to be home of elephant population.

37 Reword “largely reduced”
The viability of these populations is uncertain, since they have a small size and are isolated.

39 What are antipoaching actions?
Antipoaching actions refer here to ranger patrolling

46 They are two species (<https://www.nature.com/articles/news.2010.691>; <https://news.mongabay.com/2018/08/forest-elephant-dna-diverse-consistent-and-distinct-study-says/>; <https://www.pnas.org/content/115/11/E2566>; <https://will.illinois.edu/longerlisten/story/the-african-elephant-is-actually-two-separate-species-and-in-danger>; <https://www.nature.com/articles/ng1485>) any debate is political / legislative and not based on scientific evidence.

Genetic evidence suggests two distinct species among African elephants: the forest elephant (*Loxodonta cyclotis*) and the savanna elephant (*L. africana*).

48 either “the elephant is” or “elephants are”
Corrected

55 missing a period

In pre-colonial times (before 1893)

58 last three
Corrected

60 delete “poses a further threat” – unnecessary
Deleted

67 in decline

These populations are confined to protected areas and continue to decline

79 delete “has”
Deleted

83 change “of” to “for”
Done

84 populations
Corrected

86 change “paper” to “study”
Done

87 change “get” to “obtain”

Done

95 place a colon after “items” and delete “firstly”

Protected areas are categorized into National Parks which normally are under strong protection restriction, and Forest Reserves that are partially protected.

96 place comma after “Parks” and after “restriction”

Done

106 unclear if you mean “sign,” such as indicators of elephants, or “sign and interview surveys” in which case I am not sure of the meaning of “sign surveys” – use the Oxford comma if the former and explain if the latter

sign, and interview surveys… Sign as indicators of elephants

109 Interviews were conducted with …

Corrected

112 “in the study” and “elephants in” (delete “from”)
Done

114 “close to”

Corrected

115 what’s the relevance of talking to researchers who do work at “other” places – clarify (e.g., bordering areas, etc.)
You may wish to look at this reference too: Youldon et al. 2017. Patch - occupancy survey of elephant (Loxodonta africana) surrounding Livingstone, Zambia. Koedoe, 59(1), a1372. <https://doi.org/10.4102/koedoe.v59i1.1372>.

We also talked to researchers who conducted scientific or conservation work in these areas and the bordering areas

136 You’ve already presented the (PA) so just use the PA

Done

145 Again, you do not investigate with a statistical test

………..was evaluated with Pearson correlation

168 change “are” to “were”

Done

186-8 Were both correlations positive? It would seem that elephant presence and poaching index would be opposite in sign to presence of elephants and level of protection of the PA. In 132 you define the poaching index such that the higher the sign of poaching the higher the index value.

Our data indicated no significant correlations between the presence of elephants in the PAs and poaching index (r = 0.223, p = 0.283). This could be explained by the fact that all the poaching signs are not necessarily targeting elephants.

191 So as the presence of elephants went up, the density went down (yet there was no correlation with size of the PA) …seems odd.

There is no correlation between the presence of elephant and the size of the PA. As well as the PA has a large size, once there is no protection and that the habitat of that PA is degraded, there is less chance to find elephants.

193-5 The lack of a significant correlation for these two measures is interesting and perhaps unexpected.

The lack of significant correlation between the presence of elephants and the distance to the nearest road (r = - 0.176, p = 0.399) or the size of the PA (r = 0.217, p = 0.298) is effectively unexpected and indicates the level of disturbance that are facing the surveyed PAs.

217 Reword – see previous comment about this structure

Done

234 insert “and” before “increasing”

Done

239 I’m not sure what “conducting to the illegal plantation” means
Conversion of protected areas into plantations

242 Do you mean “risen”?

Yes

299 change “does” to “do”

Done

330 Use Oxford comma if journal permits

Not changed, to lengthening the sentence

Continue to check grammar / sentence structure / word use through the end of the manuscript.

We make effort to check grammar / sentence structure / word use through the end of the manuscript.

6. PLOS authors have the option to publish the peer review history of their article ([what does this mean?](https://journals.plos.org/plosone/s/editorial-and-peer-review-process#loc-peer-review-history)). If published, this will include your full peer review and any attached files.

If you choose “no”, your identity will remain anonymous but your review may still be made public.

**Do you want your identity to be public for this peer review?** For information about this choice, including consent withdrawal, please see our [Privacy Policy](https://www.plos.org/privacy-policy).

Reviewer #1: No

Reviewer #2: No


[NOTE: If reviewer comments were submitted as an attachment file, they will be attached to this email and accessible via the submission site. Please log into your account, locate the manuscript record, and check for the action link "View Attachments". If this link does not appear, there are no attachment files.]

While revising your submission, please upload your figure files to the Preflight Analysis and Conversion Engine (PACE) digital diagnostic tool, <https://pacev2.apexcovantage.com/>. PACE helps ensure that figures meet PLOS requirements. To use PACE, you must first register as a user. Registration is free. Then, login and navigate to the UPLOAD tab, where you will find detailed instructions on how to use the tool. If you encounter any issues or have any questions when using PACE, please email PLOS at [figures@plos.org](mailto:figures@plos.org). Please note that Supporting Information files do not need this step.

Done
